# Inverse-Sandwich Rare Earth Metal Complexes Comprising a Planar Toluene Dianion

**DOI:** 10.1021/acs.inorgchem.5c04418

**Published:** 2026-01-03

**Authors:** Elizabeth R. Pugliese, Saroshan Deshapriya, Mackenzie Meyer, Ernesto Castellanos, Selvan Demir

**Affiliations:** Department of Chemistry, 3078 Michigan State University, 578 South Shaw Lane, East Lansing, Michigan 48824, United States

## Abstract

We report the first inverse-sandwich complexes containing rare earth (RE^III^) metal ions that captured a toluene dianion between them. Toluene-bridged complexes [{(Me_3_Si)_2_NC­(N^i^Pr)_2_}_2_RE]_2_(μ-η^6^:η^6^-C_6_H_5_Me) (RE = Y (**1**), Dy (**2**), and Er (**3**)) were synthesized via chemical reductions of chloride-bridged RE complexes in which each tripositive metal is stabilized by two guanidinate ligands. Compounds **1–3** were unambiguously characterized by crystallography, NMR, UV–vis, and IR spectroscopy, magnetometry, and computations. The bond metrics from single-crystal X-ray diffraction analysis revealed a planar, cyclohexadienediide-like structure for the ligated arene, indicative of a dianionic toluene. The ^1^H NMR spectrum of **1** exhibits upfield-shifted resonances representing increased shielding from excess electrons, further validating its dianionic nature. DFT calculations afforded similar bond metrics, and natural bond orbital (NBO) analysis uncovered ionic bonding interactions between the bridging toluene and the yttrium centers, supporting the assignment of a −2 charge to the toluene. UV–vis spectroscopy highlighted that the electronic excitations primarily stem from toluene- and guanidinate-based orbitals. The Dy and Er congeners were further probed by SQUID magnetometry, with **3** revealing weak magnetic exchange coupling between the Er^III^ centers. These findings highlight the ability of reduced arenes to serve as bridging ligands in multimetallic rare earth architectures.

## Graphical abstract

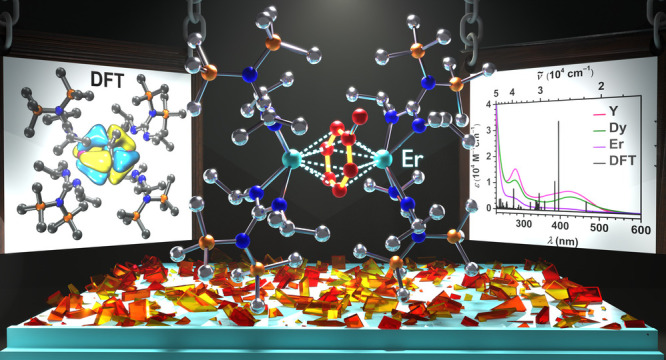

## Introduction

Since the landmark discovery of bis­(benzene)­chromium, Cr­(η^6^-C_6_H_6_)_2_, in 1956,^1^ the isolation of metal–arene interactions has continued to attract a significant amount of scientific interest. Notably, metal–arene interactions have enabled the isolation of low-valent oxidation states in rare earth (RE) metal chemistry^2–6^ , unusual electronic structures,^7^ and have found applications in catalysis^8,9^ as well as in fundamental organometallic chemistry.^10–12^ While metal–arene compounds can be isolated with RE and transition metals (TM), they differ in the nature of their metal–ligand bonding interactions. Specifically, TMs can invoke covalent metal–arene bonds through the combination of metal d orbitals and ligand σ, π, or π* orbitals. This has allowed for the isolation of TM complexes bearing neutral aromatic molecules in various coordination geometries.^3,6,13^ Examples of RE metal compounds containing neutral arene ligands were first isolated as zerovalent mononuclear sandwich complexes featuring substituted benzene ligands.^3,6,13^ However, supported RE–arene interactions have been realized through the implementation of bulky ligand scaffolds, such as the bis­(terphenylamide) framework^4,5,14,15^ or the weakly coordinating [BPh_4_]^−^ anion,^11,16,17^ where the ligand framework is anionic, with the charge not residing on the arene moiety.

Since RE metal ions primarily promote ionic bonding interactions, though polar covalent bonds between Y and heavy p-block elements have been realized,^18^ there are fewer examples of organometallic RE complexes comprising unsupported neutral arene molecules.^19^ As such, RE ions tend to form inverse-sandwich complexes with reduced arenes. In an inverse-sandwich complex, an arene ligand bridges two metal ions. Notably, benzene, as the simplest bridging arene, has been stabilized as mono-,^20^ di-,^7,21,22^ and tetraanions^23–25^ with a number of RE metals. The varying degree of anionic charge on the benzene bridge was achieved through reductive chemical conditions such as the use of reductants^22–25^ or reactive metal–metal bonds^26^ to overcome the large reduction potential for benzene of −3.42 V versus SCE.^27^ The formation of such inverse-sandwich RE complexes additionally necessitates suitable ancillary ligands ligated to the metals with sufficient steric bulk and stabilizing effects. Our group discovered that a bis­(guanidinate) scaffold around tripositive lanthanides allows the stabilization of a remarkably planar benzene dianion.^22^

Intriguingly, inverse-sandwich RE complexes containing functionalized arenes, such as toluene, are scarce (Figure 1 and Figure S1).^10,19,25,28–30^ Specifically, low-valent TM and RE metals have afforded inverse-sandwich complexes with neutral bridging toluene molecules (Figure 1A), whereas activation of a tetraanionic bridging toluene ligand was stabilized through a bulky amidinate framework with RE^III^ ions (Figure 1B). Among the versions containing RE^III^ ions, the dianonic charge state of toluene remains elusive. Relative to benzene, the methyl group of toluene causes (I) a decrease in symmetry leading to fewer equivalent C and H atoms within the arene ring, (II) inductive effects and hyperconjugation via overlap of methyl C–H bonds with the π system,^31^ and (III) a perturbation of the π system where the degeneracy of frontier molecular orbitals is lifted while the π and δ symmetry of these orbitals remain intact (Figure 1D; see the experimental details).

**Figure 1:**
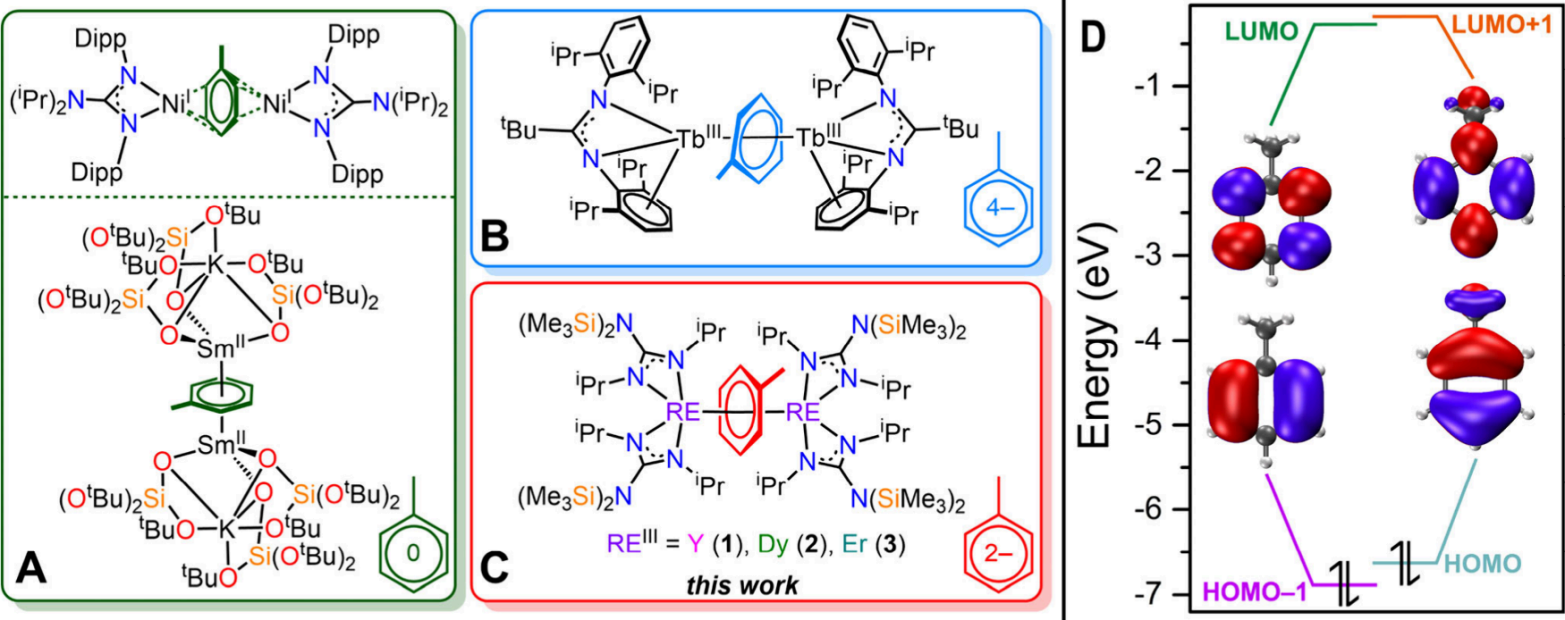
Select examples of crystallographically confirmed toluene-bridged inverse-sandwich complexes: (A) [{(^i^Pr)_2_NC­(N­(Dipp))_2_}_2_Ni]_2_(μ-η:^3^η^3^-C_6_H_5_Me) and [{KSm­(OSi­(O^t^Bu)_3_)_3_}_2_(μ-η^6^:η^6^-C_6_H_5_Me)], where each complex features a neutral toluene bridge; (B) [{Tb­(κ^1^:η^6^-Piso)}_2_(μ-η^6^:η^6^-C_6_H_5_Me)], which exhibits a tetranionic toluene bridge; and (C) [{(Me_3_Si)_2_NC­(N^i^Pr)_2_}_2_RE]_2_(μ-η^6^:η^6^-C_6_H_5_Me), where RE = Y (**1**), Dy (**2**), and Er (**3**), which contains a dianionic toluene bridge. (D) Frontier molecular orbitals calculated for a neutral, free toluene molecule. HOMO–1 and HOMO contain one nodal plane in the arene ring system, while LUMO and LUMO+1 comprise two nodal planes in the ring. The isovalue used for molecular orbital representation is 0.03. See the experimental details. Abbreviations: Dipp, 2,6-(^i^Pr)_2_C_6_H_3_; Piso, {N­(2,6-(^i^Pr)_2_C_6_H_3_)}_2_C^t^Bu.

Inspired by the developed guanidinate chemistry in our group, which allowed access to halide-,^32^ radical-,^33^ and benzene-bridged RE complexes,^22^ we set out to explore the possibility of capturing and reducing toluene with tripositive RE ions supported by a bis­(guanidinate) scaffold under reductive chemical conditions. Herein, we report the synthesis and characterization of the first series of inverse-sandwich complexes of trivalent RE ions bridged by a toluene dianion, [{(Me_3_Si)_2_NC­(N^i^Pr)_2_}_2_RE]_2_(μ-η^6^:η^6^-C_6_H_5_Me) (where RE = Y (**1**), Dy (**2**), and Er (**3**)). Compounds **1**–**3** represent structurally authenticated toluene-bridged dinuclear complexes of yttrium, dysprosium, and erbium, respectively, in any oxidation state of toluene. A standout feature constitutes the use of ancillary guanidinate scaffolds on the metal centers.

## Experimental Section

### General Information

All manipulations were performed in an argon-filled MBraun glovebox with an atmosphere of <0.1 ppm O_2_ and <0.1 ppm H_2_O. Toluene was dried by refluxing over potassium for several days. Diethyl ether was dried by refluxing over a Na/K alloy. *n*-Hexane and *n*-pentane were dried by refluxing over calcium hydride. All solvents were distilled under argon and placed in an argon-filled glovebox, where the solvents were tested for the presence of water and oxygen by the addition of one drop of a potassium benzophenone radical solution to 2 mL of the solvent of interest. YCl_3_, DyCl_3_, and ErCl_3_ were purchased from Sigma-Aldrich and used as received. *N,N*′-Diisopropylcarbodiimide was purchased from Alfa-Aesar and dried over 4 Å molecular sieves prior to use. Toluene-*d*_8_ was purchased from Sigma-Aldrich, dried over a Na/K alloy, and filtered prior to use. Lithium bis­(trimethylsilyl)­amide, Li­[N­(SiMe_3_)_2_], was purchased from Sigma-Aldrich, dissolved in toluene, filtered through a Celite plug, dried, and recrystallized from toluene at −35 °C. The lithium salt of the *N,N*′-diisopropyl-*N*″-bis­(trimethylsilyl)­guanidinate anion, Li­(Me_3_Si)_2_NC­(N^i^Pr)_2_, was synthesized through the addition of Li­[N­(SiMe_3_)_2_] to an *n*-hexane solution of *N,N*′-diisopropylcarbodiimde following a literature procedure.^34^ KC_8_,^35^ [{(Me_3_Si)_2_NC­(N^i^Pr)_2_}_2_Y­(μ-Cl)]_2_,^36^ [{(Me_3_Si)_2_NC­(N^i^Pr)_2_}_2_Dy­(μ-Cl)]_2_,^37^ and [{(Me_3_Si)_2_NC­(N^i^Pr)_2_}_2_Er­(μ-Cl)]_2_ ^38^ were synthesized according to literature procedures.

**Caution!** *KC_8_ is corrosive and extremely pyrophoric under ambient conditions. All manipulations were performed in an argon-filled MBraun glovebox with an atmosphere of <0.1 ppm O_2_ and <0.1 ppm H_2_O, and on a small practical scale following the procedures described below*.

### Synthesis of [{(Me_3_Si)_2_NC­(N^i^Pr)_2_}_2_Y]_2_(μ-η^6^:η^6^-C_6_H_5_Me) (**1**)

103.1 mg (0.0739 mmol) of [{(Me_3_Si)_2_NC­(N^i^Pr)_2_}_2_Y­(μ-Cl)]_2_ was dissolved in 3 mL of diethyl ether in a 20 mL scintillation vial charged with a magnetic stir bar, forming a clear, colorless solution. Then, 100 equivs of toluene (7.39 mmol, 0.78 mL) were added via syringe to the reaction vessel. KC_8_ (69.9 mg, 0.517 mmol, 7.0 equiv) was added, and the reaction was allowed to proceed at room temperature. After 18 h, the resulting yellow-orange reaction mixture was filtered through a Kimwipe plug into a clean 20 mL scintillation vial. The yellow-orange filtrate was evaporated to dryness under reduced pressure, yielding an orange oily residue. The resulting orange residue was extracted in 15 mL of *n*-pentane and filtered over a Celite plug into a clean 20 mL scintillation vial. The resulting bright orange solution was evaporated to dryness. The solids were subsequently dissolved in *n*-pentane and filtered over a Celite plug. Orange block-shaped crystals of **1** suitable for single-crystal X-ray diffraction analysis were grown from the slow evaporation of the pentane solution at room temperature in a 33% crystalline yield (34.3 mg, 0.0242 mmol). The crystalline material degrades within 10 min under an ambient atmosphere, even when covered in Paratone oil. IR (FTIR, cm^–1^): 2959m, 2923w, 2902w, 2869w, 1636w, 1453s, 1357m, 1319s, 1249s, 1190s, 1128m, 1041s, 939s, 880m, 822s, 753m, 679s, 657s. Anal. Calcd for C_59_H_136_N_12_Si_8_Y_2_: C, 50.03; H, 9.68; N, 11.87. Found: C, 49.61; H, 9.48; N, 11.87.

### Synthesis of [{(Me_3_Si)_2_NC­(N^i^Pr)_2_}_2_Dy]_2_(μ-η^6^:η^6^-C_6_H_5_Me) (**2**)

Following the analogous synthetic procedure for **1**, red crystals of **2** were grown from the slow evaporation of a pentane solution at room temperature in a 46% crystalline yield (42.0 mg, 0.0269 mmol). Dy exhibited a more intense red color in comparison to the bright orange of yttrium and erbium in all of the steps described for the synthesis of **1**. Used masses: [{(Me_3_Si)_2_NC­(N^i^Pr)_2_}_2_Dy­(μ-Cl)]_2_ (89.3 mg, 0.0.579 mmol), toluene (5.79 mmol, 0.62 mL, 100 equiv), KC_8_ (54.6 mg, 0.404 mmol, 7.0 equiv). IR (FTIR, cm^–1^): 2959m, 2922w, 2901w, 2868w, 1636w, 1451s, 1357m, 1318s, 1249s, 1169m, 1189s, 1132m, 1041s, 939s, 880m, 821s, 754s, 679s, 656s. Anal. Calcd for C_59_H_136_N_12_Si_8_Dy_2_: C, 45.32; H, 8.77; N, 10.75. Found: C, 44.89; H, 8.63; N, 10.74.

### Synthesis of [{(Me_3_Si)_2_NC­(N^i^Pr)_2_}_2_Er]_2_(μ-η^6^:η^6^-C_6_H_5_Me) (**3**)

Following the analogous synthetic procedure for **1**, orange crystals of **3** were grown from the slow evaporation of a pentane solution at room temperature in a 59% crystalline yield (56.0 mg, 0.036 mmol). Used masses: [{(Me_3_Si)_2_NC­(N^i^Pr)_2_}_2_Er­(μ-Cl)]_2_ (93.2 mg, 0.060 mmol), toluene (6.006 mmol, 0.64 mL), KC_8_ (56.8 mg, 0.420 mmol, 7.0 equiv). IR (FTIR, cm^–1^): 2961m, 2922w, 2902w, 2869w, 1637w, 1452m, 1358m, 1321m, 1250s, 1190s, 1169m, 1133m, 1042s, 938s, 880w, 823s, 754m, 679s, 657m. Anal. Calcd for C_59_H_136_N_12_Si_8_Er_2_: C, 45.05; H, 8.71; N, 10.69. Found: C, 44.68; H, 9.00; N, 10.80.

### Single-Crystal X-ray Diffraction Analysis

Light orange, light red, and dark orange crystals of **1–3**, respectively, with dimensions of 0.377 mm × 0.242 mm × 0.116 mm, 0.09 mm × 0.07 mm × 0.02 mm, and 0.122 mm × 0.066 mm × 0.058 mm, respectively, were mounted on a nylon loop using Paratone oil. Data for **1**–**3** were collected on an XtaLAB Synergy, Dualflex, HyPix diffractometer equipped with an Oxford Cryosystems low-temperature device, operating at 100.00(10), 100.00(11), and 100.00(11) K for **1**–**3**, respectively. Data for **1**–**3** were measured using ω scans using Cu Kα radiation (microfocus sealed X-ray tube, 50 kV, 1 mA). The total number of runs and images was based on the strategy calculation from CrysAlisPro (Rigaku, version 1.171.41.90a, 2025),^39^ which was used to retrieve and refine the cell parameters, as well as for data reduction. A numerical absorption correction based on Gaussian integration over a multifaceted crystal model empirical absorption correction using spherical harmonics was implemented in the SCALE3 ABSPACK scaling algorithm. The structures for **1**–**3** were solved in the *P*1̅ space group by using the Intrinsic Phasing ShelXT^40^ structure solution program. The structures were refined by least squares using version 2018/2 of XL^41^ incorporated in Olex2.^42^ All non-hydrogen atoms were refined anisotropically. Hydrogen atom positions were calculated geometrically and refined by using the riding model.

### NMR Spectroscopy

NMR spectra were recorded on a Bruker Avance III HD 500 MHz NMR spectrometer and calibrated to the residual solvent signals (toluene-*d*_8_, δ_H_ 2.09, δ_C_ 137.86). Signal multiplicities are abbreviated as s (singlet), d (doublet), t (triplet), q (quartet), and m (multiplet). Owing to the air sensitivity of compounds **1–3**, the NMR samples were prepared under an argon atmosphere and sealed in airtight J. Young NMR tubes.

### UV–Vis Spectroscopy

UV–vis spectra were collected with an Agilent Cary 60 spectrometer at ambient temperature from 1100 to 200 nm for complexes **1**–**3**. Samples were prepared in an argon-filled glovebox and filtered into 1 cm quartz cuvettes outfitted with a Teflon screw cap. The spectra were baseline corrected from a sample of dry hexane. Each sample (**1**–**3**) was measured at concentrations of 20 and 100 μmol/L.

### Infrared Spectroscopy

IR spectra were recorded with an Agilent Cary 630 FTIR spectrometer on crushed crystalline solids under an inert nitrogen atmosphere.

### Magnetic Measurements

Magnetic susceptibility data were collected on a Quantum Design MPMS3 superconducting quantum interference device (SQUID) magnetometer. Magnetic samples of **2** and **3** were prepared by saturating and covering dried, crushed crystalline solids (**2**, 16.4 mg; **3**, 15.3 mg) with molten eicosane (**2**, 25.4 mg; **3**, 24.0 mg) at 45 °C to prevent crystallite torquing and to provide good thermal contact between the sample and the bath. The samples were sealed in an airtight container and transferred to the magnetometer. The core diamagnetism was estimated using Pascal’s constants.^43^

### Elemental Analysis

Elemental analysis was carried out with a PerkinElmer 2400 Series II CHNS/O analyzer. Crystalline compounds of all samples (∼1–3 mg) were weighed into tin sample holders that were folded multiple times under an argon atmosphere to ensure proper sealing from the surrounding atmosphere. The samples were then transferred to the instrument in an airtight container.

### DFT Calculations

Unrestricted density functional theory (DFT) calculations were carried out on a neutral toluene molecule and **1**, using ORCA version 5.0.4 ^44^ with a neutral charge and a spin multiplicity of 1. Crystal coordinates of **1** were optimized at the def2-SVP^45^ level of theory using the uTPSSh functional.^46–48^ Subsequent frequency calculations performed on optimized coordinates confirmed an energetic minimum structure through the absence of imaginary frequencies. Time-dependent DFT (TD-DFT) calculations were conducted using the uB3LYP functional^49^ at the def2-TZVP level on the optimized coordinates of **1** for 250 roots with a conductor-like polarizable continuum model (CPCM) hexane solvent model.^50^ Obtained transitions were shifted by 0.06 eV to better align with the experimental UV–vis data. Frontier molecular orbitals of neutral toluene were generated via a geometry optimization at the def2-TZVP level using the B3LYP functional. All calculations employed the resolution of identity (RI) approximation for the Coulomb integrals, while the exchange integrals were treated with the chain-of-spheres approximation (COSX). Grimme’s dispersion correction with a Becke–Johnson damping scheme (D3BJ)^51,52^ and a finer grid (defGRID3) were used for all calculations with auxiliary basis sets being generated by the AutoAux feature.^53^ Natural bond orbital (NBO) analysis was conducted using the NBO 7.0.8 program. NICS values were generated through the EPRNMR module of ORCA.^54^ Molecular orbital surfaces were visualized with VMD.^55,56^

## Results and Discussion

### Synthesis and Structural Characterization

The toluene-bridged rare earth metal complexes supported with a guanidinate ligand scaffold, [{(Me_3_Si)_2_NC­(N^i^Pr)_2_}_2_RE]_2_(μ-η^6^:η^6^-C_6_H_5_Me) (RE= Y (**1**), Dy (**2**), and Er (**3**)), were obtained through the reduction of the chloride-bridged RE complexes, [{(Me_3_Si)_2_NC­(N^i^Pr)_2_}_2_RE­(μ-Cl)]_2_, using the strong reductant potassium graphite (KC_8_) in diethyl ether in the presence of excess toluene (Figure 2A). Crystals of **1**–**3** suitable for single-crystal X-ray diffraction analysis were acquired from the slow evaporation of a pentane solution at room temperature in 33%, 46%, and 59% yields, respectively. While these crystals are extremely sensitive to air and moisture, they were stable at room temperature under an argon atmosphere for weeks. All three complexes are isostructural and crystallize in the *P*1̅ space group. Each structure features a dinuclear complex that resides on a crystallographic inversion center. The toluene moiety is disordered with the methyl group modeled over two sites such that the rare earth metals in each complex are equivalent by symmetry. The trivalent RE ions are bridged by a toluene dianion binding in an η^6^-fashion, and each metal center is ligated to two bidentate guanidinate ligands (Figure 2B and Figures S2A and S11A). Guanidinate ligands have proven to be effective in stabilizing transition metal complexes featuring bridging toluene moieties.^57,58^ However, these complexes contain only one ancillary guanidinate ligand on each metal ion, and the bridging toluene is neutral (Figure 1A),^57,58^ unlike **1**–**3** that feature two ancillary guanidinate ligands and a dianionic bridging toluene unit. While guanidinate ligands have been explored as ancillary ligands in stabilizing bridging toluene moieties in TM chemistry, **1**–**3** represent the first toluene-bridged RE complexes with supporting guanidinate scaffolds on the metal ions.

**Figure 2:**
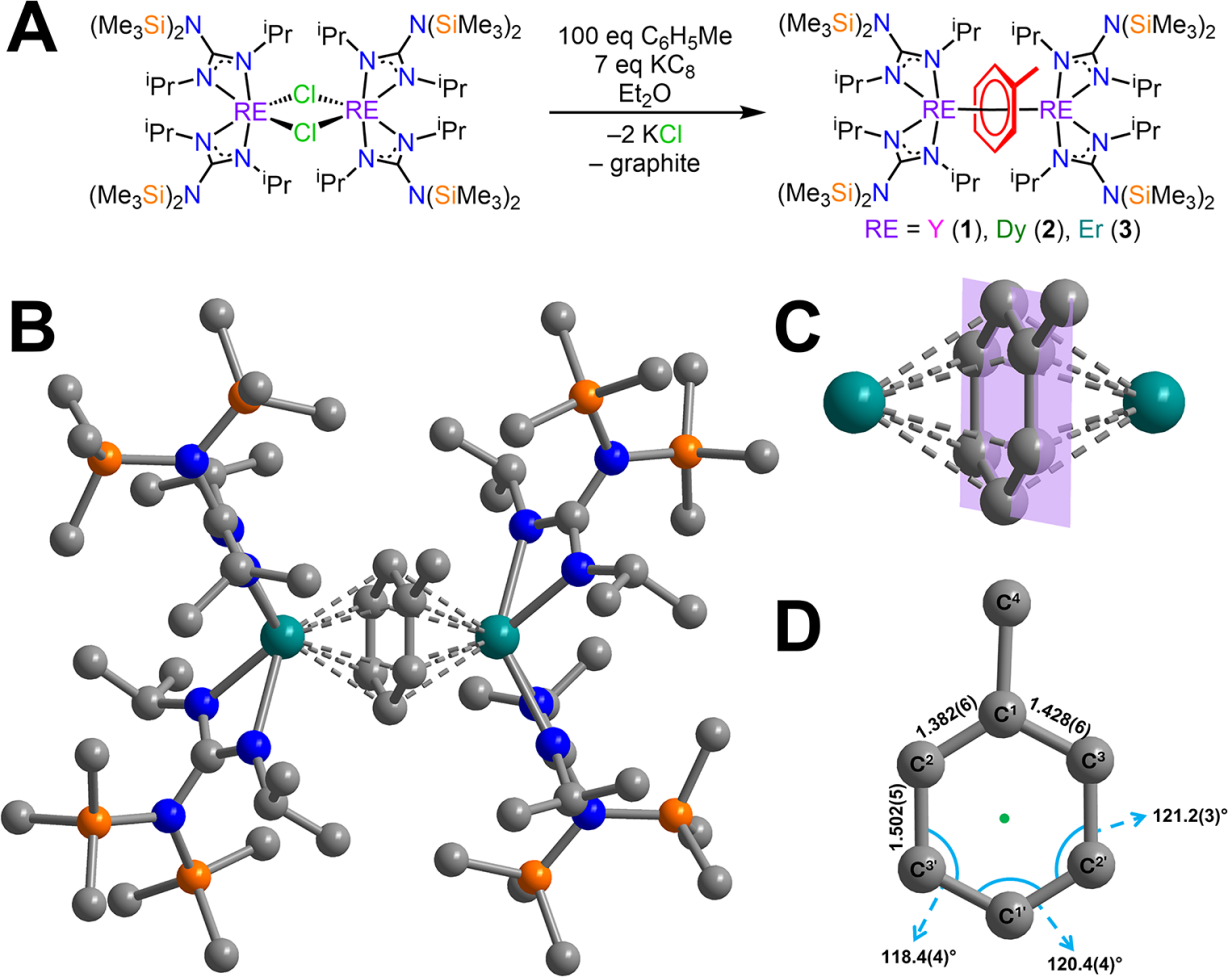
(A) Synthetic scheme for [{(Me_3_Si)_2_NC­(N^i^Pr)_2_}_2_RE]_2_(μ-η^6^:η^6^-C_6_H_5_Me), where RE = Y (**1**), Dy (**2**), and Er (**3**). (B) Structure of [{(Me_3_Si)_2_NC­(N^i^Pr)_2_}_2_Er]_2_(μ-η^6^:η^6^-C_6_H_5_Me) (**3**). Teal, orange, blue, and gray spheres represent Er, Si, N, and C atoms, respectively. H atoms have been omitted for clarity. Only one orientation of the disordered toluene dianion is shown for clarity. (C) Inverse-sandwich core of **3** enlarged with a reference plane through the phenyl moiety of toluene. Teal and gray spheres represent Er and C atoms, respectively. Ancillary guanidinate ligands and H atoms have been omitted for clarity. The mean plane is colored purple. (D) Dianionic toluene enlarged with distances (angstroms), angles (degrees), and corresponding atoms labeled. The pale green mark represents the inversion center.

Compounds **1**–**3** contain trivalent RE ions that are bridged by a dianionic toluene moiety. Here, the two bidentate guanidinate ligands bind to the metal ions in an asymmetric fashion with RE–N_guan_ distances of 2.343(2)–2.467(2) Å (Y), 2.344(4)–2.463(4) Å (Dy), and 2.315(3)–2.445(3) Å (Er). The range of distances for each compound is slightly elongated, albeit comparable to those of the parent chloride-bridged complexes, [{(Me_3_Si)_2_NC­(N^i^Pr)_2_}_2_RE­(μ-Cl)]_2_.^36–38^ Such a similarity suggests that the tripositive oxidation state of the RE ions in **1–3** is retained. The reduction of the rare earth metal center would coincide with an increase in the atomic radius of about 0.1 Å, which would be reflected in the RE–N_guan_ distances if the metal centers were reduced.^59^ Intriguingly, upon chemical reduction of the parent chloride-bridged complexes, the RE···RE distance increases from 4.278(1) Å (Y),^36^ 4.246(1) Å (Dy),^37^ and 4.217(1) Å (Er)^38^ to 4.575(1), 4.622(1), and 4.549(1) Å in **1**–**3**, respectively. Moving from the parent chloride-bridged complexes to the reduced arene bridge in the present work is accompanied by an increase in coordination number, which coincides with an increased RE···RE distance. Furthermore, these distances are significantly longer than that of the sum of the ionic radii of the two rare earth centers (when RE CN = 8, Σ­{RE^III^}_2_ = 2.038 Å (Y), 2.054 Å (Dy), and 2.008 Å (Er)) corresponding to differences of 2.537, 2.568, and 2.541 Å, for Y, Dy, and Er, respectively.^60^

The coordination of the μ-η^6^:η^6^-reduced toluene moiety in these complexes exhibits slight asymmetry, which is reflected in RE–C_arene_ distances of 2.690(3)–2.717(3), 2.695(6)–2.739(6), and 2.668(4)–2.706(3) Å, for **1**–**3**, respectively. An asymmetric coordination of a dianionic toluene bridge was observed in the Sm^II^ complex, [{Sm_2_(OSi­(O^t^Bu)_3_)_3_}_2_(μ-η^6^:η^6^-C_6_H_5_Me)], where the samarium center is divalent and exhibits Sm–C_arene_ distances of 2.523(7)–2.607(5) Å.^19^ Taken into account in this comparison is the inherently larger ionic radius of trivalent Sm, relative to those of trivalent Y, Dy, and Er ions in **1–3**, respectively, which should be amplified further through its divalent oxidation state.^60^ Accordingly, based on ionic radii, the RE–C_arene_ distances are expected to be shorter than in **1**–**3**, but in reality the distances are longer by approximately 0.11–0.17 Å. This is attributed to the lesser steric bulk imposed by siloxide ligands relative to guanidinate ligands (Figures S4, S13, and S21). In fact, guanidinates are sought after for their steric and electronic tunability, which can impact the stability, reactivity, and solubility of complexes, and have even been proposed as “steric cyclopentadienyl equivalents”.^61,62^ Furthermore, the RE–C_arene_ distances are shortened compared to those of neutral arene moieties, as is exemplified by the Dy–C_arene_ distances of 2.770(3)–2.941(3) Å in [(NHAr*)_2_Dy]­[BArF_24_], where Ar* = 2,6-(2,4,6-(^i^Pr)_3_C_6_H_2_)_2_C_6_H_3_,^15^ owing to the weaker electrostatic attraction of the neutral arene ring in NHAr* relative to the dianionic toluene unit in **1–3**. This is also reflected in the Dy–Cnt distance of 2.311 Å in **2** compared to 2.497 Å in [(NHAr*)_2_Dy]­[BArF_24_]. However, the RE–C_arene_ distances in **1** and **2** are markedly shorter by approximately 0.11–0.46 Å than those in [{(Me_3_Si)_2_NC­(N^i^Pr)_2_}_2_RE]­[(μ-η^6^-Ph)­(BPh_3_)] (where RE = 2.803(2)–3.190(2) Å for Y and 2.818(2)–3.197(2) Å for Dy).^11^ While both sets of complexes contain ancillary guanidinate ligands, [{(Me_3_Si)_2_NC­(N^i^Pr)_2_}_2_RE]­[(μ-η^6^-Ph)­(BPh_3_)] features a bulky and weakly coordinating [BPh_4_]^−^ anion, which allows for elongated RE–C_arene_ distances relative to those of **1** and **2**.

Insight into the charge of the bridging arene unit can be deconvoluted by examining the carbocyclic C–C interatomic distances, which in turn can reflect the spin multiplicity of the system. Here, the C–C distances are inequivalent, comprising two short (1.387(4) Å (**1**), 1.394(8) Å (**2**), and 1.382(6) Å (**3**)) and four long (1.433(4) and 1.505(4) Å (**1**), 1.411(8) and 1.490(7) Å (**2**), and 1.428(6) and 1.502(5) Å (**3**)) bonds, in line with a methylcyclohexadienediide structure (Figure 2D and Figures S2C and S11C). By contrast, neutral toluene features an average C–C bond distance of 1.387(10) Å.^63^ Notably, in **1–3**, the two shorter of the three distinct C–C distances reside adjacent to the *para* positions, where the methyl group is located, which is attributed to hyperconjugation from the weakly electron-donating methyl group. Furthermore, this interpretation is supported by the negative charges residing at the C^3^/C^3′^ positions in the reduced toluene unit, which further alludes to a cyclohexadienediide structure. The assignment of charges is further corroborated by natural population analysis (Figure S56).

This cyclohexadienediide-type structure was also observed for the reduced benzene dianion.^21,22,64,65^ Benzene dianions can be broadly classified into two distinct categories based on their electronic ground states, namely singlet or triplet configurations. In the case of the triplet state, the C–C distances are typically equivalent and the benzene moiety remains in the planar *D*_6*h*_ structure. Conversely, in the case of the singlet benzene dianion, the arene usually adopts a puckered structure.^64^ However, a planar singlet benzene dianion has also been realized by our group through the isolation of the RE complexes [{(Me_3_Si)_2_NC­(N^i^Pr)_2_}_2_RE]_2_(μ-η^6^:η^6^-C_6_H_5_)] (where RE = Y, Dy, and Er). Both electronic structure motifs of singlet and triplet ground states have been in fact observed in the realm of RE metal chemistry.^7,21,22,65,66^ Intriguingly, whether the arene ring will be planar or puckered based on the singlet or triplet ground state is less explored for inverse-sandwich complexes containing a bridging toluene compared to benzene. While **1**–**3** exhibit a methylcyclohexadienediide-type structure, complexes bearing divalent RE ions with a bridging reduced dianionic toluene moiety lack this motif.^19,28^ These complexes are innate to only slightly elongated C–C bonds (∼1.44–1.46 Å) that, while not perfectly equivalent, are far more uniform than those occurring in cyclohexadienediide-type systems.^20,22,65^

In contrast to these reduced benzene examples, the structural consequences of toluene reduction in RE chemistry are less explored. Importantly, complexes **1**–**3** feature a methylcyclohexadienediide-type dianionic toluene, which is planar, as confirmed by the C–C–C–C torsion angles that range from 1.0 to 1.9 deg (Figure 2C and Figures S2B and S11B). In fact, **1**–**3** constitute the first complexes composed of trivalent RE ions and a planar toluene dianion. This structural motif of a planar bridging toluene in **1**–**3** closely parallels the structural scenario observed in the RE metal complexes comprising a planar bridging benzene dianion, published by our group.^22^

### DFT Calculations

To further elucidate the metrical parameters obtained through crystallography and reveal the electronic structure of the discovered toluene-bridged complexes, DFT calculations were performed. Specifically, the diamagnetic nature of Y^III^ enables the facile elucidation of the electronic structure of **1** via computational methods. Density functional theory (DFT) calculations were carried out on the crystal coordinates of **1** to generate an energy-minimized structure (Table S4). The absence of imaginary frequencies in a frequency calculation performed on these coordinates confirmed the energetic minimum (Figure S32). These coordinates were used for all subsequent calculations.

The bond distances of the optimized structure can be compared with the respective distances of the crystallographically determined structure of **1** to gauge the validity of the DFT calculations. The optimized structure uncovers an Y···Y distance of 4.502 Å, whereas the Y–N distances for guanidinate coordination vary between 2.334 and 2.453 Å. The centroid of the bridging toluene ring is 2.257 Å from the yttrium­(III) centers relative to the corresponding distance of 2.288 Å seen in the crystal coordinates. Thus, the bond distances produced by geometry optimization are comparable to those found in the structure of **1** by single-crystal X-ray diffraction. These bond metric analyses support the validity of the implemented computational methodology and thereby the assignment of a singlet spin state.

The bridging toluene unit remains fully planar and retains its inequivalent C–C bond distances in the optimized structure: 1.386, 1.395, 1.423, 1.427, 1.499, and 1.507 Å. This result is congruent with the bond distances observed for the crystal coordinates of **1**, further confirming the presence of a methylcyclohexadienediide-type structure. Hence, the charge on this bridging toluene ring is assigned as −2, in accordance with the experimental data. In addition, as the experimental structural data suggest, the highest negative charges on the toluene bridge are concentrated at the C^3^/C^3′^ positions. The atomic charges allotted through natural population analysis support the assignment of a −2 charge for the arene ring of the toluene (Figure S56).

The analysis of the frontier molecular orbitals of **1** (Figure 3) afforded a highest occupied molecular orbital (HOMO) consisting of δ-bonding interactions between the yttrium­(III) centers and π* orbitals of the bridging toluene unit. The lowest unoccupied molecular orbital (LUMO), which lies energetically 1.65 eV higher than the HOMO, is composed of an orbital stemming from the Y^III^ centers and π orbitals of the toluene bridge. To further scrutinize the bonding situation of this complex, NBO analysis was carried out.

**Figure 3:**
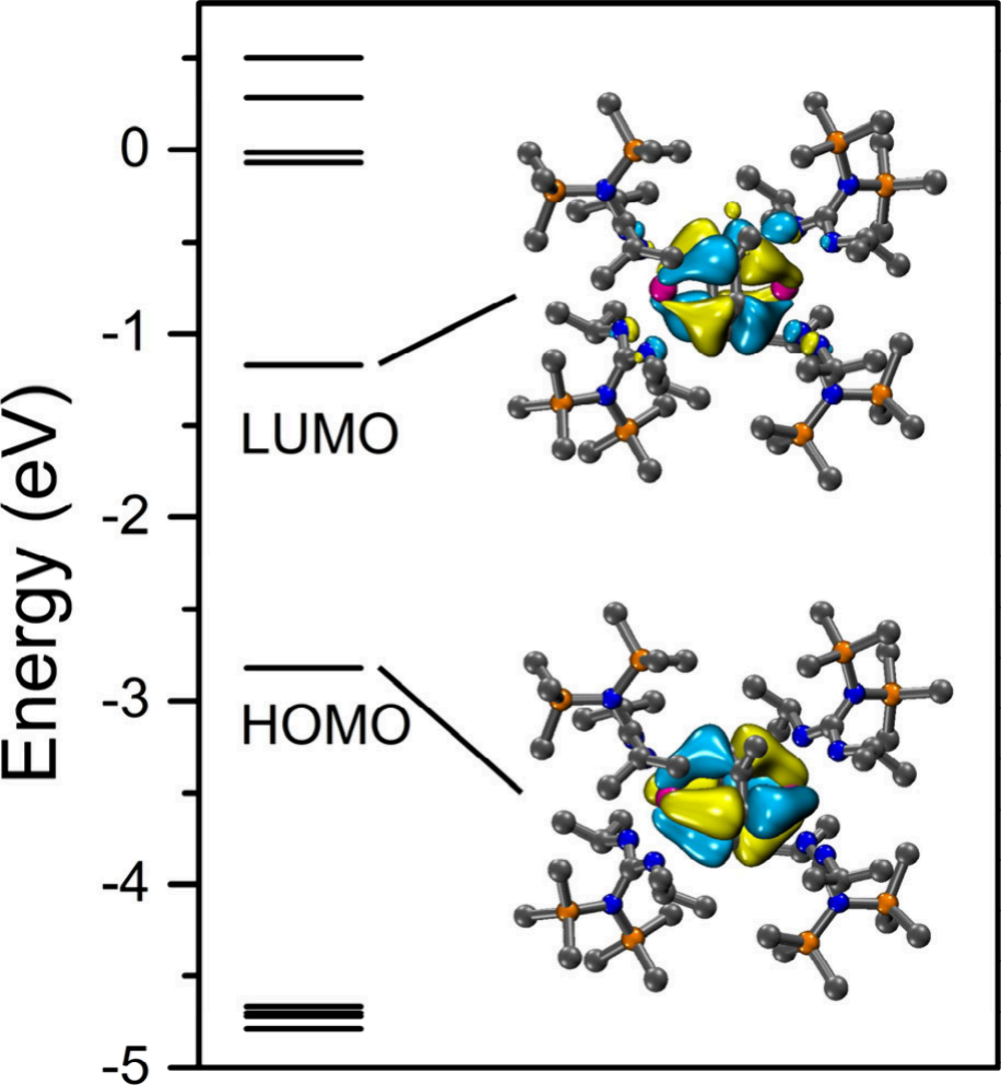
Frontier molecular orbitals of **1**. Pink, orange, blue, and gray spheres represent Y, Si, N, and C atoms, respectively. H atoms have been omitted for clarity. The isovalue for the depiction of surfaces is 0.03.

The strength of the bonding interaction can be deduced from second-order perturbation theory analysis in the NBO calculation. Based on these computational results, the strongest interactions between the bridging toluene unit and the Y^III^ centers originate from the lone pairs on the C atom p orbitals donating to the lone valence orbitals of the yttrium­(III) ions. The acceptor orbitals on the metal center are primarily of d character. Similarly, stronger donations are seen from the bonding orbitals between the sp^2^ carbons of toluene to the d lone valence orbitals of the yttrium­(III) centers. The stabilization energies for the aforementioned bonding interactions are in the range of ∼1–4 kcal/mol, indicating weaker orbital overlap and hence ionic interactions. These computationally determined bonding interactions between the toluene bridge and the yttrium­(III) centers are comparable to those ascertained for the arene–yttrium­(III) interactions for the analogous benzene-bridged complex, [{(Me_3_Si)_2_NC­(N^i^Pr)_2_}_2_RE]_2_(μ-η^6^:η^6^-C_6_H_5_Me), and the toluene-bridged amidinate complex, [{Tb­(κ^1^:η^6^-Piso)}_2_(μ-η^6^:η^6^-C_6_H_5_Me)]. This electrostatic attractive force between the two RE­(III) ions and the toluene dianion is the main reason why the toluene moiety adopts a planar configuration. The overall strength of the ionic interaction between the [{(Me_3_Si)_2_NC­(N^i^Pr)_2_}_2_Y]^+^ moiety and the (C_6_H_5_Me)^2–^ unit was determined to be 170 kcal/mol through a single-point DFT calculation.

The interactions of the yttrium ions with the guanidinate ligands are also mainly ionic in nature. The strongest bonding interactions (∼3–11 kcal/mol stabilization energy) arise from lone pair electrons on the sp^3^-hybridized orbitals of the nitrogen atoms to d orbitals of the Y^III^ centers. The higher stabilization energy indicates more orbital overlap owing to the more diffuse nature of these donor orbitals stemming from the higher p character.

The aromaticity of the bridging toluene unit was probed through calculation of the nucleus-independent chemical shift (NICS)^54^ values for **1** (Figure 4). This calculation was conducted by placing dummy atoms at 0.1 Å intervals along the axis perpendicular to the arene ring. The calculated NICS values exhibit a trend with a NICS(0) value of 37.69 ppm and a gradual decrease to a NICS(1) value of about 35 ppm. These significantly positive NICS values are characteristic of a paratropic ring current on the arene ring. Such a paratropic ring current in the singlet state suggests antiaromaticity of the arene bridge, which is consistent with the expected 4n π-electron configuration for a toluene dianion.

**Figure 4:**
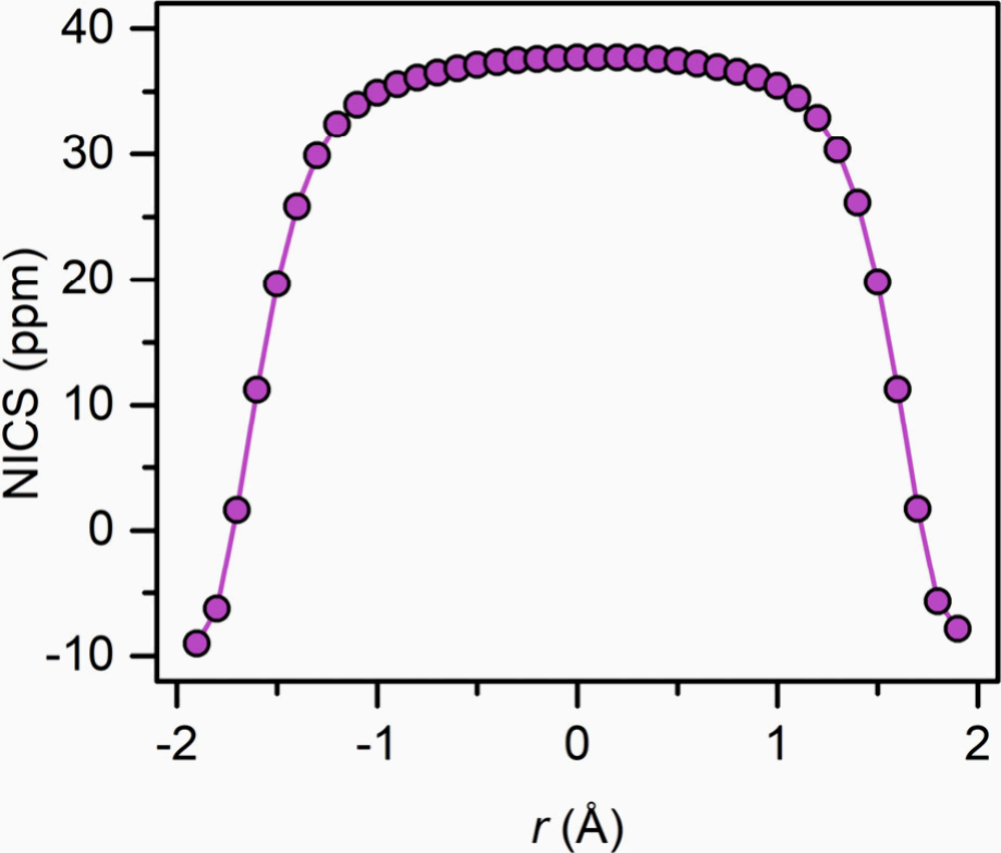
NICS values generated by the isotropic chemical shielding values for dummy atoms placed along an axis perpendicular to the arene ring of toluene in **1** at 0.1 Å intervals.

A comparison of the NICS values to those of the benzene-bridged Y complex, [{(Me_3_Si)_2_NC­(N^i^Pr)_2_}_2_Y]_2_(μ-η^6^:η^6^-C_6_H_5_),^22^ reveals that in both cases the data follow a trend with a maximum positive NICS value at the centroid with a gradual decrease up to ∼1.2 Å, after which a pronounced decrease occurs. This progression of the NICS values points at antiaromaticity in **1**, which is also observerd for the dianionic benzene bridge. Accordingly, the NICS values for both cases are consistent and confirm antiaromaticity, which in fact is expected for both of these six-membered arene systems upon chemical double reduction of the parent neutral arenes.

### Spectroscopy

The collected Fourier-transformed infrared (FTIR) spectra for **1**–**3** exhibit superimposable features across the measured wavenumber region, from 3100 to 650 cm^–1^ (Figures S32–S34). Vibrations originating from methyl C–H stretches are observed in the ∼2900 cm^–1^ region as strong absorptions in all three compounds, which is ascribed to the multiple methyl groups present on the guanidinate scaffold. The strong absorption peaks at 1450 and 1640 cm^–1^ may be attributable to the C–C stretches intrinsic to bridging toluene. The intense peaks visible at 747 and 701 cm^–1^ are ascribed to toluene C–H vibrations.

The solution state structure of **1** was studied through ^1^H NMR spectroscopy where data collection proceeded in deuterated toluene (Figure S28). Proton resonances diagnostic of the trimethylsilyl groups of the guanidinate scaffold are found at 0.15 and 0.54 ppm. The isopropyl protons of the guanidinate ligands constitute the doublets located at 1.04 and 1.61 ppm. The doubly negative charge present on the toluene bridge results in a significant shielding effect that is reflected by the upfield shift observed for the relevant proton resonances. The methyl protons of toluene are assigned to a singlet observed at 3.14 ppm. The protons at the *ortho* positions can be ascribed to the resonance occurring at 1.84 ppm, and the protons at the *meta* positions cause the resonance at 2.49 ppm. The proton at the *para* position integrates to a resonance peak seen at 3.98 ppm. These assignments indicate that in the solution phase, the inequivalence of protons is derived from the presence of the methyl group rather than from a cyclohexadienediide-type arene ring. This inference agrees with the ^1^H NMR data reported for the analogous benzene-bridged complex that show the six protons on the benzene ring to be chemically equivalent.

UV–vis spectra were collected from 200 to 1100 nm for **1**–**3** in hexane under inert conditions. All three complexes exhibit similar absorption features that span from the UV region to around 600 nm in the visible region (Figure 5A). This observation is congruent with the orange and red colors exhibited by solutions of **1**–**3**. The similarity of the electronic absorptions across the three compounds indicates that the observable electronic excitations are mainly ligand-based charge transfer events.

**Figure 5:**
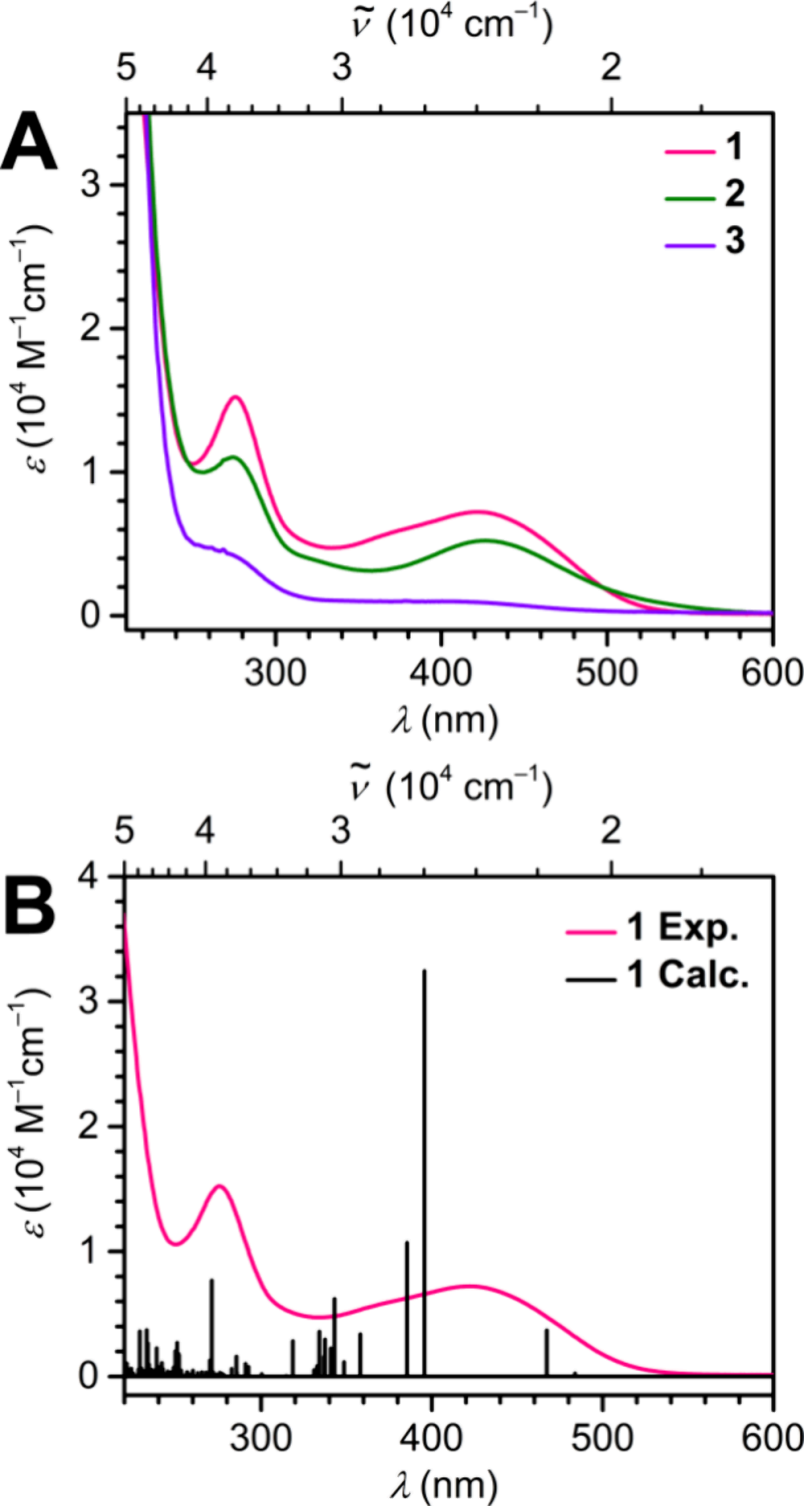
(A) UV–vis spectra of **1** (pink trace), **2** (dark green trace), and **3** (purple trace) collected in hexane with an analyte concentration of 20 μM. (B) Experimental UV–vis spectrum of **1** and calculated TD-DFT transitions of **1** (black vertical lines).

The strongest absorption observed for all compounds falls in the UV region closer to 220 nm (4.94 × 10^4^ cm^–1^). The second most prominent feature is seen as a narrow absorption band at around 275 nm (3.87 × 10^4^ cm^–1^). A broader absorption band spanning ∼150 nm is observed centered at 427 nm (2.38 × 10^4^ cm^–1^). A less obvious absorption feature can be seen buried beneath the broad feature at around 320 nm (3.12 × 10^4^ cm^–1^). The absence of absorption features at higher wavelengths ascertains the lack of transitions corresponding to f–f excitations, agreeing with the trivalent oxidation state of the RE ions.^67^ The absorption features observed for compounds **1**–**3** are comparable to those seen for the analogous benzene-bridged complexes, [{(Me_3_Si)_2_NC­(N^i^Pr)_2_}_2_RE]_2_(μ-η^6^: η^6^-C_6_H_6_) (RE = Y, Dy, and Er), further indicating that the primary electronic transitions involve the guanidinate scaffold and the arene ring.^22^ The individual electronic excitations contributing to UV–vis absorption features are further discussed below.

Time-dependent DFT (TD-DFT) calculations were performed on the optimized coordinates of **1** to predict the electronic excitations giving rise to UV–vis absorption features (Figure 5B). The strongest predicted transition is at 373 nm (2.68 × 10^4^ cm^–1^) and corresponds to an excitation originating from HOMO to HOMO+4, primarily an Y-based orbital with d character. The second most prominent transition is at 364 nm (2.74 × 10^4^ cm^–1^) and arises due to an excitation from HOMO to HOMO+5, which can be described as an orbital with significant contributions from the yttrium centers as well as the guanidinate scaffold. The strongest absorption in the visible region is due to an excitation centered around 437 nm (2.29 × 10^4^ cm^–1^). The occupied and virtual orbitals giving rise to this transition are HOMO and HOMO+12 consisting of contributions from the bridiging toluene unit and the yttrium centers, respectively. Depictions of the orbitals involved in the major TD-DFT transitions and relevant details are described in Table S3.

### Magnetic Characterization

To shed light on the magnetic properties of **2** and **3**, direct current (dc) molar magnetic susceptibility (χ_M_ *T*) data were collected on restrained polycrystalline samples between 2 and 300 K under 0.1 and 1.0 T applied dc fields (Figure 6 and Figures S39–S45). At 0.1 T, the room-temperature χ_M_ *T* values of 28.06 and 23.36 cm^3^ K mol^–1^ are in agreement with the expected values for two noninteracting Dy^III^ and Er^III^ ions, respectively (for Dy^III^, 4f^9^, ^6^H_15/2_, *S* = 5/2, *L* = 5, *J* = 15/2, *g* = 4/3, and χ_M_ *T*_calc_ = 28.33 cm^3^ K mol^–1^; for Er^III^, 4f^11^, ^4^I_15/2_, *S* = 3/2, *L* = 6, *J* = 15/2, *g* = 6/5, and χ_M_ *T*_calc_ = 22.95 cm^3^ K mol^–1^). As the temperature is decreased, a gradual downturn is monitored for **2**, reaching a value of 18.62 cm^3^ K mol^–1^ at 10 K, before precipitously decreasing to 9.95 cm^3^ K mol^–1^ at 2 K. At 1.0 T, a similar trend is observed, where the molar magnetic susceptibility and temperature product steadily decrease until 2 K. This gradual decline in the χ_M_ *T* value is attributed to the depopulation of crystal field states and alludes to the absence of magnetic exchange coupling in **2**. By contrast, the χ_M_ *T* value of **3** increases gradually upon cooling before rapidly rising to a maximum of 33.00 cm^3^ K mol^–1^ under a 0.1 T applied dc field. Subsequently, the χ_M_ *T* value decreases to a value of 29.90 cm^3^ K mol^–1^ at 2 K. Under a 1.0 T applied dc field, the χ_M_ *T* value remains constant until 50 K before gently increasing to 26.43 cm^3^ K mol^–1^ at 13 K and subsequently decreasing to 9.52 cm^3^ K mol^–1^ at 2 K. The rise in χ_M_ *T* upon lowering the temperature is indicative of the presence of weak magnetic exchange coupling, which is markedly diminished upon the application of the stronger 1.0 T magnetic field. The absence of an uptick in χ_M_ *T* for **2** suggests that the magnetic coupling in **3** orginates from intramolecular dipolar coupling, which likely arises from the short intramolecular Er^III^···Er^III^ distance in **3** of 4.549(1) Å.^68^

**Figure 6:**
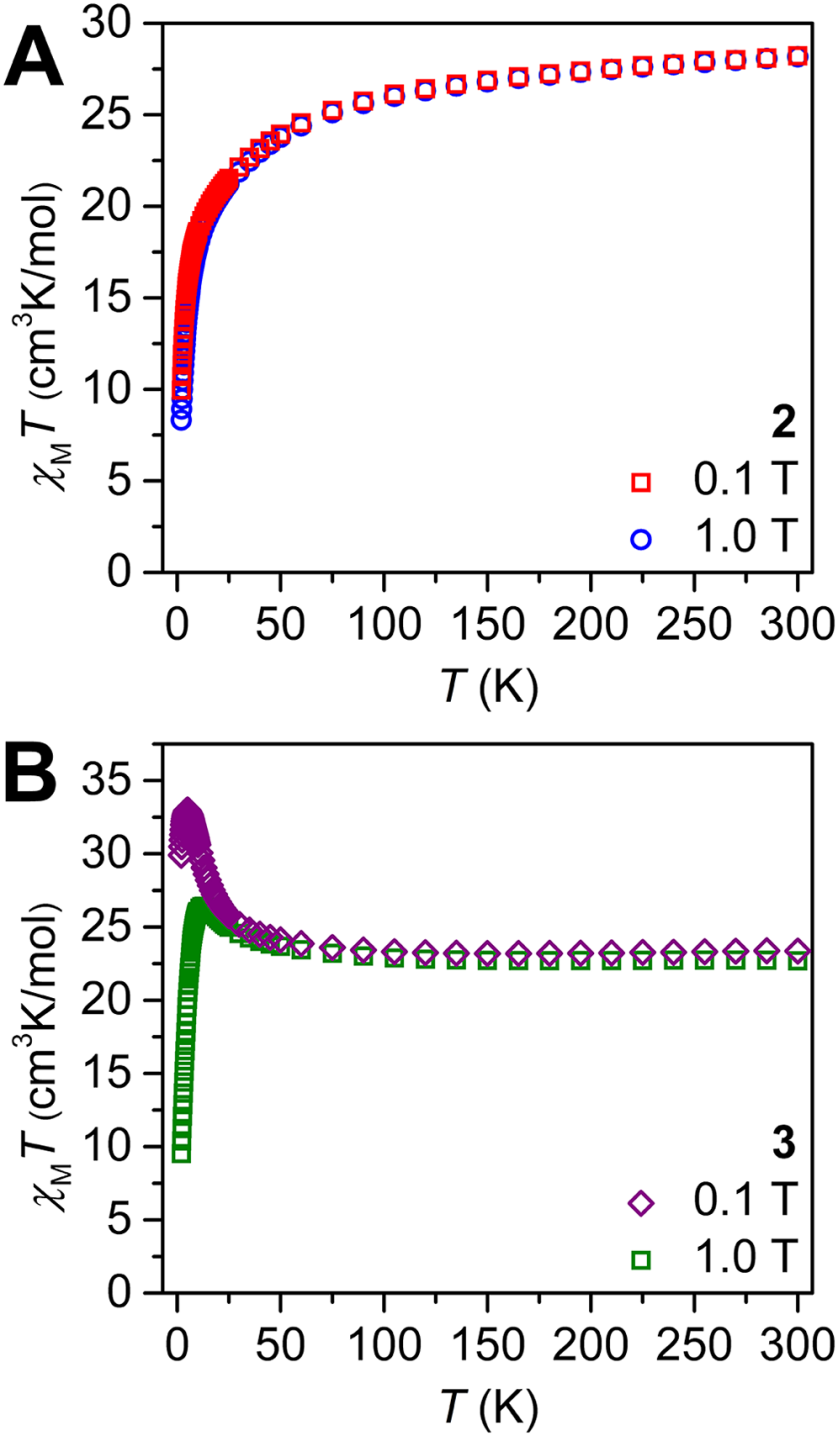
Variable-temperature dc magnetic susceptibility data for restrained polycrystalline samples of (A) [{(Me_3_Si)_2_NC­(N^i^Pr)_2_}_2_Dy]_2_(μ-η^6^:η^6^-C_6_H_5_Me) (**2**) and (B) [{(Me_3_Si)_2_NC­(N^i^Pr)_2_}_2_Er]_2_(μ-η^6^:η^6^-C_6_H_5_Me) (**3**), collected under 0.1 and 1.0 T applied dc fields.

The field-dependent magnetization data (*M* vs *H*) for **2** and **3** were recorded from 2 to 10 K (Figure 7 and Figures S46–S48) and revealed nonsuperimposable reduced magnetization curves, consistent with the presence of pronounced magnetic anisotropy. At low temperatures, the magnetization values for **2** and **3** slowly rise with increasing field strength before saturating at 11.34 and 10.30 μ_B_, respectively, which is consistent with the magnetization values expected for two Dy^III^ and Er^III^ ions.^69–71^

**Figure 7:**
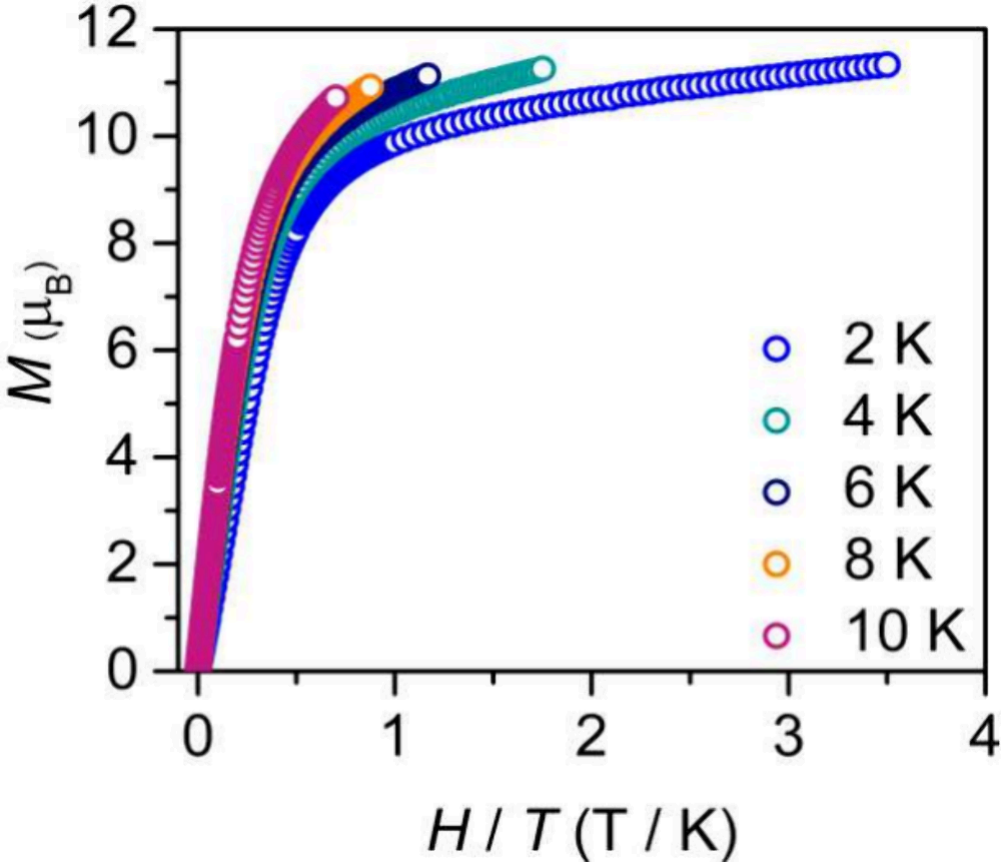
Variable-temperature field-dependent reduced magnetization curves recorded for [{(Me_3_Si)_2_NC­(N^i^Pr)_2_}_2_Dy]_2_(μ-η^6^:η^6^-C_6_H_5_Me) (**2**). Measurements were carried out from 0 to 7 T at 2, 4, 6, 8, and 10 K.

The dynamic magnetic properties of **2** and **3** were probed via variable-temperature, variable-frequency alternating current (ac) magnetic susceptibility measurements. For **2** and **3**, no out-of-phase (χ_M_″) signals were observed under zero applied dc field. This suggests that rapid magnetic relaxation pathways such as quantum tunneling of the magnetization (QTM) are operative in the samples. Rapid relaxation pathways may be suppressed through the application of an external magnetic field.^72^ Applied dc fields ranging from 500 to 2000 Oe for **2** and from 500 to 3000 Oe for **3** strongly influenced the shape of the χ_M_″ frequency scan for both complexes. In each case, broad χ_M_″ signals were observed at high frequencies (Figures S49 and S52).

The optimal dc fields for **2** and **3** were determined to be 1500 and 500 Oe, respectively. Under the application of a 1500 Oe dc magnetic field, a χ_M_″ maximum was observed for the Dy congener at high frequencies from 1.8 to 2.0 K (Figures S50 and S51). For **3**, under a 500 Oe applied dc field, at frequencies between 0.1 and 1000 Hz, a χ_M_″ maximum was observed at 573 Hz at 1.8 K, which moved to higher frequencies as the temperature was increased to 2 K, suggestive of single-molecule magnet behavior (Figure 8 and Figure S53). However, the few accessible temperatures prevented unambiguous determination of magnetic relaxation times. To further elucidate the magnetic properties of **2** and **3**, field-dependent magnetization (*M* vs *H*) data were collected between 7 and – 7 T at 1.8 K. Here, each complex exhibited a superimposable curve, which alludes to the presence of rapid magnetic relaxation mechanisms such as QTM (Figures S54 and S55). The lack of remanent magnetization when traversing zero field is in accordance with the fast magnetic relaxation observed through ac magnetic susceptibility measurements under zero applied dc field.

**Figure 8:**
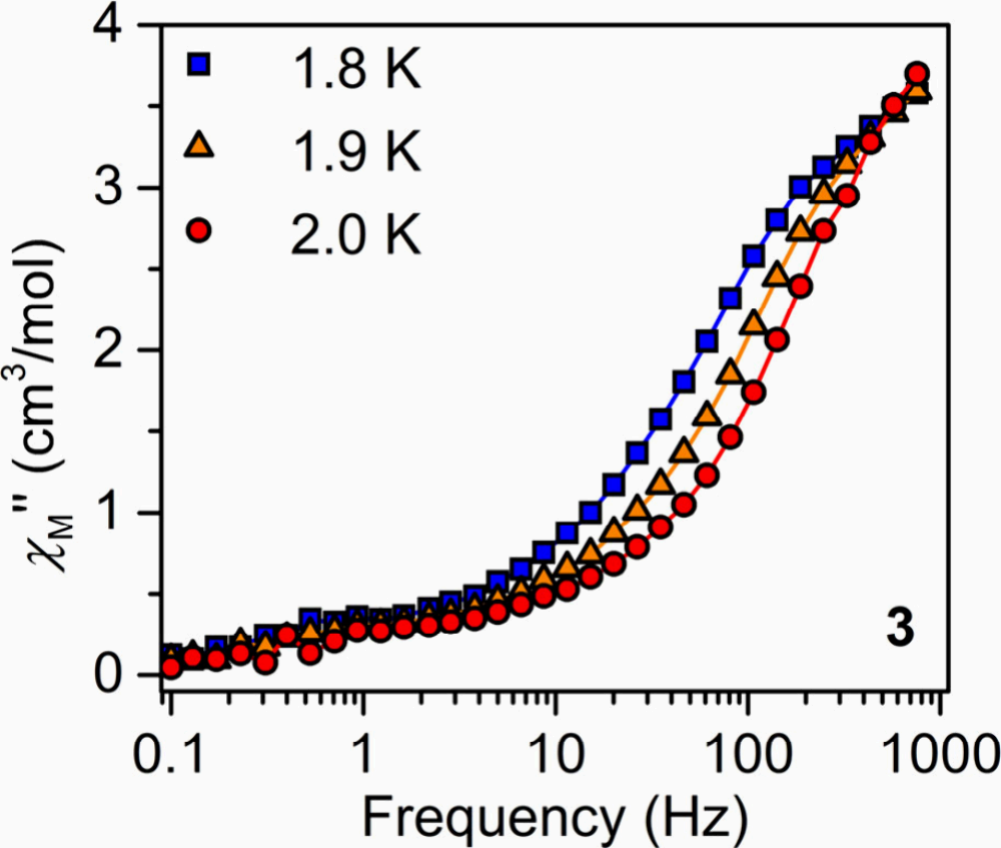
Out-of-phase (χ_M_″) components of the ac magnetic susceptibility for [{(Me_3_Si)_2_NC­(N^i^Pr)_2_}_2_Er]_2_(μ-η^6^:η^6^-C_6_H_5_Me) (**3**) at 1.8 K under a 500 Oe applied dc field. Solid lines represent guides for the eye.

While the electronic structure of lanthanide ions such as Dy^III^ and Er^III^ are predominantly governed by spin–orbit coupling, perturbations arising from crystal field interactions remain non-negligible and can strongly influence the observed magnetic properties.^73^ More specifically, fine-tuning the crystal field of Ln^III^ ions can give rise to a large separation between the ground and excited state doublets. This can usher in the observation of slow magnetic relaxation in single molecules, akin to bulk-like magnet behavior. Taking this into account, the magnetic behavior of topologically related benzene-bridged lanthanide complexes [{(Me_3_Si)_2_NC­(N^i^Pr)_2_}_2_Ln]_2_(μ-η^6^:η^6^-C_6_H_6_) (where Ln = Dy and Er)^22^ can be compared to that of the title compounds **2** and **3**. These two sets of molecules differ essentially in the activated dianionic arene bridge, where this seemingly structurally small difference has a profound impact on the dynamic magnetic properties. Intriguingly, the benzene-bridged Er complex exhibits an energy barrier to spin reversal of *U*_eff_ = 14.0(3) cm^–1^, which corresponds to a slower magnetic relaxation than observed for **3**. Evidently, the nature of the arene bridge has an effect on relaxation. Within **2** and **3**, the bridging toluene dianion functions as a stronger π donor with respect to benzene, owing to the presence of an electron-donating methyl group. Here, the increase in the extent of π donation from the toluene moiety may engender greater transverse anisotropy and sublevel mixing, leading to faster rates of QTM.^72^ Thus, this slight perturbation in electronic effects coupled with the decrease in symmetry from benzene to toluene may be responsible for the observed magnetic behavior of **2** and **3**.

## Conclusion

The synthesis and characterization of inverse-sandwich complexes bearing trivalent rare earth metal ions that are bridged by a planar toluene dianion are reported. Notably, these represent the first inverse-sandwich compounds composed of a dianionic toluene and metal ions that are supported by ancillary guanidinate scaffolds. The title complexes, [{(Me_3_Si)_2_NC­(N^i^Pr)_2_}_2_RE]_2_(μ-η^6^:η^6^-C_6_H_5_Me) (RE = Y (**1**), Dy (**2**), and Er (**3**)), were isolated and crystallographically characterized, unveiling a methylcyclohexadienediide-type toluene structure, supporting the assignment of a −2 charge to the bridging toluene. This inference is further reinforced via DFT calculations performed on the diamagnetic yttrium congener, demonstrating the metal–arene ionic bonding interactions using NBO analyses. ^1^H NMR spectroscopic data of **1** and UV–vis spectra for **1**–**3** further corroborate these findings. In addition, weak magnetic exchange coupling between the Ln^III^ ions was uncovered for **2** and **3** through variable-temperature dc magnetic susceptibility measurements. In the case of **2** and **3**, slow magnetic relaxation was observed under 1500 and 500 Oe applied dc fields between 1.8 and 2 K on the time scale of ac magnetic susceptibility measurements.

Collectively, these results demonstrate that highly reactive, activated arene systems can be stabilized through the employment of RE^III^ ions. This in turn enables the utilization of such negatively charged arene systems as effective bridging ligands, facilitating the design of multimetallic rare earth complexes. Such functionality may be exploited in the judicious design of molecules with electronic structures of interest toward spintronic applications. Future studies will explore the reactivity of these dianionic arene-bridged complexes to assess their bond activation and electron transfer ability. Specifically, these compounds may have similar reactivity relative to divalent lanthanides, enabled through the dianonic toluene ready to be extruded in reactions. Overall, this work establishes a platform for exploring potential applications of guanidinate-supported rare earth inverse-sandwich complexes bearing reduced arene systems.

## Supplementary Material



## Notes

Crystallographic refinement parameters, NMR, IR, and UV–vis spectra, magnetic data, computational data, and coordinates of optimized structures (PDF)

## References

[1] Weiss E., Fischer E. O. (1956). Über Aromatenkomplexe von Metallen. II. Zur
Kristallstruktur und Molekelgestalt des Di-benzol-chrom(0). Z. Anorg. Allg. Chem..

[2] Tricoire M., Sroka W., Rajeshkumar T., Scopelliti R., Sienkiewicz A., Maron L., Mazzanti M. (2025). Multielectron
Redox
Chemistry of Ytterbium Complexes Reaching the + 1 and Zero Formal
Oxidation States. J. Am. Chem. Soc..

[3] Brennan J. G., Cloke F. G. N., Sameh A. A., Zalkin A. (1987). Synthesis of Bis­(η-1,3,5-Tri-t-Butylbenzene)
Sandwich Complexes of Yttrium(0) and Gadolinium(0); the X-Ray Crystal
Structure of the First Authentic Lanthanide(0) Complex, [Gd­(η-Bu^t^
_3_C_6_H_3_)_2_]. J. Chem. Soc., Chem. Commun..

[4] Jena R., Benner F., Delano IV F., Holmes D., McCracken J., Demir S., Odom A. L. (2023). A Rare
Isocyanide Derived from an
Unprecedented Neutral Yttrium­(II) Bis­(Amide) Complex. Chem. Sci..

[5] Benner F., Pugliese E. R., Castellanos E., Deshapriya S., Demir S. (2025). Slow Magnetic Relaxation in a Rare,
Neutral, Formally Divalent Terbium
Bis­(Amide) Complex. Inorg. Chem..

[6] Arnold P. L., Petrukhina M. A., Bochenkov V. E., Shabatina T. I., Zagorskii V. V., Sergeev G. B., Cloke F. G. N. (2003). Arene Complexation
of Sm, Eu, Tm and Yb Atoms: A Variable Temperature Spectroscopic Investigation. J. Organomet. Chem..

[7] Wang Y., Sun R., Liang J., Zhang Y., Tan B., Deng C., Wang Y.-H., Wang B.-W., Gao S., Huang W. (2025). Synthesis
and Stabilization of a Benzene Dianion with a Triplet Ground State
and Baird Aromaticity. J. Am. Chem. Soc..

[8] Mazzanti M. (2018). The Secret
Is in the Ring. Nature Chem..

[9] Halter D. P., Heinemann F. W., Maron L., Meyer K. (2018). The Role of Uranium-Arene
Bonding in H_2_O Reduction Catalysis. Nature Chem..

[10] Richardson G. M., Rajeshkumar T., Burke F. M., Cameron S. A., Nicholls B. D., Harvey J. E., Keyzers R. A., Butler T., Granville S., Liu L., Langley J., Lim L. F., Cox N., Chilton N. F., Hicks J., Davis N. J. L. K., Maron L., Anker M. D. (2025). Four-Electron
Reduction of Benzene by a Samarium­(II)-Alkyl without the Addition
of External Reducing Agents. Nat. Chem..

[11] Delano
IV F., Demir S. (2023). Guanidinate Rare-Earth Tetraphenylborate Complexes
and Their Prospects in Single-Molecule Magnetism. Cryst. Growth Des..

[12] Demir S., Lorenz S. E., Fang M., Furche F., Meyer G., Ziller J. W., Evans W. J. (2010). Synthesis,
Structure, and Density
Functional Theory Analysis of a Scandium Dinitrogen Complex, [(C_5_Me_4_H)_2_Sc]_2_(μ-η^2^:η^2^-N_2_). J. Am. Chem. Soc..

[13] Cloke F. G. N., Khan K., Perutz R. N. (1991). η-Arene
Complexes of Scandium(0)
and Scandium­(II). J. Chem. Soc., Chem. Commun..

[14] Jena R., Benner F., Staples R. J., Demir S., Odom A. L. (2025). A Neutral
Dy­(II) Bis­(Amide): Synthesis, Magnetism, and a P_4_
^2–^ Complex. Inorg. Chem..

[15] Benner F., Jena R., Odom A. L., Demir S. (2025). Magnetic Hysteresis
in a Dysprosium Bis­(Amide) Complex. J. Am. Chem.
Soc..

[16] Demir S., Gonzalez M. I., Darago L. E., Evans W. J., Long J. R. (2017). Giant Coercivity
and High Magnetic Blocking Temperatures for N_2_
^3‑^ Radical-Bridged Dilanthanide Complexes upon Ligand Dissociation. Nat. Commun..

[17] Evans W. J., Davis B. L., Champagne T. M., Ziller J. W. (2006). C-H Bond Activation
through Steric Crowding of Normally Inert Ligands in the Sterically
Crowded Gadolinium and Yttrium (C_5_Me_5_)_3_M Complexes. Proc. Natl. Acad. Sci. U.S.A..

[18] Pugliese E. R., Benner F., Demir S. (2023). From an Isolable Bismolyl Anion to
an Yttrium-Bismolyl Complex with μ-Bridging Bismuth­(I) Centers
and Polar Covalent Y-Bi Bonds. Chem. - Eur.
J..

[19] Kelly R. P., Toniolo D., Tirani F. F., Maron L., Mazzanti M. (2018). A Tetranuclear
Samarium­(II) Inverse Sandwich from Direct Reduction of Toluene by
a Samarium­(II) Siloxide. Chem. Commun..

[20] Cassani M.
C., Duncalf D. J., Lappert M. F. (1998). The First Example of a Crystalline
Subvalent Organolanthanum Complex: [K([18]­Crown-6)-(η^2^-C_6_H_6_)_2_]­[(LaCp^tt^
_2_)_2_(μ-η^6^:η^6^-C_6_H_6_)]•2C_6_H_6_ (Cp^tt^ = η^5^-C_5_H_3_Bu^t^
_2_-1,3). J. Am. Chem. Soc..

[21] Cassani M.
C., Gun’ko Y. K., Hitchcock P. B., Lappert M. F. (1996). The First Metal
Complexes Containing the 1,4-Cyclohexa-2,5-Dienyl Ligand (Benzene
1,4-Dianion); Synthesis and Structures of [K­(18-Crown-6)]­[Ln­{η^5^-C_5_H_3_(SiMe_3_)_2_-1,3}_2_(C_6_H_6_)]­(Ln = La, Ce). Chem. Commun..

[22] Delano
IV F., Demir S. (2025). Stabilization of the Compressed Planar Benzene Dianion
in Inverse-Sandwich Rare Earth Metal Complexes. Angew. Chem., Int. Ed..

[23] McClain K. R., Vincent A. H., Rajabi A., Ngo D. X., Meihaus K. R., Furche F., Harvey B. G., Long J. R. (2024). Linear Inverse Sandwich
Complexes of Tetraanionic Benzene Stabilized by Covalent δ-Bonding
with Late Lanthanides. J. Am. Chem. Soc..

[24] Liu M., Chen Y.-C., Mondal A., Wang H., Tong M.-L., Layfield R. A., Guo F.-S. (2025). η^6^-Benzene Tetra-Anion
Complexes of Early and Late Rare-Earth Metals. J. Am. Chem. Soc..

[25] Jin P.-B., Luo Q.-C., Gransbury G. K., Winpenny R. E. P., Mills D. P., Zheng Y.-Z. (2025). Rare Earth Benzene
Tetraanion-Bridged Amidinate Complexes. Chem.
Sci..

[26] Gentner T. X., Rösch B., Ballmann G., Langer J., Elsen H., Harder S. (2019). Low Valent Magnesium Chemistry with a Super Bulky β-Diketiminate
Ligand. Angew. Chem., Int. Ed..

[27] Mortensen J., Heinze J. (1984). The Electrochemical
Reduction of Benzene—First
Direct Determination of the Reduction Potential. Angew. Chem., Int. Ed..

[28] Kelly R. P., Maron L., Scopelliti R., Mazzanti M. (2017). Reduction of a Cerium­(III)
Siloxide Complex To Afford a Quadruple-Decker Arene-Bridged Cerium­(II)
Sandwich. Angew. Chem., Int. Ed..

[29] Gun’ko Y. K., Hitchcock P. B., Lappert M. F. (2000). Nonclassical Organolanthanoid Metal
Chemistry: [K([18]-Crown-6)­(η^2^-PhMe)_2_]­X
(X = [(LnCp^t^
_3_)_2_(μ-H)], [(LnCp‘‘_2_)_2_(μ-η^6^:η^6^-PhMe)]) from [LnCp^x^
_3_], K, and [18]-Crown-6
in Toluene (Ln = La, Ce; Cp^t^ = η^5^-C_5_H_4_SiMe_2_Bu^t^; Cp‘‘
= η^5^-C_5_H_3_(SiMe_3_)_2_-1,3). Organometallics.

[30] Zhang L., Jiang Z., Zhang C., Cheng K., Li S., Gao Y., Wang X., Chu J. (2025). Room Temperature Ring Opening of
Benzene by Four-Electron Reduction and Carbonylation. J. Am. Chem. Soc..

[31] Crawford V. A. (1949). Hyperconjugation. Q. Rev. Chem. Soc..

[32] Deshapriya S., Delano IV F., Demir S. (2024). Magnetic and Spectroscopic Properties
of Chloride-Bridged Guanidinate Dilanthanide Complexes. Polyhedron.

[33] Delano
IV F., Deshapriya S., Demir S. (2024). Guanidinate Yttrium Complexes Containing
Bipyridyl and Bis­(Benzimidazolyl) Radicals. Inorg. Chem..

[34] Luo Y., Yao Y., Shen Q., Yu K., Weng L. (2003). Synthesis
and Characterization
of Lanthanide­(III) Bis­(Guanidinate) Derivatives and the Catalytic
Activity of Methyllanthanide Bis­(Guanidinate) Complexes for the Polymerization
of ϵ-Caprolactone and Methyl Methacrylate. Eur. J. Inorg. Chem..

[35] Bergbreiter D. E., Killough J. M. (1978). Reactions of Potassium-Graphite. J. Am. Chem. Soc..

[36] Lu Z., Yap G. P. A., Richeson D. S. (2001). Tetrasubstituted
Guanidinate Anions
as Supporting Ligands in Organoyttrium Chemistry. Organometallics.

[37] Jin P., Yu K., Zhai Y., Luo Q., Wang Y., Zhang X., Lv Y., Zheng Y. (2021). Chelating Guanidinates for Dysprosium­(III) Single-Molecule
Magnets. Chin. J. Chem..

[38] Delano
IV F., Demir S. (2024). Unprecedented Pyrazine-Bridged Guanidinate Rare Earth
Complexes Through a Bridge Splitting Reaction Path. Eur. J. Inorg. Chem..

[39] Rigaku Oxford Diffraction . CrysAlisPro Software System, ver. 1.171.44.103a; 2025.

[40] Sheldrick G. M. (2015). *SHELXT* - Integrated Space-Group and Crystal-Structure Determination. Acta Crystallogr. A Found Adv..

[41] Sheldrick G. M. (2015). Crystal
Structure Refinement with *SHELXL*. Acta Crystallogr. C Struct Chem..

[42] Dolomanov O. V., Bourhis L. J., Gildea R. J., Howard J. A. K., Puschmann H. (2009). OLEX2: A Complete
Structure Solution, Refinement and Analysis Program. J. Appl. Crystallogr..

[43] Bain G. A., Berry J. F. (2008). Diamagnetic Corrections
and Pascal’s Constants. J. Chem. Educ..

[44] Neese F. (2012). The ORCA Program
System. Wiley Interdiscip. Rev.: Comput. Mol.
Sci..

[45] Weigend F., Ahlrichs R. (2005). Balanced Basis Sets of Split Valence, Triple Zeta Valence
and Quadruple Zeta Valence Quality for H to Rn: Design and Assessment
of Accuracy. Phys. Chem. Chem. Phys..

[46] Tao J., Perdew J. P., Staroverov V. N., Scuseria G. E. (2003). Climbing the Density
Functional Ladder: Nonempirical Meta-Generalized Gradient Approximation
Designed for Molecules and Solids. Phys. Rev.
Lett..

[47] Staroverov V. N., Scuseria G. E., Tao J., Perdew J. P. (2003). Comparative
Assessment
of a New Nonempirical Density Functional: Molecules and Hydrogen-Bonded
Complexes. J. Chem. Phys..

[48] Staroverov V. N., Scuseria G. E., Tao J., Perdew J. P. (2004). Erratum: “Comparative
Assessment of a New Nonempirical Density Functional: Molecules and
Hydrogen-Bonded Complexes”[J. Chem. Phys. 119, 12129 (2003)]. J. Chem. Phys..

[49] Becke A. D. (1992). Density-Functional
Thermochemistry. I. The Effect of the Exchange-Only Gradient Correction. J. Phys. Chem..

[50] Barone V., Cossi M. (1998). Quantum Calculation
of Molecular Energies and Energy Gradients in
Solution by a Conductor Solvent Model. J. Phys.
Chem. A.

[51] Grimme S., Antony J., Ehrlich S., Krieg H. (2010). A Consistent and Accurate
Ab Initio Parametrization of Density Functional Dispersion Correction
(DFT-D) for the 94 Elements H-Pu. J. Chem. Phys..

[52] Grimme S., Ehrlich S., Goerigk L. (2011). Effect of
the Damping Function in
Dispersion Corrected Density Functional Theory. J. Comput. Chem..

[53] Stoychev G. L., Auer A. A., Neese F. (2017). Automatic
Generation of Auxiliary
Basis Sets. J. Chem. Theory Comput..

[54] Schleyer P. V. R., Maerker C., Dransfeld A., Jiao H., Van Eikema
Hommes N. J. R. (1996). Nucleus-Independent Chemical Shifts: A Simple and Efficient
Aromaticity Probe. J. Am. Chem. Soc..

[55] Glendening, E. D. ; Badenhoop, J. K. ; Reed, A. E. ; Carpenter, J. E. ; Bohmann, J. A. ; Morales, C. M. ; Karafiloglou, P. ; Landis, C. R. ; Weinhold, F. NBO, ver. 7.0; 2018.

[56] Humphrey W., Dalke A., Schulten K. (1996). VMD: Visual Molecular Dynamics. J. Mol. Graph..

[57] Noor A., Qayyum S., Dickert A., El Oirdi M. (2024). An Inverted-Sandwich
Dichromium­(I) Complex Stabilized by Guanidinate Ligands. Molbank.

[58] Jones C., Schulten C., Fohlmeister L., Stasch A., Murray K. S., Moubaraki B., Kohl S., Ertem M. Z., Gagliardi L., Cramer C. J. (2011). Bulky Guanidinato Nickel­(I) Complexes: Synthesis, Characterization,
Isomerization, and Reactivity Studies. Chem.
- Eur. J..

[59] Fieser M. E., MacDonald M. R., Krull B. T., Bates J. E., Ziller J. W., Furche F., Evans W. J. (2015). Structural, Spectroscopic, and Theoretical
Comparison of Traditional vs Recently Discovered Ln^2+^ Ions
in the [K­(2.2.2-Cryptand)]­[(C_5_H_4_SiMe_3_)_3_Ln] Complexes: The Variable Nature of Dy^2+^ and Nd^2+^. J. Am. Chem. Soc..

[60] Shannon R.
D. (1976). Revised
Effective Ionic Radii and Systematic Studies of Interatomic Distances
in Halides and Chalcogenides. Acta Crystallogr.
A.

[61] Edelmann F. T. (2009). Lanthanide
Amidinates and Guanidinates: From Laboratory Curiosities to Efficient
Homogeneous Catalysts and Precursors for Rare-Earth Oxide Thin Films. Chem. Soc. Rev..

[62] Edelmann F. T. (2012). Lanthanide
Amidinates and Guanidinates in Catalysis and Materials Science: A
Continuing Success Story. Chem. Soc. Rev..

[63] Allen F. H., Kennard O., Watson D. G., Brammer L., Orpen A. G., Taylor R. (1987). Tables of Bond Lengths
Determined by X-Ray and Neutron
Diffraction. Part 1. Bond Lengths in Organic Compounds. J. Chem. Soc., Perkin Trans. 2.

[64] Falceto A., Casanova D., Alemany P., Alvarez S. (2014). Distortions of π-Coordinated
Arenes with Anionic Character. Chem. - Eur.
J..

[65] Cassani M. C., Gun’ko Y. K., Hitchcock P. B., Lappert M. F., Laschi F. (1999). Synthesis
and Characterization of Organolanthanidocene­(III) (Ln = La, Ce, Pr,
Nd) Complexes Containing the 1,4-Cyclohexa-2,5-Dienyl Ligand (Benzene
1,4-Dianion): Structures of [K([18]-Crown-6)]­[Ln­{η^5^-C_5_H_3_(SiMe_3_)_2_-1,3}_2_ (C_6_H_6_)] [Cp‘‘ = η^5^-C_5_H_3_(SiMe_3_)_2_-1,3;
Ln = La, Ce, Nd]. Organometallics.

[66] Gould C. A., Marbey J., Vieru V., Marchiori D. A., David Britt R., Chibotaru L. F., Hill S., Long J. R. (2021). Isolation
of a Triplet Benzene Dianion. Nat. Chem..

[67] Dumas M. T., Ziller J. W., Evans W. J. (2019). Synthesis and Reduction of Bimetallic
Methyl-Bridged Rare-Earth Metal Complexes, [(C_5_H_4_SiMe_3_)_2_Ln­(μ-CH_3_)]_2_ (Ln = Y, Tb, Dy). ACS Omega.

[68] Orlova A. P., Hilgar J. D., Bernbeck M. G., Gembicky M., Rinehart J. D. (2022). Intuitive
Control of Low-Energy Magnetic Excitations via Directed Dipolar Interactions
in a Series of Er­(III)-Based Complexes. J. Am.
Chem. Soc..

[69] Hilgar J. D., Bernbeck M. G., Flores B. S., Rinehart J. D. (2018). Metal-Ligand Pair
Anisotropy in a Series of Mononuclear Er-COT Complexes. Chem. Sci..

[70] Benner F., Pugliese E. R., Marsden R. Q., Staples R. J., Chilton N. F., Demir S. (2024). An Organometallic Erbium
Bismuth Cluster Complex Comprising a Bi_6_
^6–^ Zintl Ion. Inorg.
Chem..

[71] Delano
IV F., Benner F., Jang S., Demir S. (2023). Pyrrolyl-Bridged Metallocene
Complexes: From Synthesis, Electronic Structure, to Single-Molecule
Magnetism. Inorg. Chem..

[72] Feltham H. L. C., Brooker S. (2014). Review of Purely 4f
and Mixed-Metal Nd-4f Single-Molecule
Magnets Containing Only One Lanthanide Ion. Coord. Chem. Rev..

[73] Rinehart J. D., Long J. R. (2011). Exploiting Single-Ion Anisotropy
in the Design of f-Element
Single-Molecule Magnets. Chem. Sci..

