# Supplementary material for: Inverse-Sandwich Rare Earth Metal Complexes Comprising a Planar Toluene Dianion
Source: Inorg Chem. 2026 Jan 3;65(2):1172–83. doi: 10.1021/acs.inorgchem.5c04418 (PMC12820926; doi:10.1021/acs.inorgchem.5c04418)
Supplement: Supplementary file 1 [file ic5c04418_si_001.pdf]

Supporting Information

for

Inverse-Sandwich Rare Earth Metal Complexes Comprising a Planar Toluene Dianion

Elizabeth R. Pugliese,§ Saroshan Deshapriya,§ Mackenzie Meyer, Ernesto Castellanos, and Selvan Demir*

Department of Chemistry, Michigan State University, 578 South Shaw Lane, East Lansing, Michigan 48824, USA

*Correspondence to: sdemir@chemistry.msu.edu (S.D.)
§These authors contributed equally.

Table of Contents

Figure S1. Examples of crystallographically confirmed toluene-bridged inverse-sandwich complexes. S6

1 **X–Ray Crystallography** **S7**

Table S1. Crystallographic data and structural refinement of $[\{(Me_3Si)_2NC(N^iPr)_2\}_2RE]_2(\mu\text{-}\eta^6:\eta^6\text{-}C_6H_5Me)$, where RE = Y (**1**), Dy (**2**), and Er (**3**). S7
Figure S2. Structure of $[\{(Me_3Si)_2NC(N^iPr)_2\}_2Y]_2(\mu\text{-}\eta^6:\eta^6\text{-}C_6H_5Me)$, **1**. S8
Figure S3. Structure of $[\{(Me_3Si)_2NC(N^iPr)_2\}_2Y]_2(\mu\text{-}\eta^6:\eta^6\text{-}C_6H_5Me)$, **1**, with thermal ellipsoids drawn at the 50% probability level. S9
Figure S4. Space-filling model of $[\{(Me_3Si)_2NC(N^iPr)_2\}_2Y]_2(\mu\text{-}\eta^6:\eta^6\text{-}C_6H_5Me)$, **1**. S10
Figure S5. Unit cell of $[\{(Me_3Si)_2NC(N^iPr)_2\}_2Y]_2(\mu\text{-}\eta^6:\eta^6\text{-}C_6H_5Me)$, **1**, along the a-axis. S11
Figure S6. Unit cell of $[\{(Me_3Si)_2NC(N^iPr)_2\}_2Y]_2(\mu\text{-}\eta^6:\eta^6\text{-}C_6H_5Me)$, **1**, along the b-axis. S12
Figure S7. Unit cell of $[\{(Me_3Si)_2NC(N^iPr)_2\}_2Y]_2(\mu\text{-}\eta^6:\eta^6\text{-}C_6H_5Me)$, **1**, along the c-axis. S13
Figure S8. Crystal packing diagram of $[\{(Me_3Si)_2NC(N^iPr)_2\}_2Y]_2(\mu\text{-}\eta^6:\eta^6\text{-}C_6H_5Me)$, **1**, along the a-axis. S14
Figure S9. Crystal packing diagram of $[\{(Me_3Si)_2NC(N^iPr)_2\}_2Y]_2(\mu\text{-}\eta^6:\eta^6\text{-}C_6H_5Me)$, **1**, along the b-axis. S15
Figure S10. Crystal packing diagram of $[\{(Me_3Si)_2NC(N^iPr)_2\}_2Y]_2(\mu\text{-}\eta^6:\eta^6\text{-}C_6H_5Me)$, **1**, along the c-axis. S16
Figure S11. Structure of $[\{(Me_3Si)_2NC(N^iPr)_2\}_2Dy]_2(\mu\text{-}\eta^6:\eta^6\text{-}C_6H_5Me)$, **2**. S17
Figure S12. Structure of $[\{(Me_3Si)_2NC(N^iPr)_2\}_2Dy]_2(\mu\text{-}\eta^6:\eta^6\text{-}C_6H_5Me)$, **2**, with thermal ellipsoids drawn at the 50% probability level. S18
Figure S13. Space-filling model of $[\{(Me_3Si)_2NC(N^iPr)_2\}_2Dy]_2(\mu\text{-}\eta^6:\eta^6\text{-}C_6H_5Me)$, **2**. S19
Figure S14. Unit cell of $[\{(Me_3Si)_2NC(N^iPr)_2\}_2Dy]_2(\mu\text{-}\eta^6:\eta^6\text{-}C_6H_5Me)$, **2**, along the a-axis. S20
Figure S15. Unit cell of $[\{(Me_3Si)_2NC(N^iPr)_2\}_2Dy]_2(\mu\text{-}\eta^6:\eta^6\text{-}C_6H_5Me)$, **2**, along the b-axis. S21
Figure S16. Unit cell of $[\{(Me_3Si)_2NC(N^iPr)_2\}_2Dy]_2(\mu\text{-}\eta^6:\eta^6\text{-}C_6H_5Me)$, **2**, along the c-axis. S22
Figure S17. Crystal packing diagram of $[\{(Me_3Si)_2NC(N^iPr)_2\}_2Dy]_2(\mu\text{-}\eta^6:\eta^6\text{-}C_6H_5Me)$, **2**, along the a-axis. S23
Figure S18. Crystal packing diagram of $[\{(Me_3Si)_2NC(N^iPr)_2\}_2Dy]_2(\mu\text{-}\eta^6:\eta^6\text{-}C_6H_5Me)$, **2**, along the b-axis. S24
Figure S19. Crystal packing diagram of $[\{(Me_3Si)_2NC(N^iPr)_2\}_2Dy]_2(\mu\text{-}\eta^6:\eta^6\text{-}C_6H_5Me)$, **2**, along the c-axis. S25
Figure S20. Structure of $[\{(Me_3Si)_2NC(N^iPr)_2\}_2Er]_2(\mu\text{-}\eta^6:\eta^6\text{-}C_6H_5Me)$, **3**, S26

with thermal ellipsoids drawn at the 50% probability level.
Figure S21. Space-filling model of
[{$(Me_3Si)_2NC(N^iPr)_2\}_2Er]_2(\mu\text{-}\eta^6:\eta^6\text{-}C_6H_5Me)$, **3**. S27
Figure S22. Unit cell of [{$(Me_3Si)_2NC(N^iPr)_2\}_2Er]_2(\mu\text{-}\eta^6:\eta^6\text{-}C_6H_5Me)$, **3**, along the a-axis. S28
Figure S23. Unit cell of [{$(Me_3Si)_2NC(N^iPr)_2\}_2Er]_2(\mu\text{-}\eta^6:\eta^6\text{-}C_6H_5Me)$, **3**, along the b-axis. S29
Figure S24. Unit cell of [{$(Me_3Si)_2NC(N^iPr)_2\}_2Er]_2(\mu\text{-}\eta^6:\eta^6\text{-}C_6H_5Me)$, **3**, along the c-axis. S30
Figure S25. Crystal packing diagram of
[{$(Me_3Si)_2NC(N^iPr)_2\}_2Er]_2(\mu\text{-}\eta^6:\eta^6\text{-}C_6H_5Me)$, **3**, along the a-axis. S31
Figure S26. Crystal packing diagram of
[{$(Me_3Si)_2NC(N^iPr)_2\}_2Er]_2(\mu\text{-}\eta^6:\eta^6\text{-}C_6H_5Me)$, **3**, along the b-axis. S32
Figure S27. Crystal packing diagram of
[{$(Me_3Si)_2NC(N^iPr)_2\}_2Er]_2(\mu\text{-}\eta^6:\eta^6\text{-}C_6H_5Me)$, **3**, along the c-axis. S33

2 NMR Spectroscopy S34

Figure S28. ^1H NMR spectrum of
[{$(Me_3Si)_2NC(N^iPr)_2\}_2Y]_2(\mu\text{-}\eta^6:\eta^6\text{-}C_6H_5Me)$, **1.** S34
Figure S29. ^{13}C NMR spectrum of
[{$(Me_3Si)_2NC(N^iPr)_2\}_2Y]_2(\mu\text{-}\eta^6:\eta^6\text{-}C_6H_5Me)$, **1.** S36
Figure S30. ^1H NMR spectrum of
[{$(Me_3Si)_2NC(N^iPr)_2\}_2Dy]_2(\mu\text{-}\eta^6:\eta^6\text{-}C_6H_5Me)$, **2**. S36
Figure S31. ^1H NMR spectrum of
[{$(Me_3Si)_2NC(N^iPr)_2\}_2Er]_2(\mu\text{-}\eta^6:\eta^6\text{-}C_6H_5Me)$, **3**. S37

3 IR Spectroscopy S38

Figure S32. FTIR spectrum of
[{$(Me_3Si)_2NC(N^iPr)_2\}_2Y]_2(\mu\text{-}\eta^6:\eta^6\text{-}C_6H_5Me)$, **1**. S38
Figure S33. FTIR spectrum of
[{$(Me_3Si)_2NC(N^iPr)_2\}_2Dy]_2(\mu\text{-}\eta^6:\eta^6\text{-}C_6H_5Me)$, **2**. S39
Figure S34. FTIR spectrum of
[{$(Me_3Si)_2NC(N^iPr)_2\}_2Er]_2(\mu\text{-}\eta^6:\eta^6\text{-}C_6H_5Me)$, **3**. S40

4 UV–Vis Spectroscopy S41

Figure S35. UV-vis spectrum of
[{$(Me_3Si)_2NC(N^iPr)_2\}_2Y]_2(\mu\text{-}\eta^6:\eta^6\text{-}C_6H_5Me)$, **1**. S41
Figure S36. UV-vis spectrum of
[{$(Me_3Si)_2NC(N^iPr)_2\}_2Dy]_2(\mu\text{-}\eta^6:\eta^6\text{-}C_6H_5Me)$, **2**. S42
Figure S37. UV-vis spectrum of
[{$(Me_3Si)_2NC(N^iPr)_2\}_2Er]_2(\mu\text{-}\eta^6:\eta^6\text{-}C_6H_5Me)$, **3**. S43
Figure S38. Magnification of the UV-vis spectra of
[{$(Me_3Si)_2NC(N^iPr)_2\}_2RE]_2(\mu\text{-}\eta^6:\eta^6\text{-}C_6H_5Me)$, where RE = Y (**1**), Dy (**2**), S44

and Er (**3**).

5 Magnetic Measurements S45

Figure S39. Variable-temperature dc magnetic susceptibility data of $[\{(Me_3Si)_2NC(N^iPr)_2\}_2Dy]_2(\mu-\eta^6:\eta^6-C_6H_5Me)$, **2**, under a 0.1 T applied dc field. S45
Figure S40. Variable-temperature dc magnetic susceptibility data of $[\{(Me_3Si)_2NC(N^iPr)_2\}_2Dy]_2(\mu-\eta^6:\eta^6-C_6H_5Me)$, **2**, under a 1.0 T applied dc field. S46
Figure S41. Variable-temperature dc magnetic susceptibility data of $[\{(Me_3Si)_2NC(N^iPr)_2\}_2Er]_2(\mu-\eta^6:\eta^6-C_6H_5Me)$, **3**, under a 0.01 T applied dc field. S47
Figure S42. Variable-temperature dc magnetic susceptibility data of $[\{(Me_3Si)_2NC(N^iPr)_2\}_2Er]_2(\mu-\eta^6:\eta^6-C_6H_5Me)$, **3**, under a 0.1 T applied dc field. S48
Figure S43. Variable-temperature dc magnetic susceptibility data of $[\{(Me_3Si)_2NC(N^iPr)_2\}_2Er]_2(\mu-\eta^6:\eta^6-C_6H_5Me)$, **3**, under a 0.5 T applied dc field. S49
Figure S44. Variable-temperature dc magnetic susceptibility data of $[\{(Me_3Si)_2NC(N^iPr)_2\}_2Er]_2(\mu-\eta^6:\eta^6-C_6H_5Me)$, **3**, under a 1.0 T applied dc field. S50
Figure S45. Variable-temperature dc magnetic susceptibility data of $[\{(Me_3Si)_2NC(N^iPr)_2\}_2Er]_2(\mu-\eta^6:\eta^6-C_6H_5Me)$, **3**, under 0.01 T, 0.1 T, 0.5 T, and 1.0 T applied dc fields. S51
Figure S46. Field-dependent magnetization data for $[\{(Me_3Si)_2NC(N^iPr)_2\}_2Dy]_2(\mu-\eta^6:\eta^6-C_6H_5Me)$, **2**, collected from 0 to 7 T between 2 K and 10 K. S52
Figure S47. Field-dependent magnetization data for $[\{(Me_3Si)_2NC(N^iPr)_2\}_2Er]_2(\mu-\eta^6:\eta^6-C_6H_5Me)$, **3**, collected from 0 to 7 T between 2 K and 10 K. S53
Figure S48. Field-dependent reduced magnetization data for $[\{(Me_3Si)_2NC(N^iPr)_2\}_2Er]_2(\mu-\eta^6:\eta^6-C_6H_5Me)$, **3**, collected from 0 to 7 T between 2 K and 10 K. S54
Figure S49. Out-of-phase (χ_M'') components of the ac magnetic susceptibility for $[\{(Me_3Si)_2NC(N^iPr)_2\}_2Dy]_2(\mu-\eta^6:\eta^6-C_6H_5Me)$, **2**, at 1.8 K under dc fields ranging from 0 Oe to 2000 Oe. S55
Figure S50. In-phase (χ_M') components of the ac magnetic susceptibility for $[\{(Me_3Si)_2NC(N^iPr)_2\}_2Dy]_2(\mu-\eta^6:\eta^6-C_6H_5Me)$, **2**, under a 1500 Oe applied dc field. S56
Figure S51. Out-of-phase (χ_M'') components of the ac magnetic susceptibility for $[\{(Me_3Si)_2NC(N^iPr)_2\}_2Dy]_2(\mu-\eta^6:\eta^6-C_6H_5Me)$, **2**, under a 1500 Oe applied dc field. S57
Figure S52. Out-of-phase (χ_M'') components of the ac magnetic susceptibility for $[\{(Me_3Si)_2NC(N^iPr)_2\}_2Er]_2(\mu-\eta^6:\eta^6-C_6H_5Me)$, **3**, at 1.8 K S58

under dc fields ranging from 0 Oe to 3000 Oe.
Figure S53. In-phase (χ_M') components of the ac magnetic susceptibility for [{$(Me_3Si)_2NC(N^iPr)_2$}$_2$Er]$_2$(μ-η^6:η^6-C_6H_5Me), **2**, under a 500 Oe applied dc field. S59
Figure S54. Plot of magnetization (*M*) vs dc magnetic field (*H*) for [{$(Me_3Si)_2NC(N^iPr)_2$}$_2$Dy]$_2$(μ-η^6:η^6-C_6H_5Me), **2**, at 1.8 K. S60
Figure S55. Plot of magnetization (*M*) vs dc magnetic field (*H*) for [{$(Me_3Si)_2NC(N^iPr)_2$}$_2$Er]$_2$(μ-η^6:η^6-C_6H_5Me), **3**, at 1.8 K. S61

6 DFT Calculations S62

Figure S56. DFT-calculated C–C bond distances and natural charge distribution on the bridging toluene unit of [{$(Me_3Si)_2NC(N^iPr)_2$}$_2$Y]$_2$(μ-η^6:η^6-C_6H_5Me), **1**. S62
Table S2. DFT-calculated bond metrics of [{$(Me_3Si)_2NC(N^iPr)_2$}$_2$Y]$_2$(μ-η^6:η^6-C_6H_5Me), **1**. S63
Table S3. Major TD-DFT transitions calculated for [{$(Me_3Si)_2NC(N^iPr)_2$}$_2$Y]$_2$(μ-η^6:η^6-C_6H_5Me), **1**. S64
Table S4. DFT-optimized coordinates of [{$(Me_3Si)_2NC(N^iPr)_2$}$_2$Y]$_2$(μ-η^6:η^6-C_6H_5Me), **1**. S66

7 References S71

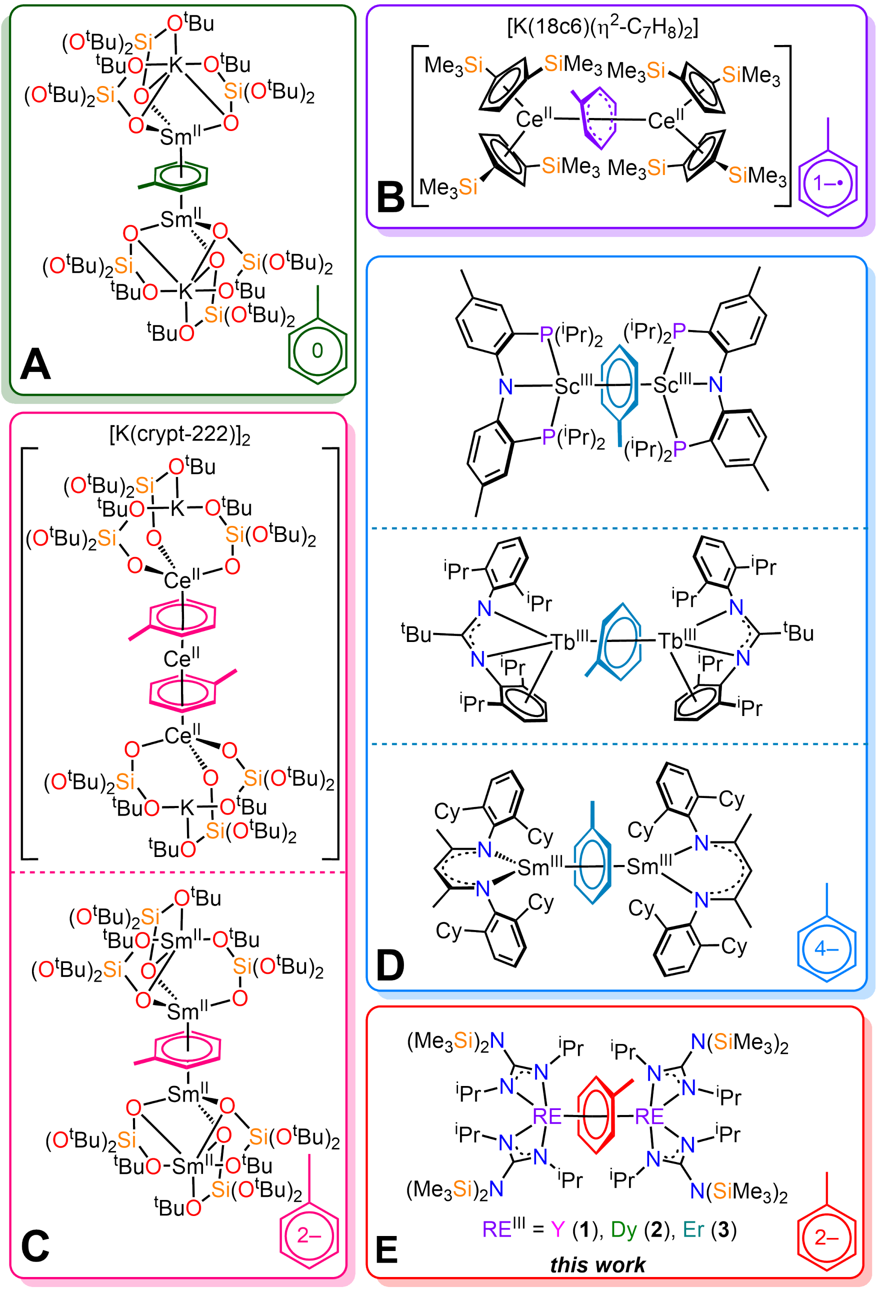

Figure S1. All crystallographically confirmed dinuclear toluene-bridged rare earth metal complexes. (**A**) [{KSm(OSi(O^{t}Bu)$_3$)$_3$}$_2$(μ-η^6:η^6-C$_6$H$_5$Me)], featuring a neutral toluene bridge;[1] (**B**) [K(18-c-6)(η^2-PhMe)$_2$] [(CeCp″$_2$)$_2$(μ-η^6:η^6-C$_6$H$_5$Me)], containing a monoanionic toluene bridge;[2] (**C**) [K(2.2.2-crypt)]$_2$[{(KCe(OSi(O^{t}Bu)$_3$)$_3$)(μ-η^6:η^6-C$_6$H$_5$Me)}$_2$Ce][3] and [{KSm(OSi(O^{t}Bu)$_3$)$_3$}$_2$(μ-η^6:η^6-C$_6$H$_5$Me)],[1] comprising dianionic toluene bridges; (**D**) [{(PNP)Sc}$_2$(μ-η^6:η^6-C$_6$H$_5$Me)],[4] [{(BDI^{Dicyp})Sm}$_2$(μ-η^6:η^6-C$_6$H$_5$Me)],[5] and [{Tb(κ^1:η^6-Piso)}$_2$(μ-η^6:η^6-C$_6$H$_5$Me)],[6] exhibiting tetraanionic toluene bridges; (**E**) This work, [{(Me$_3$Si)$_2$NC(N^{i}Pr)$_2$}$_2$RE]$_2$(μ-η^6:η^6-C$_6$H$_5$Me), where RE = Y (**1**), Dy (**2**), Er (**3**), featuring a dianionic toluene bridge. Abbreviations: BDI^{Dicyp} = CH[C(CH$_3$)NDicyp]$_2$; Dicyp = 2,6-dicyclohexylphenyl; Piso = {N(2,6-(^{i}Pr)$_2$C$_6$H$_3$)}$_2$C^{t}Bu; PNP = N(C$_6$H$_3$-2-P^{i}Pr$_2$-4-CH$_3$)$_2$.

1 X–Ray Crystallography

Table S1. Crystallographic data and structural refinement of [{$(Me_3Si)_2NC(N^iPr)_2$}$_2$RE]$_2$(μ-η^6:η^6-C_6H_5Me), where RE = Y (**1**), Dy (**2**), Er (**3**).

	**1**	**2**	**3**
CCDC Number	2490023	2490024	2490025
Empirical formula	$C_{59}H_{136}Y_2N_{12}Si_8$	$C_{59}H_{136}Dy_2N_{12}Si_8$	$C_{59}H_{136}Er_2N_{12}Si_8$
Formula weight	1416.33	1563.51	1573.03
Temperature/K	100.00(10)	100.00(11)	100.00(11)
Crystal system	triclinic	triclinic	triclinic
Space group	*P*-1	*P*-1	*P*-1
a/Å	9.8039(2)	9.8060(3)	9.7974(2)
b/Å	13.8271(3)	13.8364(4)	13.8072(3)
c/Å	15.3196(4)	15.4042(6)	15.3076(4)
α/°	83.498(2)	83.670(3)	83.675(2)
β/°	88.250(2)	88.128(3)	87.962(2)
γ/°	77.623(2)	77.425(3)	77.467(2)
Volume/Å^3	2015.36(8)	2027.38(12)	2008.95(9)
Z	1	1	1
ρ_{calc}g/cm^3	1.167	1.281	1.300
μ/mm^{-1}	3.366	11.178	5.208
F(000)	764.0	818.0	822.0
Crystal size/mm^3	0.377 × 0.242 × 0.116	0.09 × 0.07 × 0.02	0.122 × 0.066 × 0.058
Radiation	Cu Kα (λ = 1.54184)	Cu Kα (λ = 1.54184)	Cu Kα (λ = 1.54184)
2*Θ* range for data collection/°	5.806 to 160.822	5.772 to 149	5.81 to 160.77
Index ranges	-11 ≤ h ≤ 12, -17 ≤ k ≤ 16, -19 ≤ l ≤ 19	-12 ≤ h ≤ 12, -14 ≤ k ≤ 17, -19 ≤ l ≤ 19	-12 ≤ h ≤ 12, -17 ≤ k ≤ 15, -19 ≤ l ≤ 19
Reflections collected	28266	27889	28146
Independent reflections	8545 [R_{int} = 0.0540, R_{sigma} = 0.0482]	8179 [R_{int} = 0.0586, R_{sigma} = 0.0528]	8512 [R_{int} = 0.0506, R_{sigma} = 0.0497]
Data/restraints/parameters	8545/0/549	8179/0/390	8512/0/399
Goodness-of-fit on F^2	1.053	1.112	1.068
Final R indexes [I>=2σ (I)]	R_1 = 0.0454, wR_2 = 0.1200	R_1 = 0.0497, wR_2 = 0.1302	R_1 = 0.0419, wR_2 = 0.0952
Final R indexes [all data]	R_1 = 0.0476, wR_2 = 0.1222	R_1 = 0.0609, wR_2 = 0.1376	R_1 = 0.0478, wR_2 = 0.0975
Largest diff. peak/hole / e Å^{-3}	0.86/-0.90	0.90/-1.85	0.78/-1.51

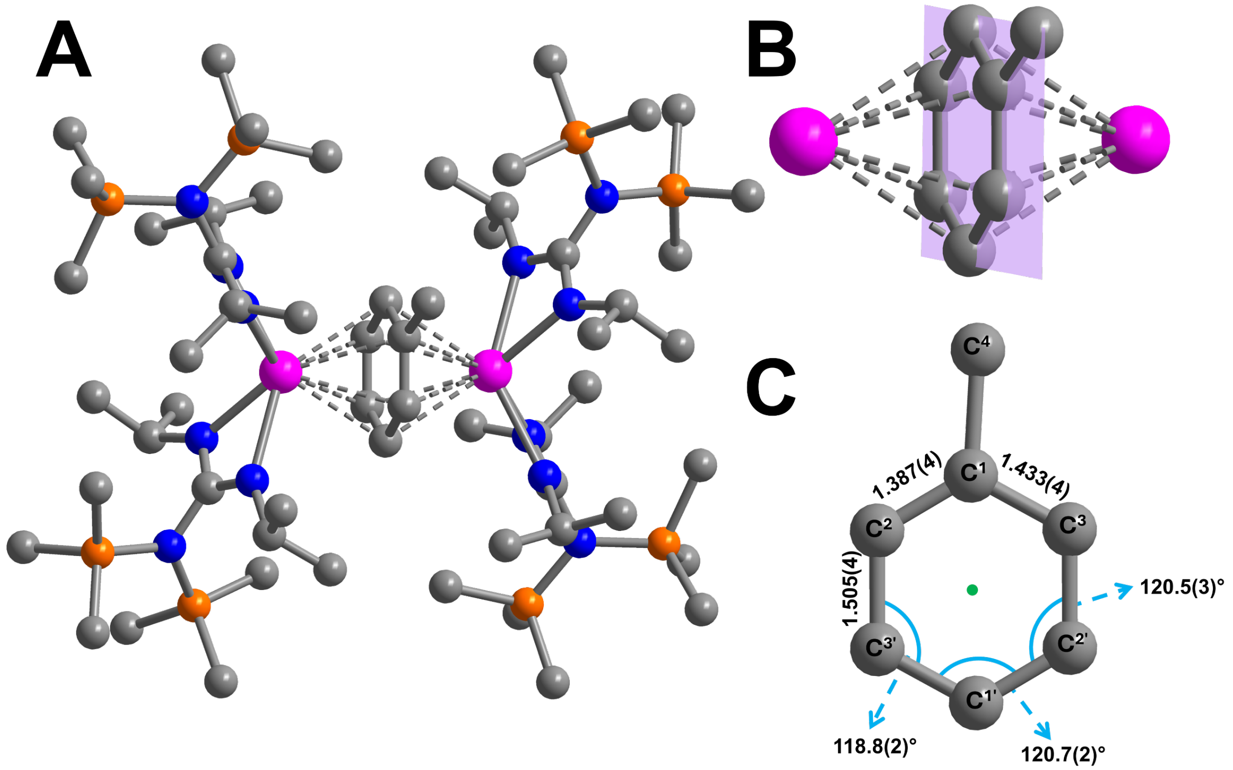

Figure S2. (**A**) Structure of [{$(Me_3Si)_2NC(N^iPr)_2$}$_2$Y]$_2$(μ-η^6:η^6-C_6H_5Me), **1**. Pink, orange, blue, and gray spheres represent Y, Si, N, and C atoms, respectively. H atoms have been omitted for clarity. Only one orientation of the disordered toluene dianion is shown for clarity. (**B**) Inverse-sandwich core of **1** enlarged with a reference plane through the phenyl moiety of toluene. Pink and gray spheres represent Y and C atoms, respectively. Ancillary guanidinate ligands and H atoms have been omitted for clarity. The mean plane is shown in purple. (**C**) Dianionic toluene enlarged with distances (Å), angles (°), and corresponding atoms labeled. The pale green mark represents the inversion center. Only one orientation of the disordered toluene dianion is shown for clarity also for (**B**) and (**C**).

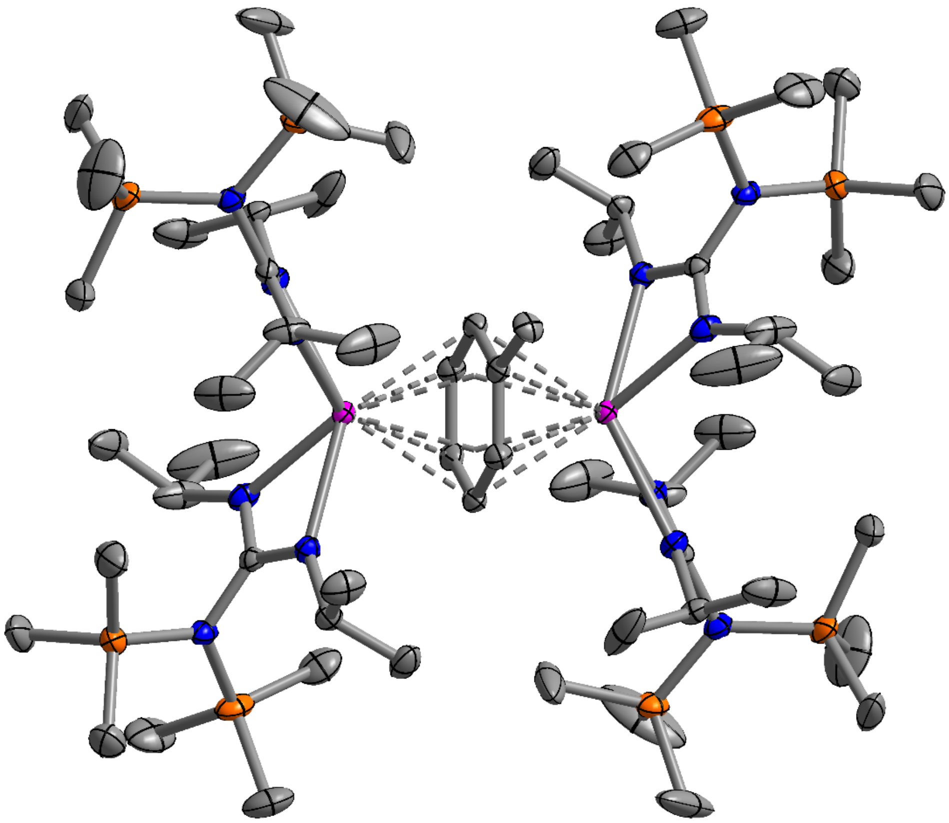

Figure S3. Structure of $[\{(Me_3Si)_2NC(N^iPr)_2\}_2Y]_2(\mu\text{-}\eta^6\text{:}\eta^6\text{-}C_6H_5Me)$, **1**, with thermal ellipsoids drawn at the 50% probability level. Pink, orange, blue, and gray spheres represent Y, Si, N, and C atoms, respectively. H atoms have been omitted for clarity. Only one orientation of the disordered toluene dianion is shown for clarity.

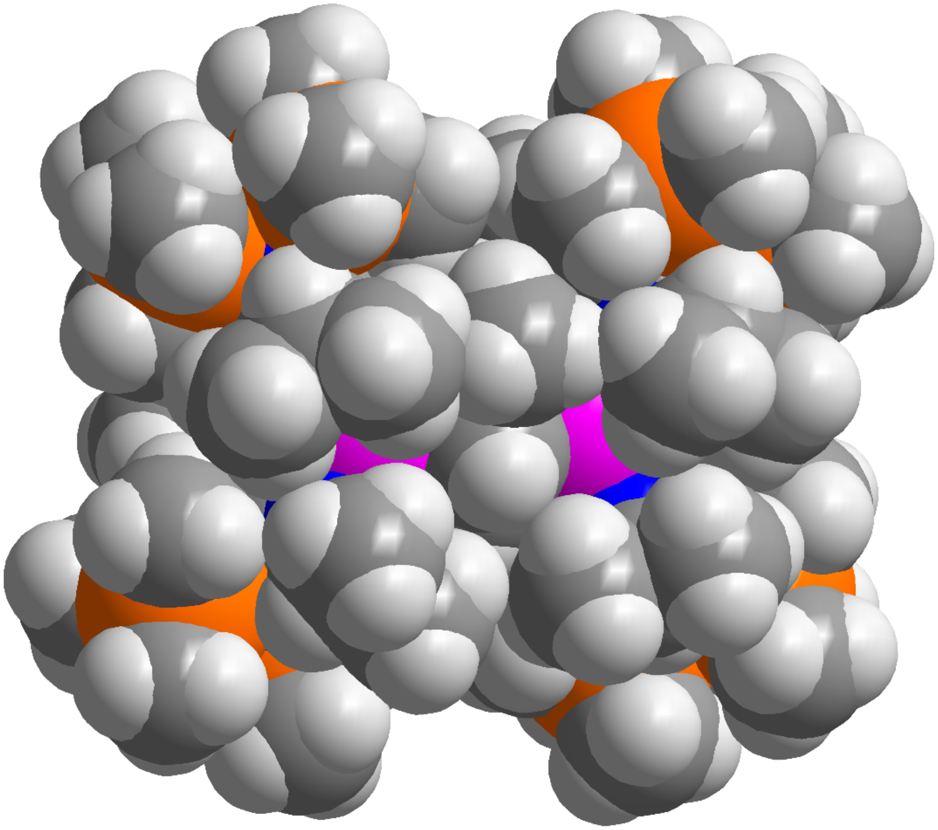

Figure S4. Space-filling model of [{(Me$_3$Si)$_2$NC(N^{i}Pr)$_2$}$_2$Y]$_2$(μ-η^6:η^6-C$_6$H$_5$Me), **1**. Pink, orange, blue, gray, and white-gray spheres represent Y, Si, N, C, and H atoms, respectively. Only one orientation of the disordered toluene dianion is shown for clarity.

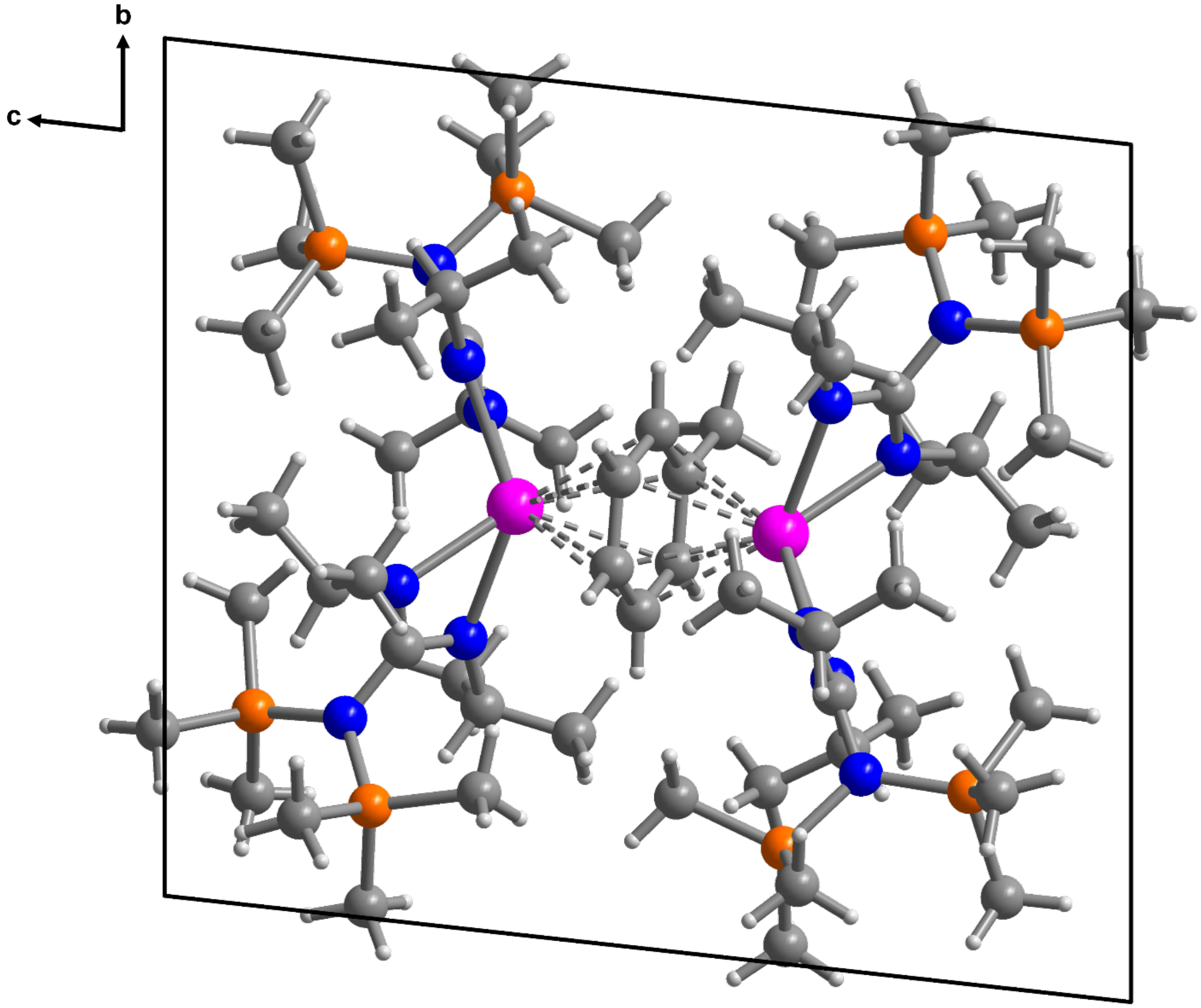

Figure S5. Unit cell of $[\{(Me_3Si)_2NC(N^iPr)_2\}_2Y]_2(\mu\text{-}\eta^6\text{:}\eta^6\text{-}C_6H_5Me)$, **1**, along the a-axis. Pink, orange, blue, gray, and white-gray spheres represent Y, Si, N, C, and H atoms, respectively. Only one orientation of the disordered toluene dianion is shown for clarity. Unit cell edges are shown as black lines.

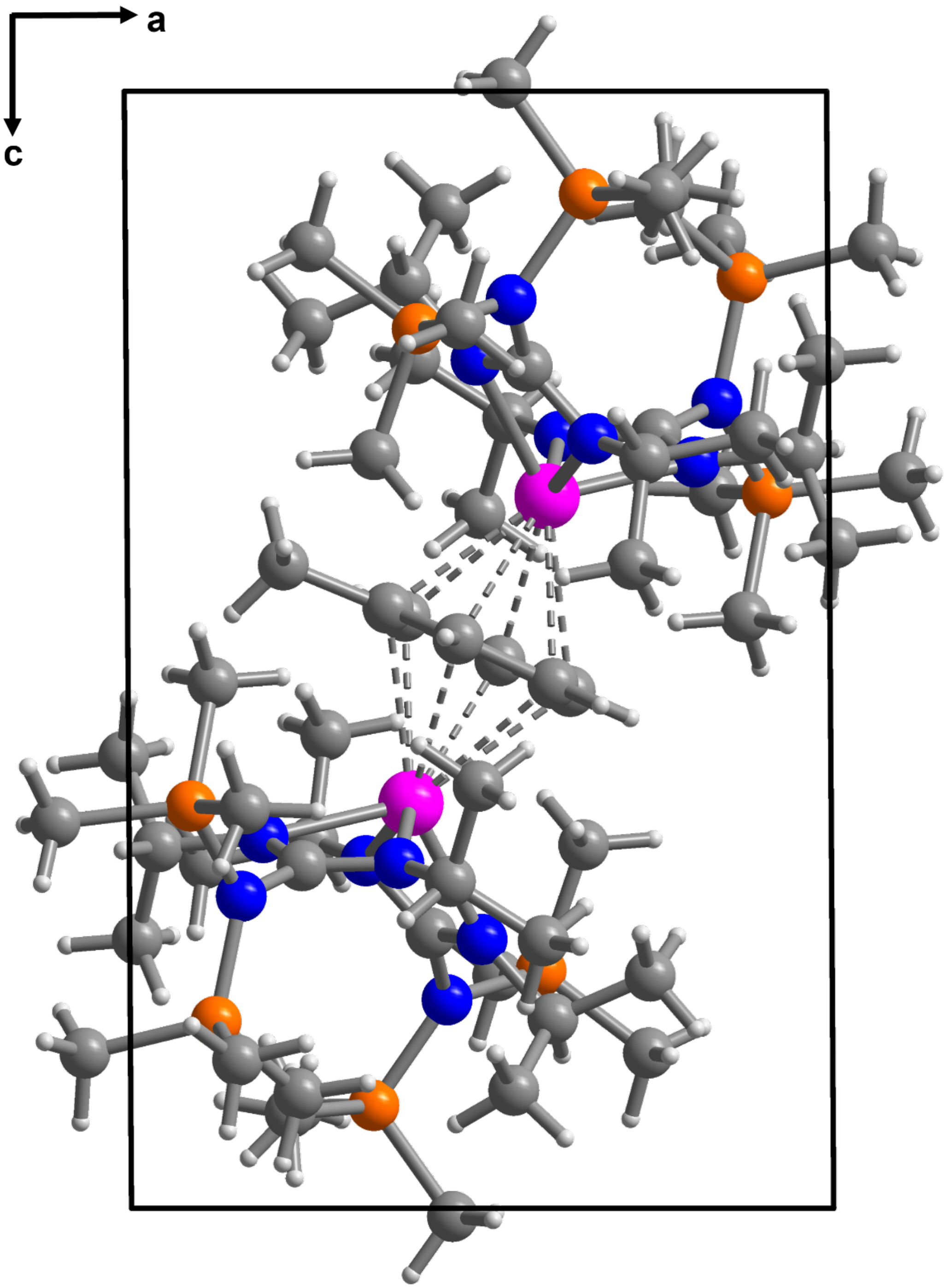

Figure S6. Unit cell of $[\{(Me_3Si)_2NC(N^iPr)_2\}_2Y]_2(\mu\text{-}\eta^6:\eta^6\text{-}C_6H_5Me)$, **1**, along the b-axis. Pink, orange, blue, gray, and white-gray spheres represent Y, Si, N, C, and H atoms, respectively. Only one orientation of the disordered toluene dianion is shown for clarity. Unit cell edges are shown as black lines.

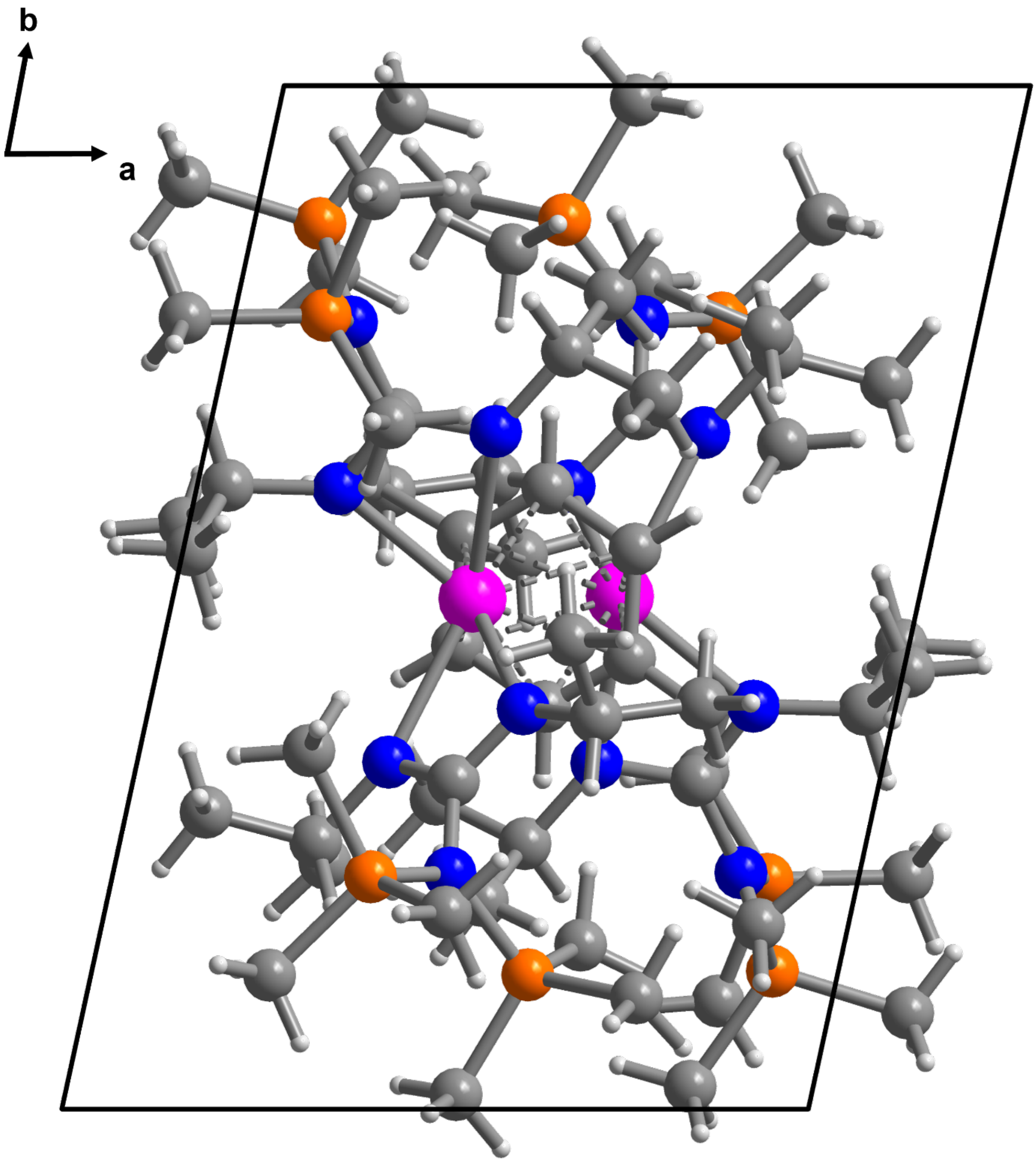

Figure S7. Unit cell of [{(Me_3Si)$_2$NC(N^{i}Pr)$_2$}$_2$Y]$_2$(μ-η^6:η^6-C_6H_5Me), **1**, along the c-axis. Pink, orange, blue, gray, and white-gray spheres represent Y, Si, N, C, and H atoms, respectively. Only one orientation of the disordered toluene dianion is shown for clarity. Unit cell edges are shown as black lines.

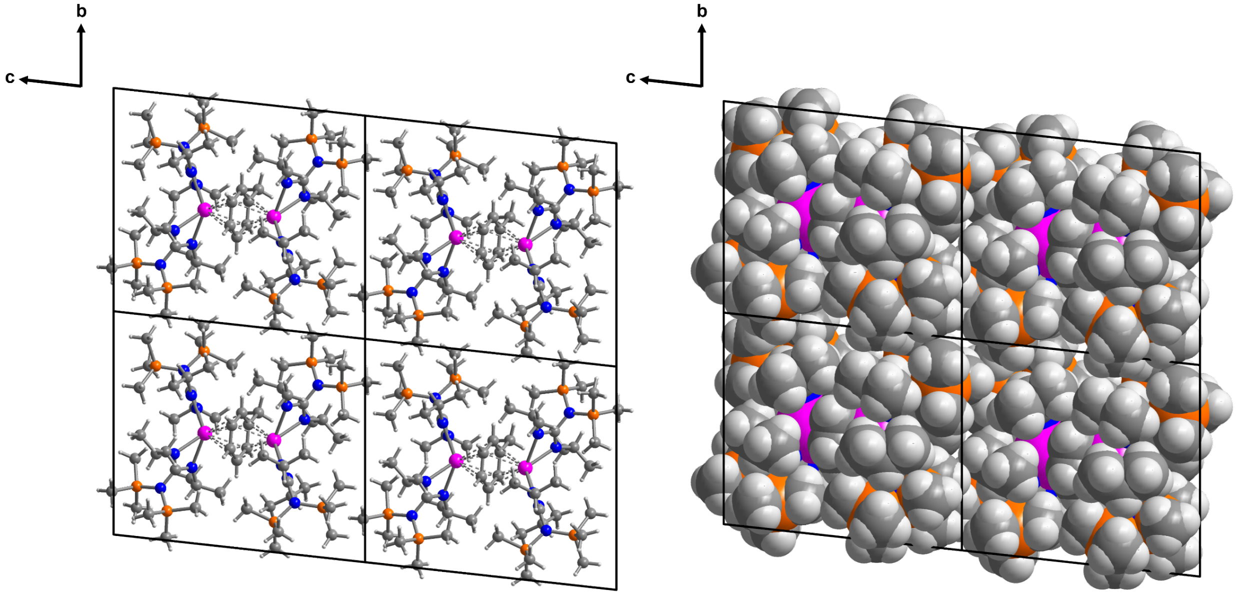

Figure S8. Crystal packing diagram of [{$(Me_3Si)_2NC(N^iPr)_2$}$_2$Y]$_2$(μ-η^6:η^6-C_6H_5Me), **1**, along the a-axis with ball-and-stick (left) and space-filling (right) representations. Pink, orange, blue, gray, and white-gray spheres represent Y, Si, N, C, and H atoms, respectively. Only one orientation of the disordered toluene dianion is shown for clarity. Unit cell edges are shown as black lines.

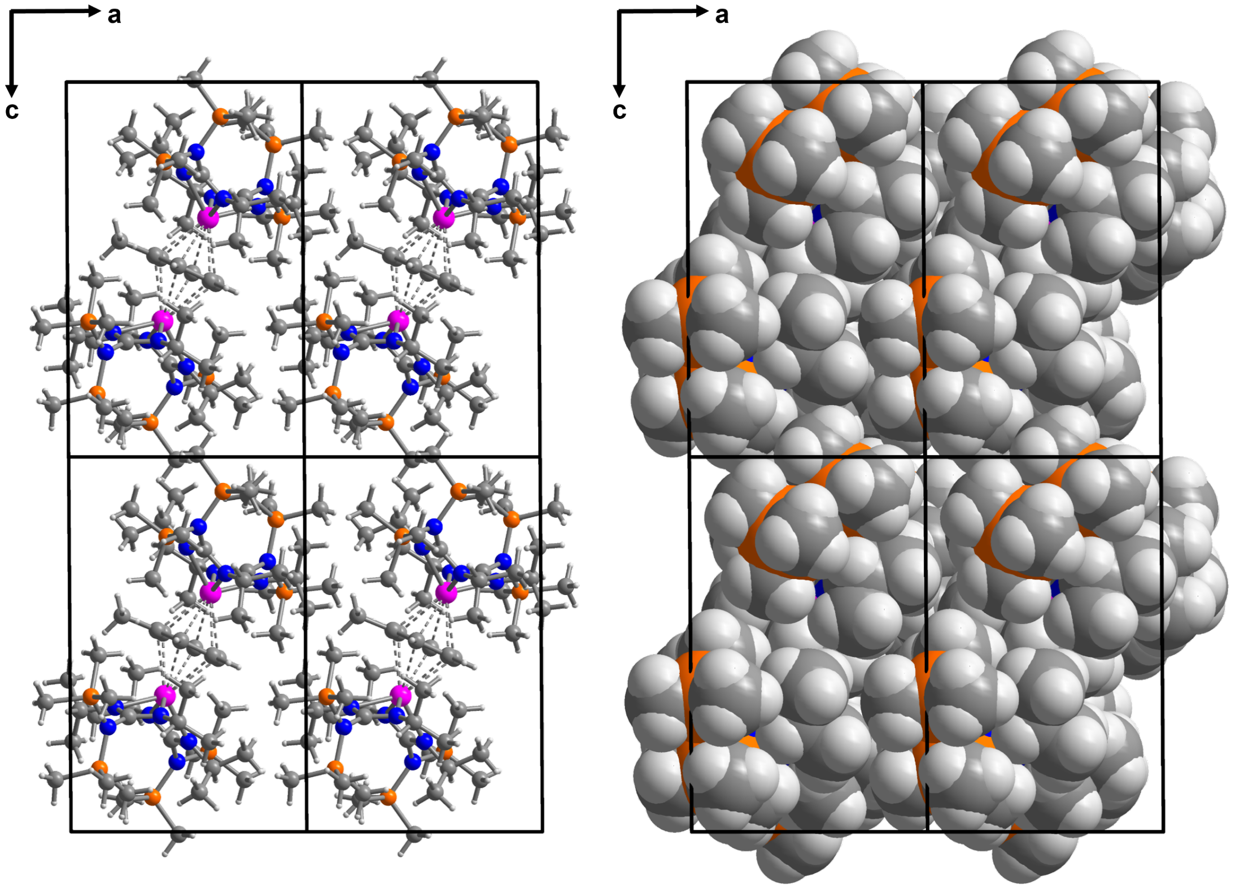

Figure S9. Crystal packing diagram of $[\{(Me_3Si)_2NC(N^iPr)_2\}_2Y]_2(\mu\text{-}\eta^6\text{:}\eta^6\text{-}C_6H_5Me)$, **1**, along the b-axis with ball-and-stick (left) and space-filling (right) representations. Pink, orange, blue, gray, and white-gray spheres represent Y, Si, N, C, and H atoms, respectively. Only one orientation of the disordered toluene dianion is shown for clarity. Unit cell edges are shown as black lines.

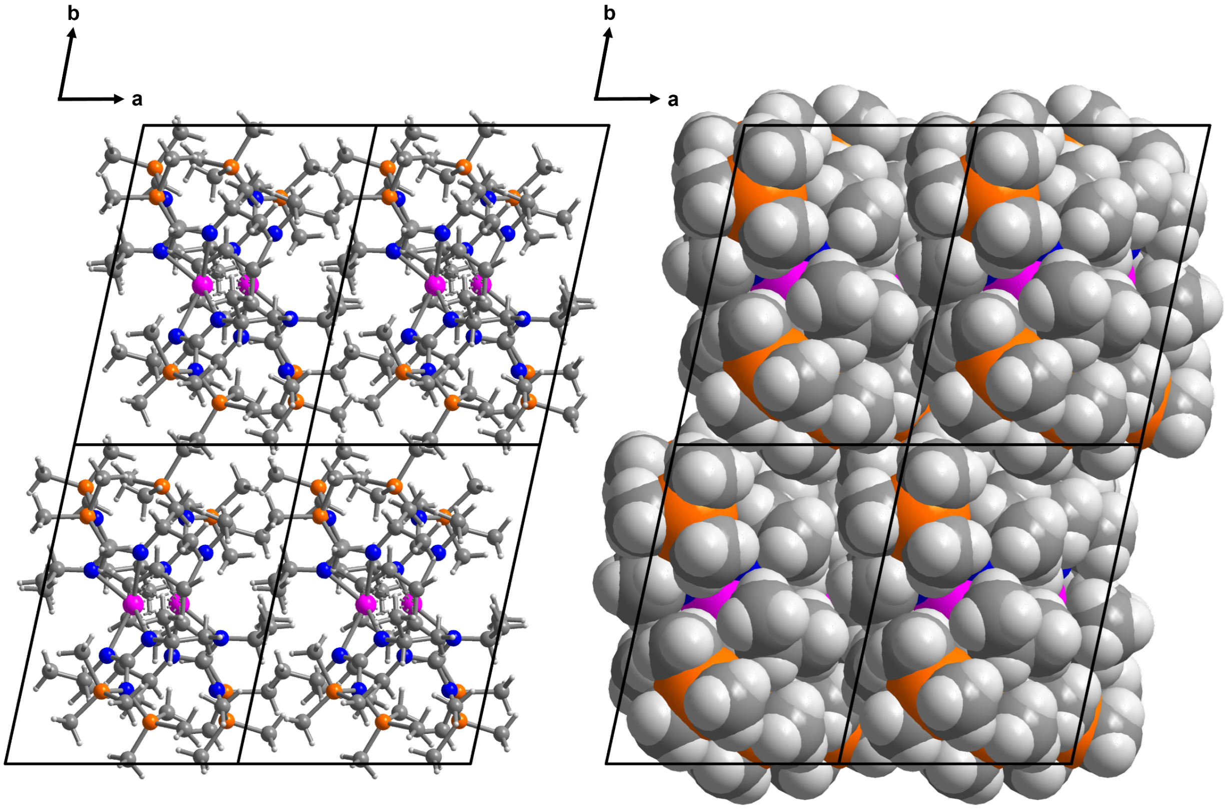

Figure S10. Crystal packing diagram of $[\{(Me_3Si)_2NC(N^iPr)_2\}_2Y]_2(\mu\text{-}\eta^6\text{:}\eta^6\text{-}C_6H_5Me)$, **1**, along the c-axis with ball-and-stick (left) and space-filling (right) representations. Pink, orange, blue, gray, and white-gray spheres represent Y, Si, N, C, and H atoms, respectively. Only one orientation of the disordered toluene dianion is shown for clarity. Unit cell edges are shown as black lines.

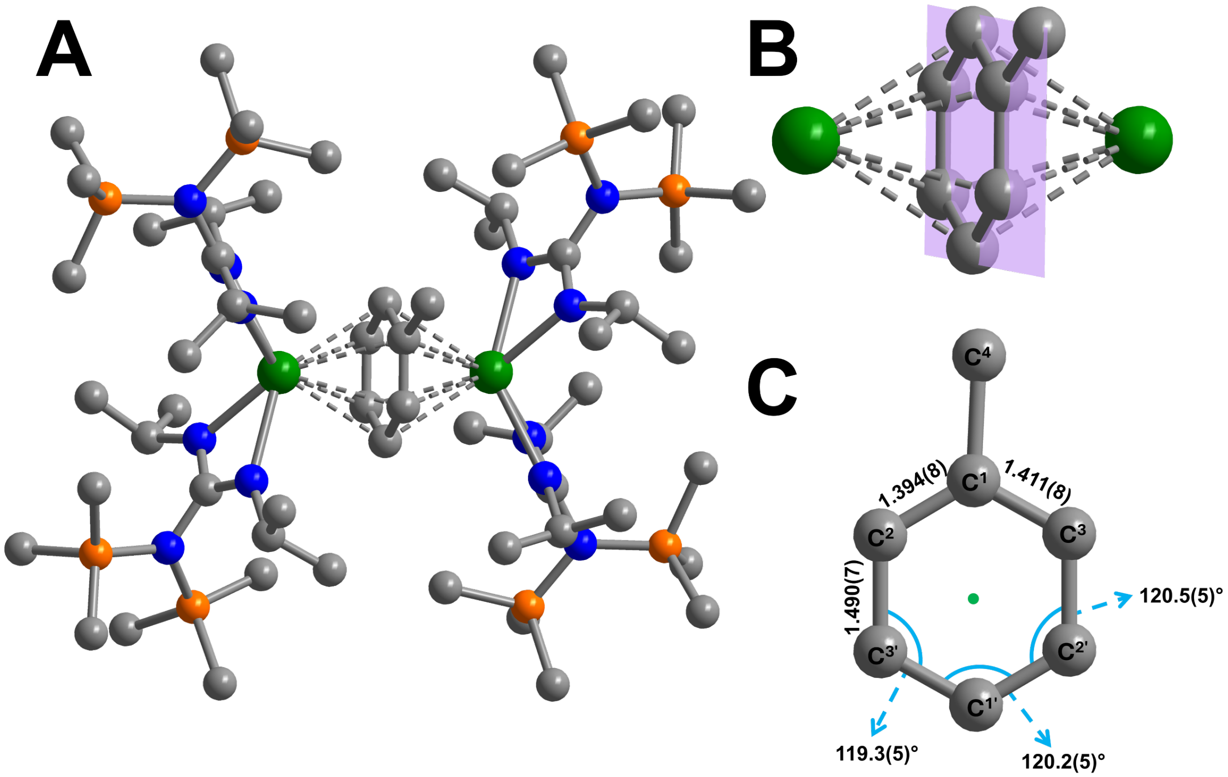

Figure S11. (**A**) Structure of [{$(Me_3Si)_2NC(N^iPr)_2\}_2Dy]_2(\mu-\eta^6:\eta^6-C_6H_5Me)$, **2**. Green, orange, blue, and gray spheres represent Dy, Si, N, and C atoms, respectively. H atoms have been omitted for clarity. Only one orientation of the disordered toluene dianion is shown for clarity. (**B**) Inverse-sandwich core of **2** enlarged with a reference plane through the phenyl moiety of toluene. Green and gray spheres represent Dy and C atoms, respectively. Ancillary guanidinate ligands and H atoms have been omitted for clarity. The mean plane is shown in purple. (**C**) Dianionic toluene enlarged with distances (Å), angles (°), and corresponding atoms labeled. The pale green mark represents the inversion center. Only one orientation of the disordered toluene dianion is shown for clarity also for (**B**) and (**C**).

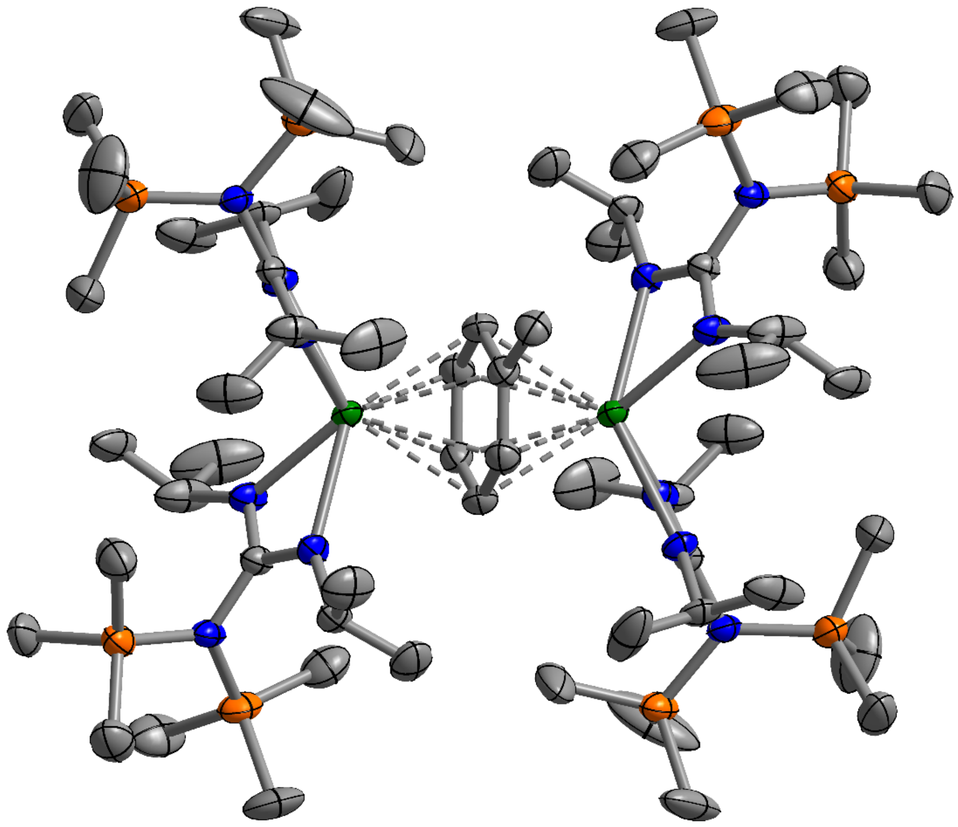

Figure S12. Structure of $[\{(Me_3Si)_2NC(N^iPr)_2\}_2Dy]_2(\mu\text{-}\eta^6{:}\eta^6\text{-}C_6H_5Me)$, **2**, with thermal ellipsoids drawn at the 50% probability level. Green, orange, blue, and gray spheres represent Dy, Si, N, and C atoms, respectively. H atoms have been omitted for clarity. Only one orientation of the disordered toluene dianion is shown for clarity.

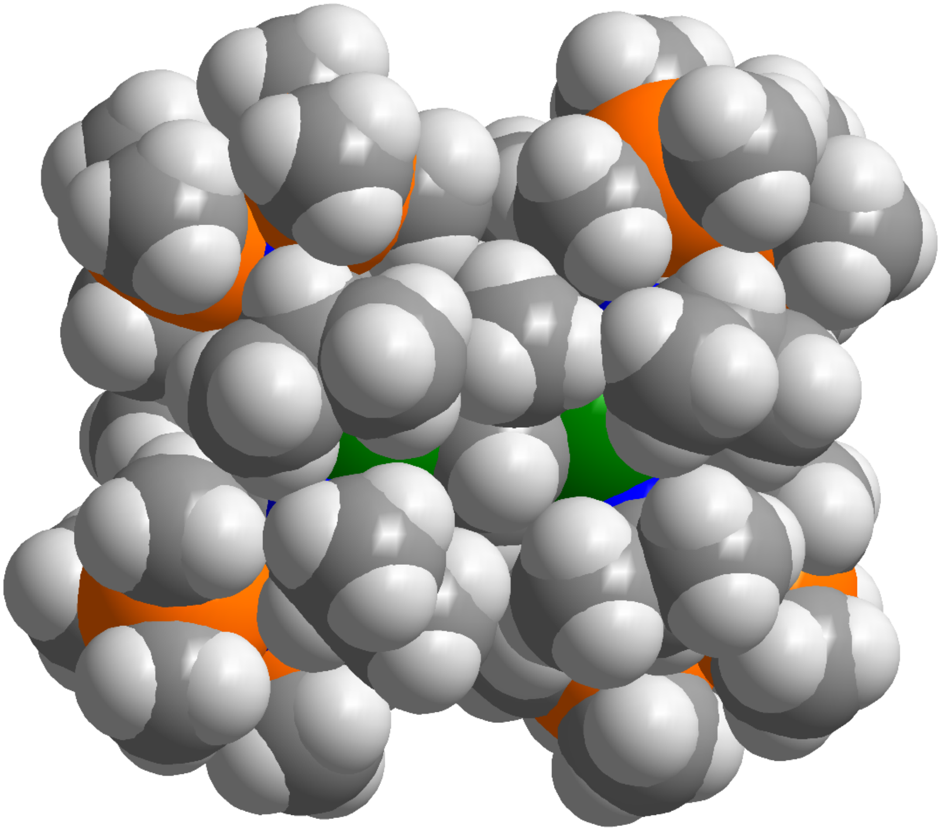

Figure S13. Space-filling model of $[\{(Me_3Si)_2NC(N^iPr)_2\}_2Dy]_2(\mu\text{-}\eta^6{:}\eta^6\text{-}C_6H_5Me)$, **2**. Green, orange, blue, gray, and white-gray spheres represent Dy, Si, N, C, and H atoms, respectively. Only one orientation of the disordered toluene dianion is shown for clarity.

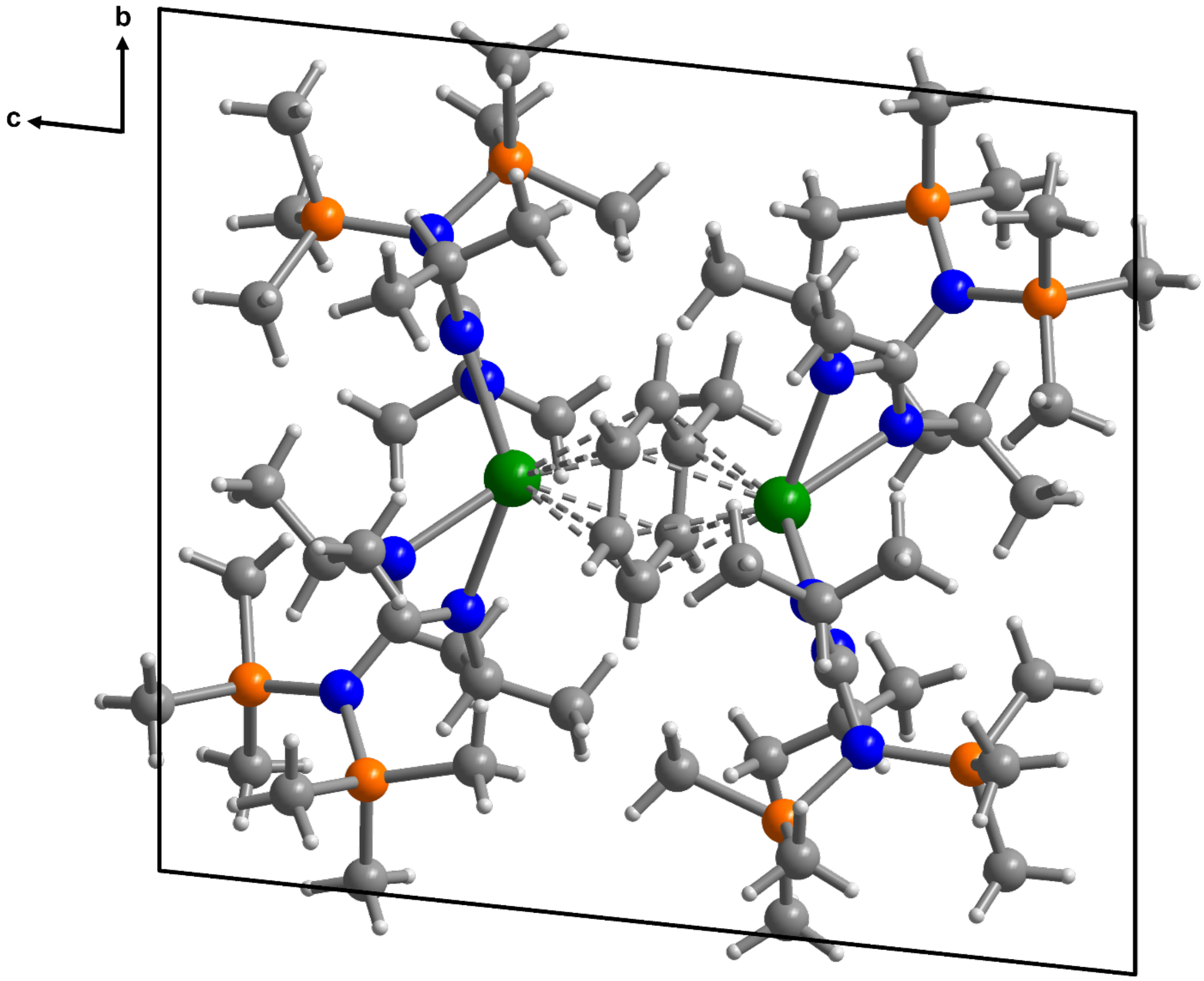

Figure S14. Unit cell of [{(Me$_3$Si)$_2$NC(N^{i}Pr)$_2$}$_2$Dy]$_2$(μ-η^6:η^6-C$_6$H$_5$Me), **2**, along the a-axis. Green, orange, blue, gray, and white-gray spheres represent Dy, Si, N, C, and H atoms, respectively. Only one orientation of the disordered toluene dianion is shown for clarity. Unit cell edges are shown as black lines.

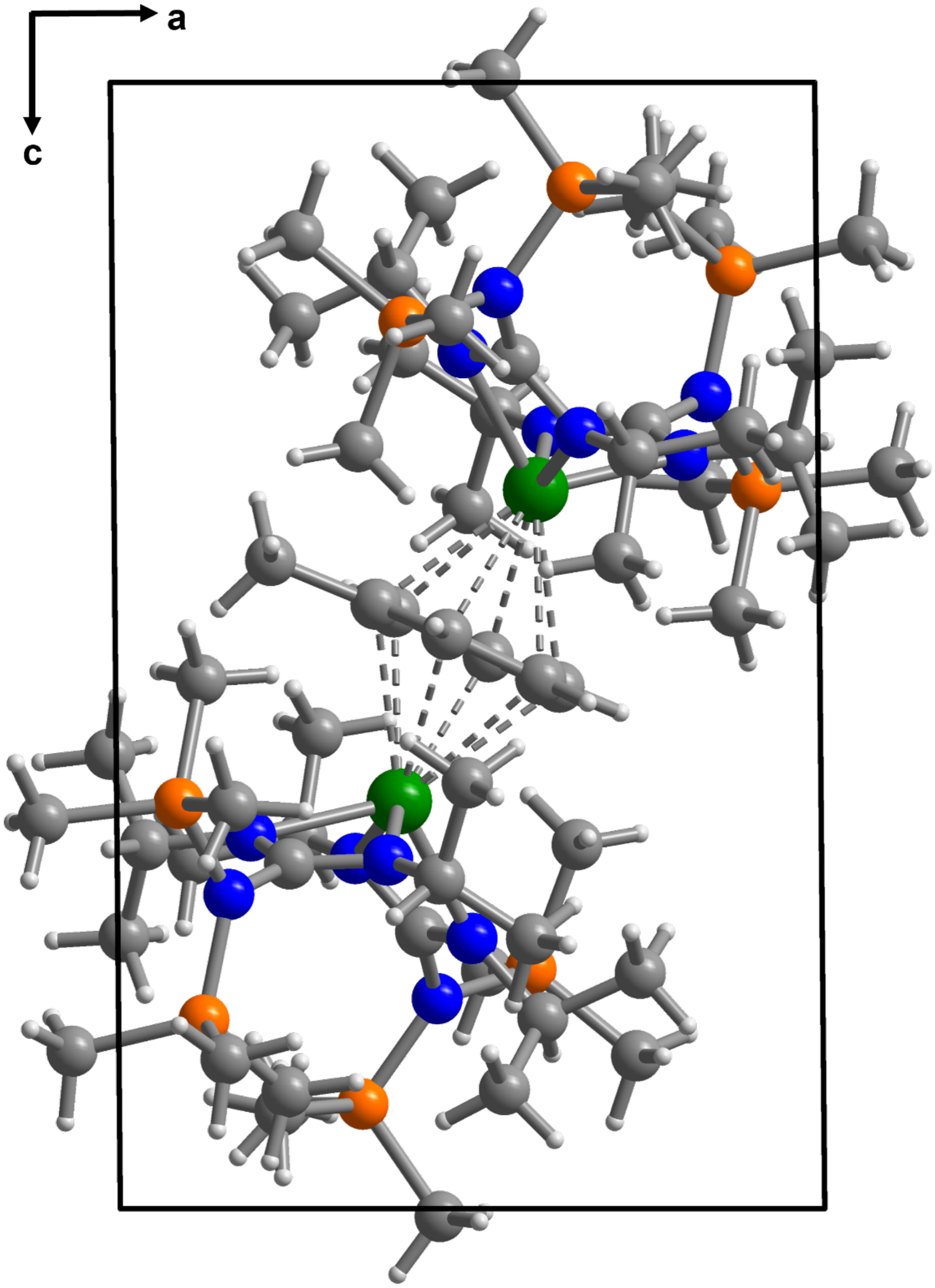

Figure S15. Unit cell of [{$(Me_3Si)_2NC(N^iPr)_2\}_2Dy]_2(\mu$-$\eta^6$:$\eta^6$-$C_6H_5Me$), **2**, along the b-axis. Green, orange, blue, gray, and white-gray spheres represent Dy, Si, N, C, and H atoms, respectively. Only one orientation of the disordered toluene dianion is shown for clarity. Unit cell edges are shown as black lines.

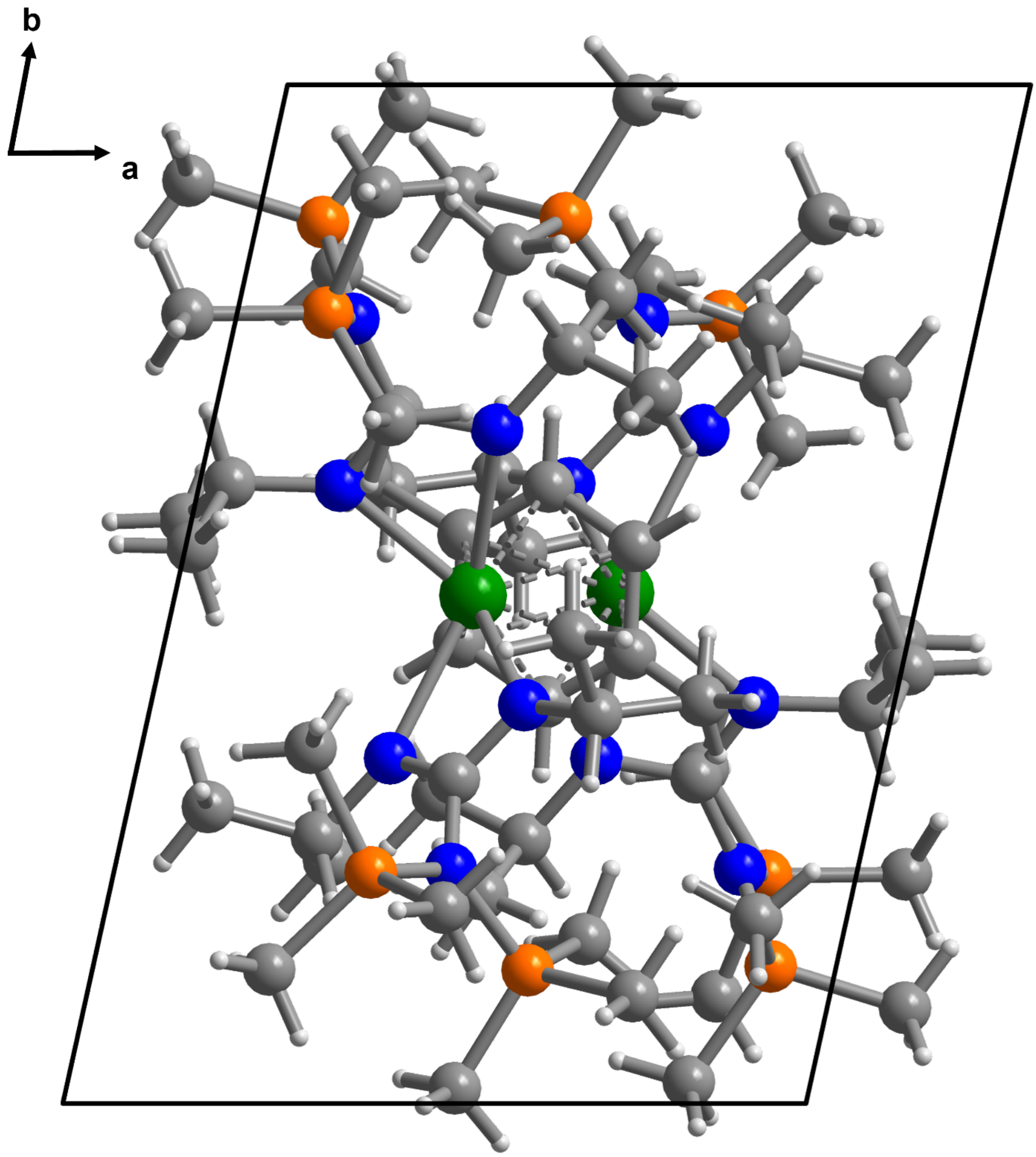

Figure S16. Unit cell of [{$(Me_3Si)_2NC(N^iPr)_2\}_2Dy]_2(\mu\text{-}\eta^6\text{:}\eta^6\text{-}C_6H_5Me)$, **2**, along the c-axis. Green, orange, blue, gray, and white-gray spheres represent Dy, Si, N, C, and H atoms, respectively. Only one orientation of the disordered toluene dianion is shown for clarity. Unit cell edges are shown as black lines.

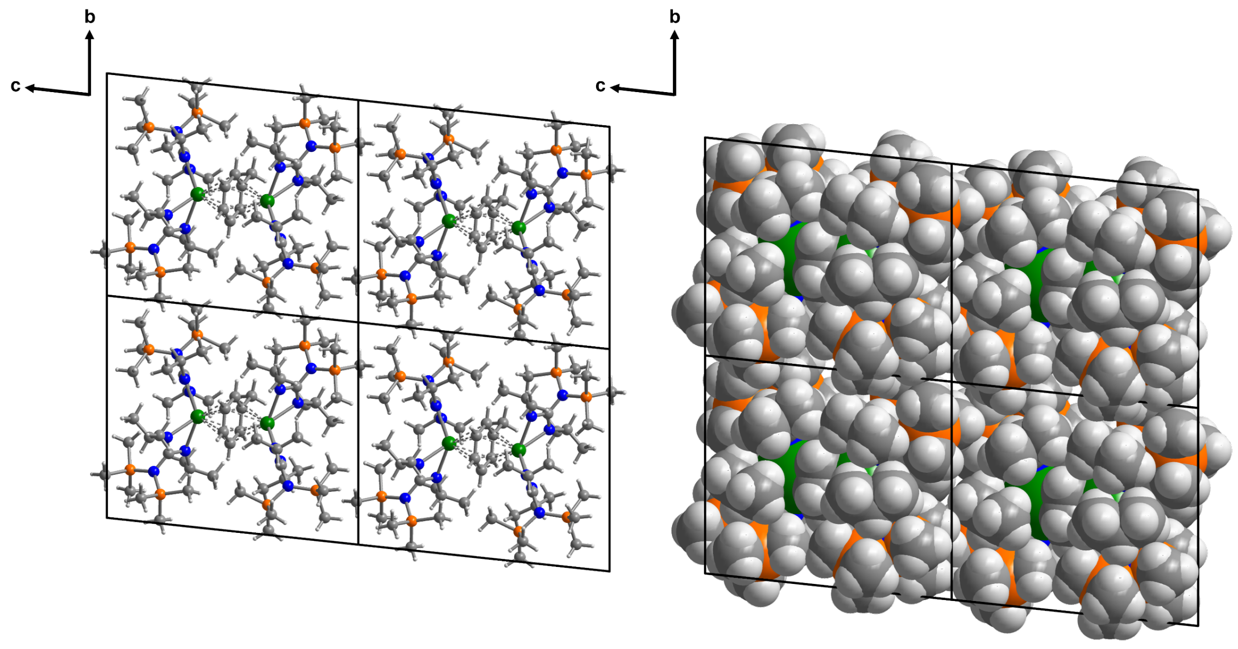

Figure S17. Crystal packing diagram of [{$(Me_3Si)_2NC(N^iPr)_2$}$_2$Dy]$_2$(μ-η^6:η^6-C_6H_5Me), **2**, along the a-axis with ball-and-stick (left) and space-filling (right) representations. Green, orange, blue, gray, and white-gray spheres represent Dy, Si, N, C, and H atoms, respectively. Only one orientation of the disordered toluene dianion is shown for clarity. Unit cell edges are shown as black lines.

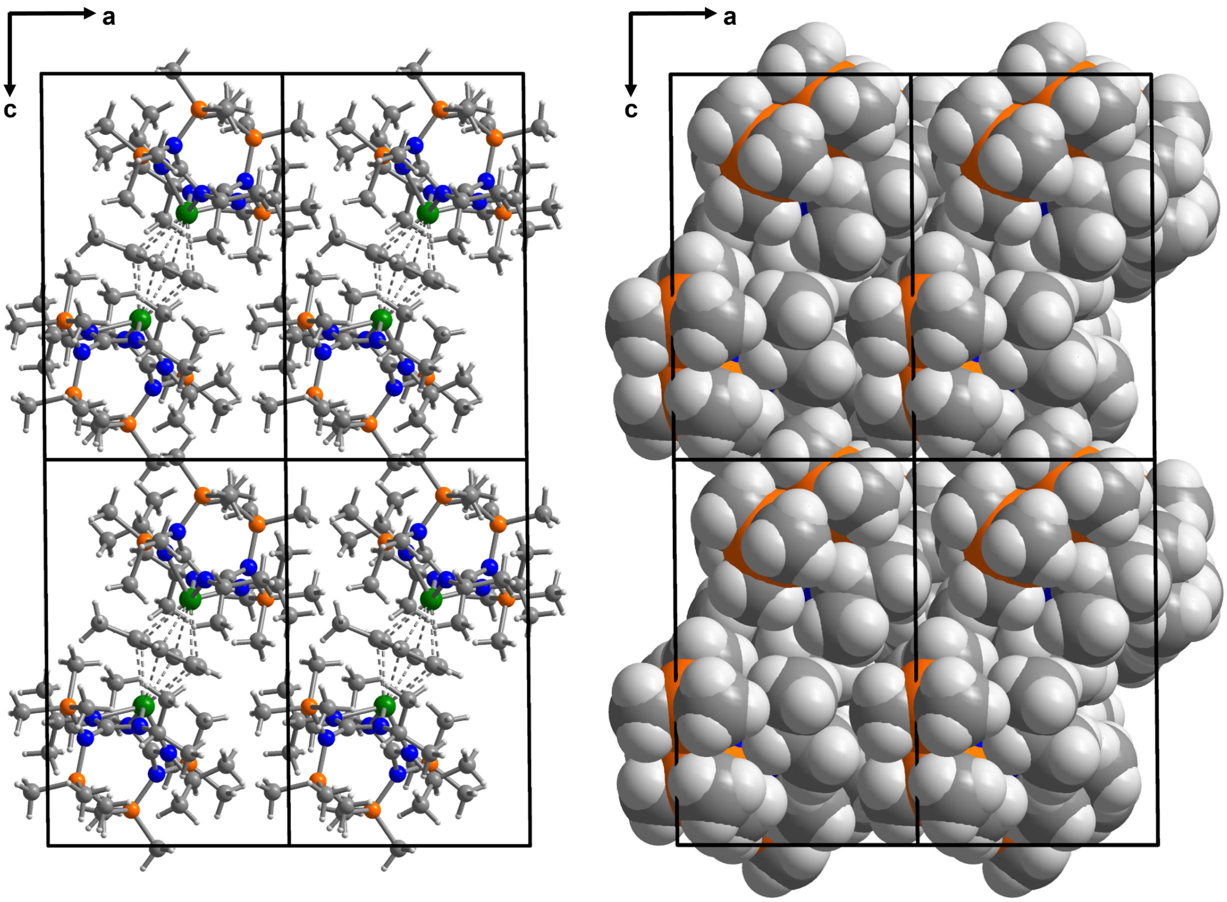

Figure S18. Crystal packing diagram of $[\{(Me_3Si)_2NC(N^iPr)_2\}_2Dy]_2(\mu\text{-}\eta^6\text{:}\eta^6\text{-}C_6H_5Me)$, **2**, along the b-axis with ball-and-stick (left) and space-filling (right) representations. Green, orange, blue, gray, and white-gray spheres represent Dy, Si, N, C, and H atoms, respectively. Only one orientation of the disordered toluene dianion is shown for clarity. Unit cell edges are shown as black lines.

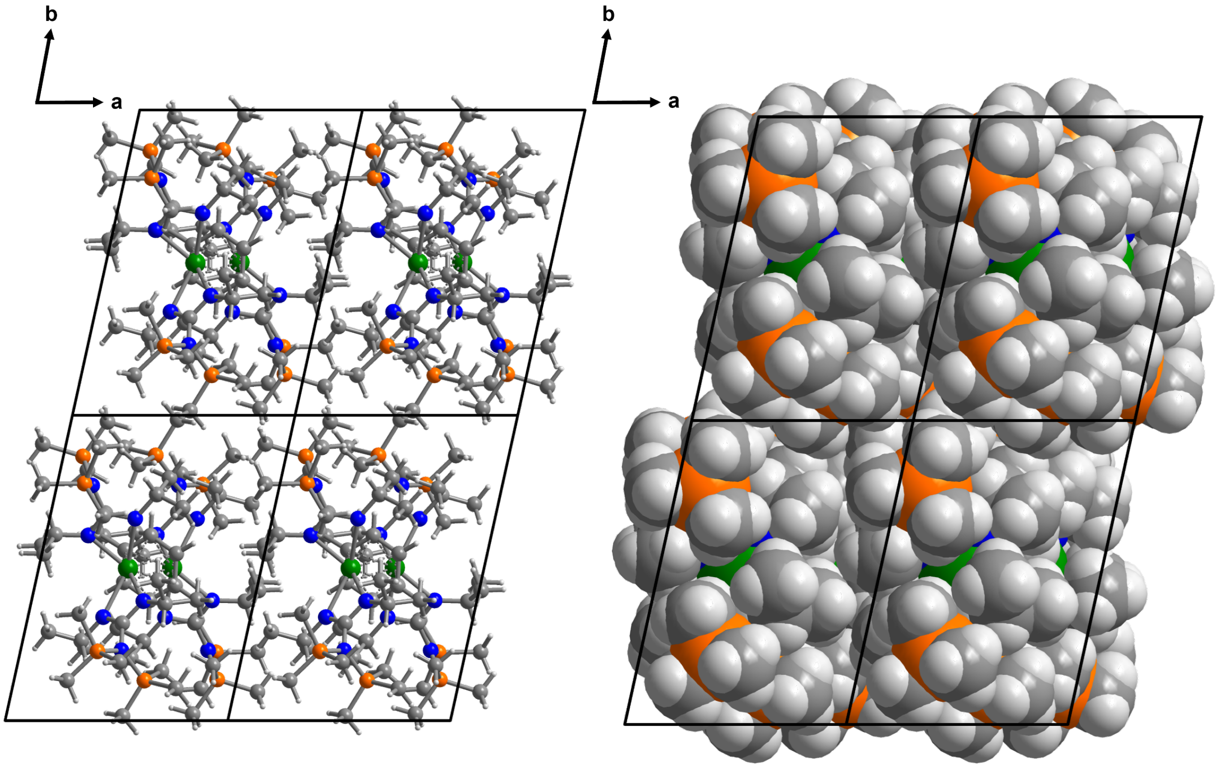

Figure S19. Crystal packing diagram of [{$(Me_3Si)_2NC(N^iPr)_2$}$_2$Dy]$_2$(μ-η^6:η^6-C_6H_5Me), **2**, along the c-axis with ball-and-stick (left) and space-filling (right) representations. Green, orange, blue, gray, and white-gray spheres represent Dy, Si, N, C, and H atoms, respectively. Only one orientation of the disordered toluene dianion is shown for clarity. Unit cell edges are shown as black lines.

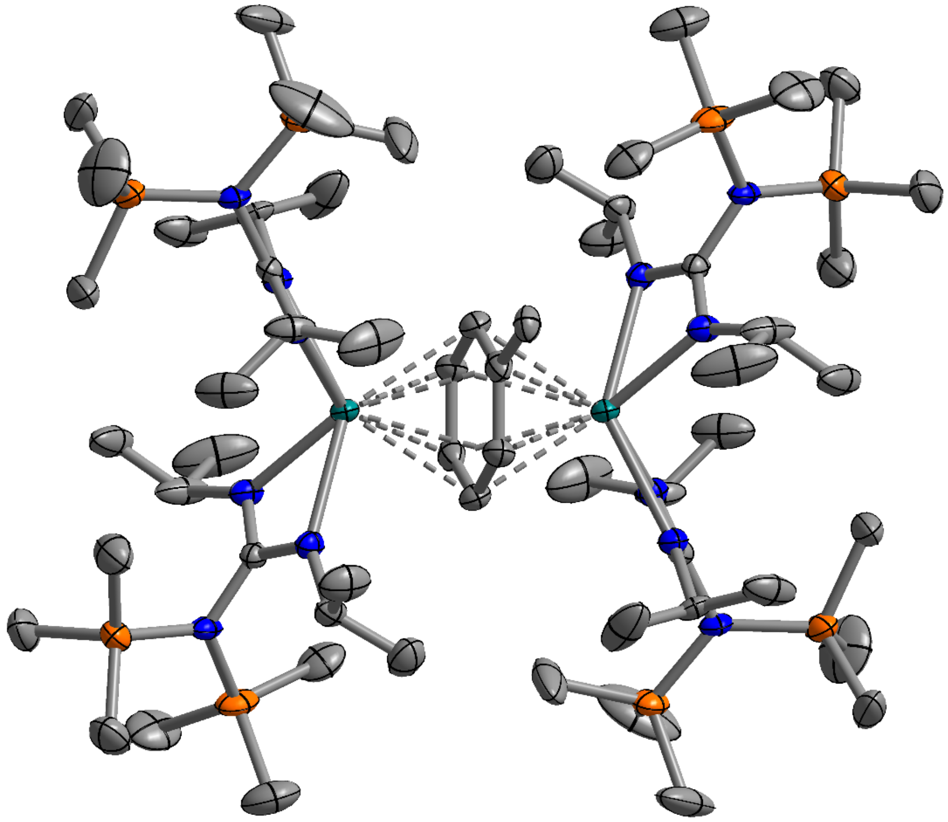

Figure S20. Structure of [{$(Me_3Si)_2NC(N^iPr)_2$}$_2$Er]$_2$(μ-η^6:η^6-C_6H_5Me), **3**, with thermal ellipsoids drawn at the 50% probability level. Teal, orange, blue, and gray spheres represent Er, Si, N, and C atoms, respectively. H atoms have been omitted for clarity. Only one orientation of the disordered toluene dianion is shown for clarity.

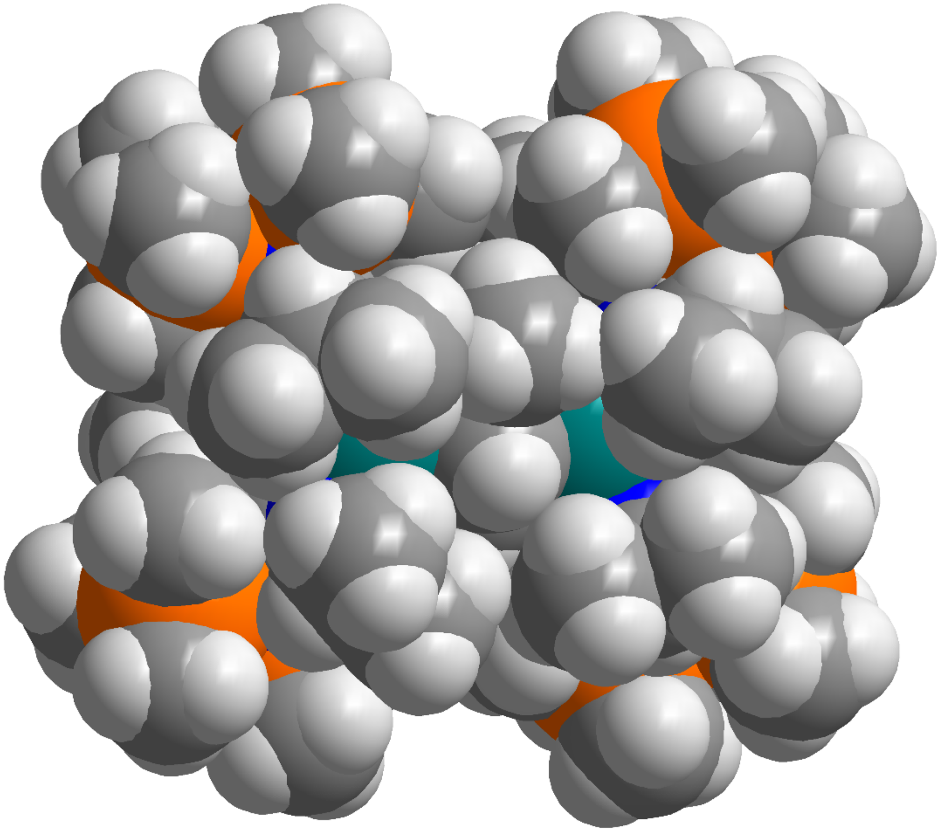

Figure S21. Space-filling model of $[\{(Me_3Si)_2NC(N^iPr)_2\}_2Er]_2(\mu\text{-}\eta^6\text{:}\eta^6\text{-}C_6H_5Me)$, **3**. Teal, orange, blue, gray, and white-gray spheres represent Er, Si, N, C, and H atoms, respectively. Only one orientation of the disordered toluene dianion is shown for clarity.

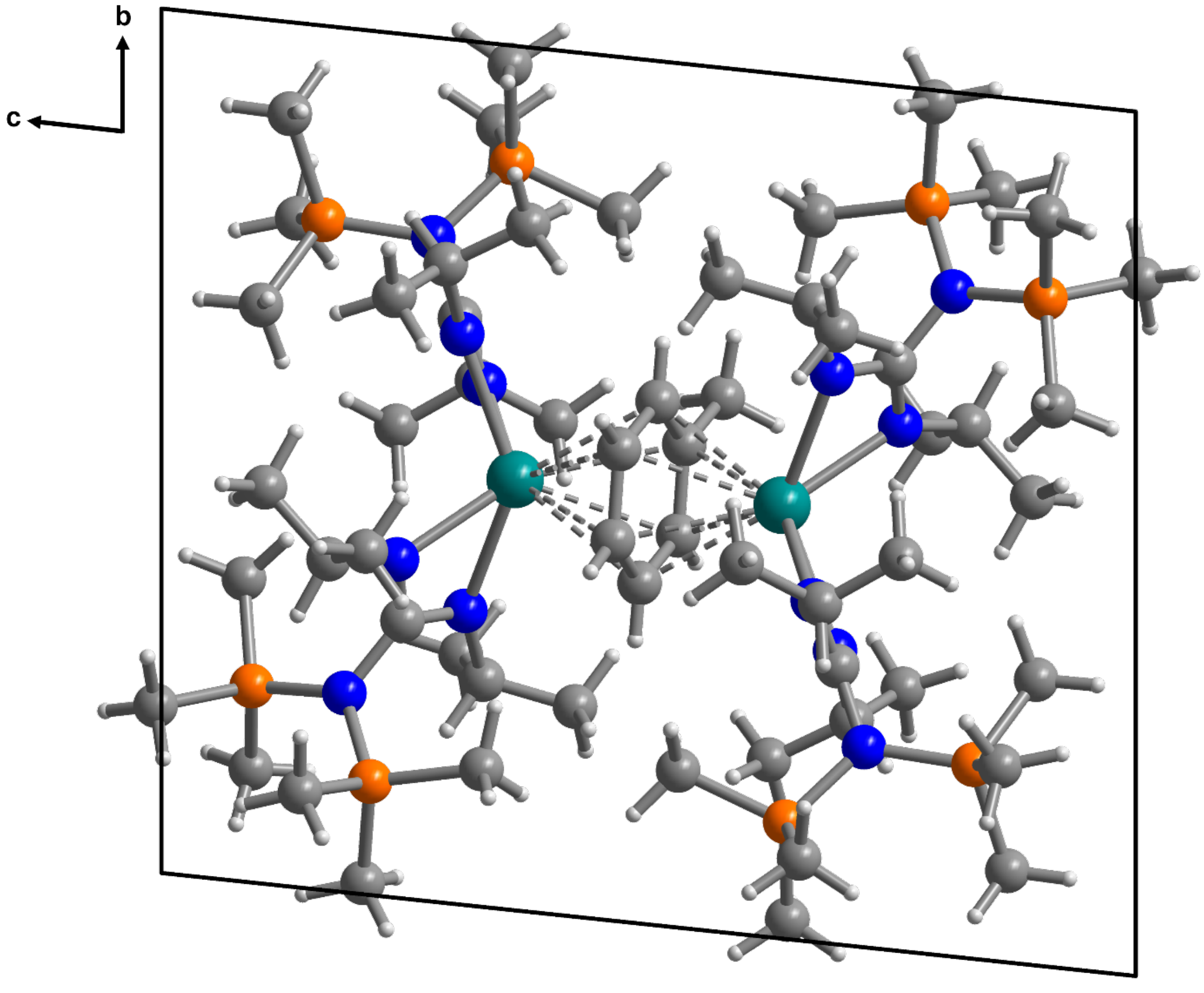

Figure S22. Unit cell of $[\{(Me_3Si)_2NC(N^iPr)_2\}_2Er]_2(\mu\text{-}\eta^6{:}\eta^6\text{-}C_6H_5Me)$, **3**, along the a-axis. Teal, orange, blue, gray, and white-gray spheres represent Er, Si, N, C, and H atoms, respectively. Only one orientation of the disordered toluene dianion is shown for clarity. Unit cell edges are shown as black lines.

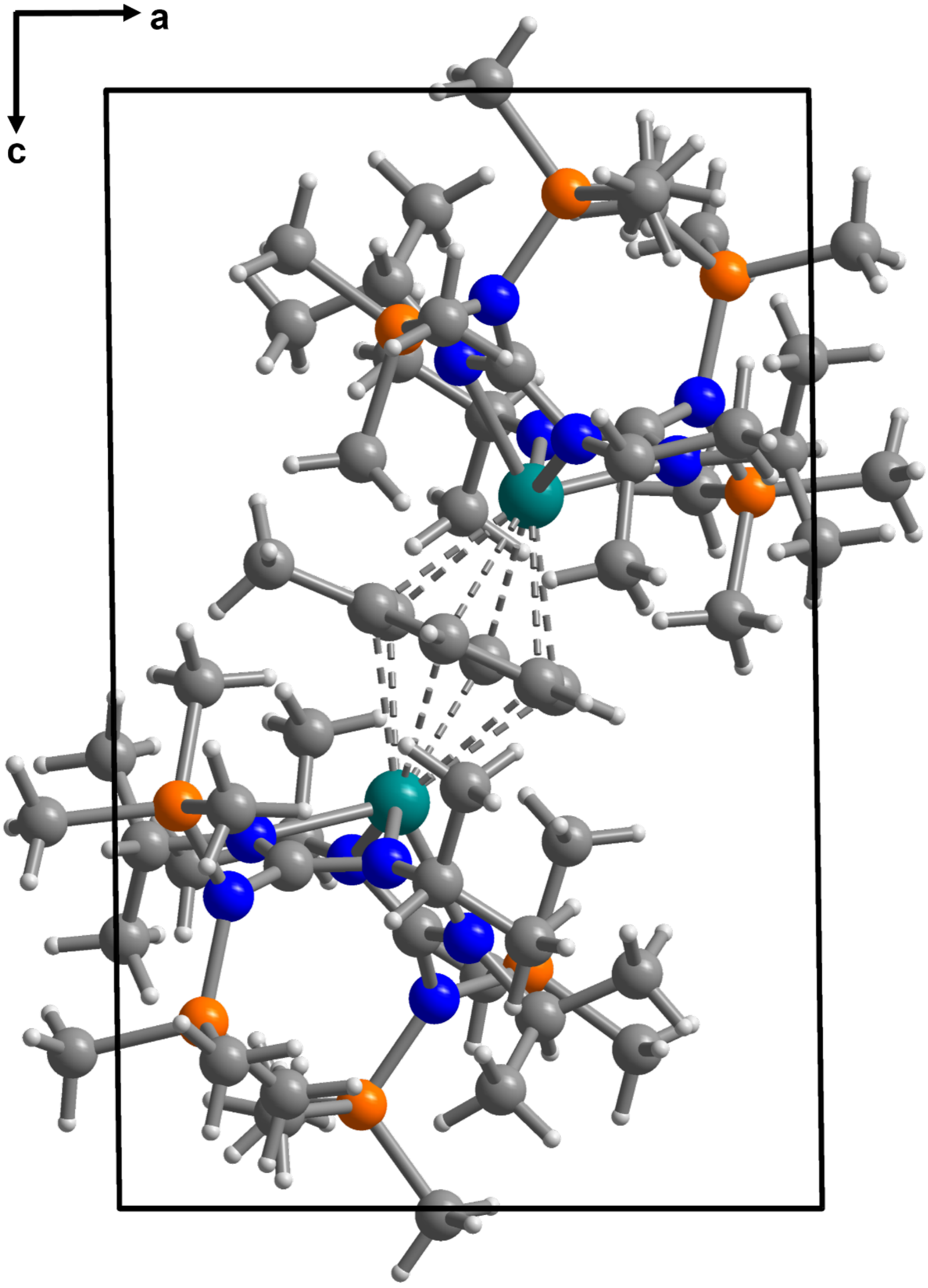

Figure S23. Unit cell of [{$(Me_3Si)_2NC(N^iPr)_2$}$_2Er]_2(\mu-\eta^6:\eta^6-C_6H_5Me)$, **3**, along the b-axis. Teal, orange, blue, gray, and white-gray spheres represent Er, Si, N, C, and H atoms, respectively. Only one orientation of the disordered toluene dianion is shown for clarity. Unit cell edges are shown as black lines.

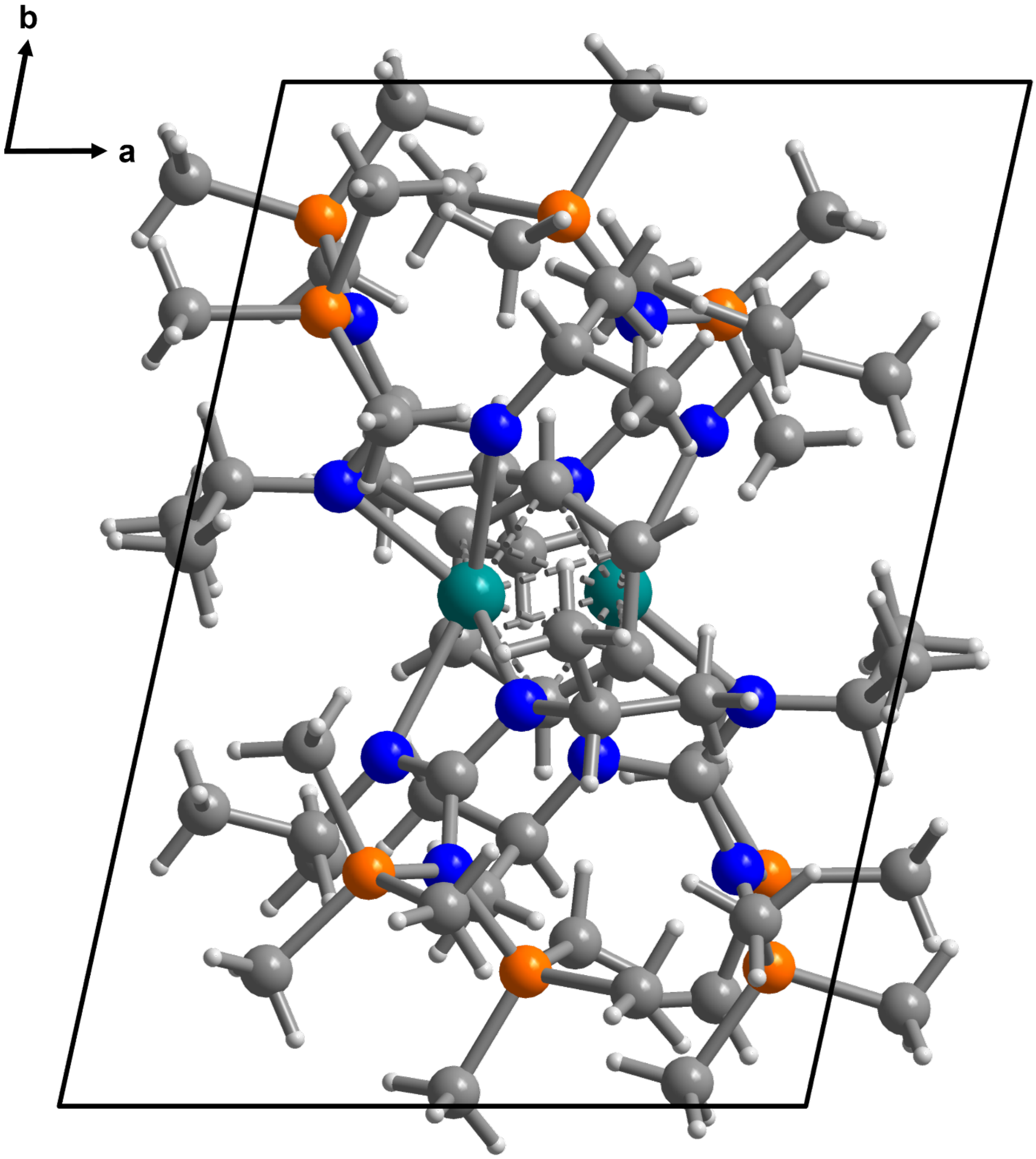

Figure S24. Unit cell of [{$(Me_3Si)_2NC(N^iPr)_2$}$_2Er]_2(\mu-\eta^6:\eta^6-C_6H_5Me)$, **3**, along the c-axis. Teal, orange, blue, gray, and white-gray spheres represent Er, Si, N, C, and H atoms, respectively. Only one orientation of the disordered toluene dianion is shown for clarity. Unit cell edges are shown as black lines.

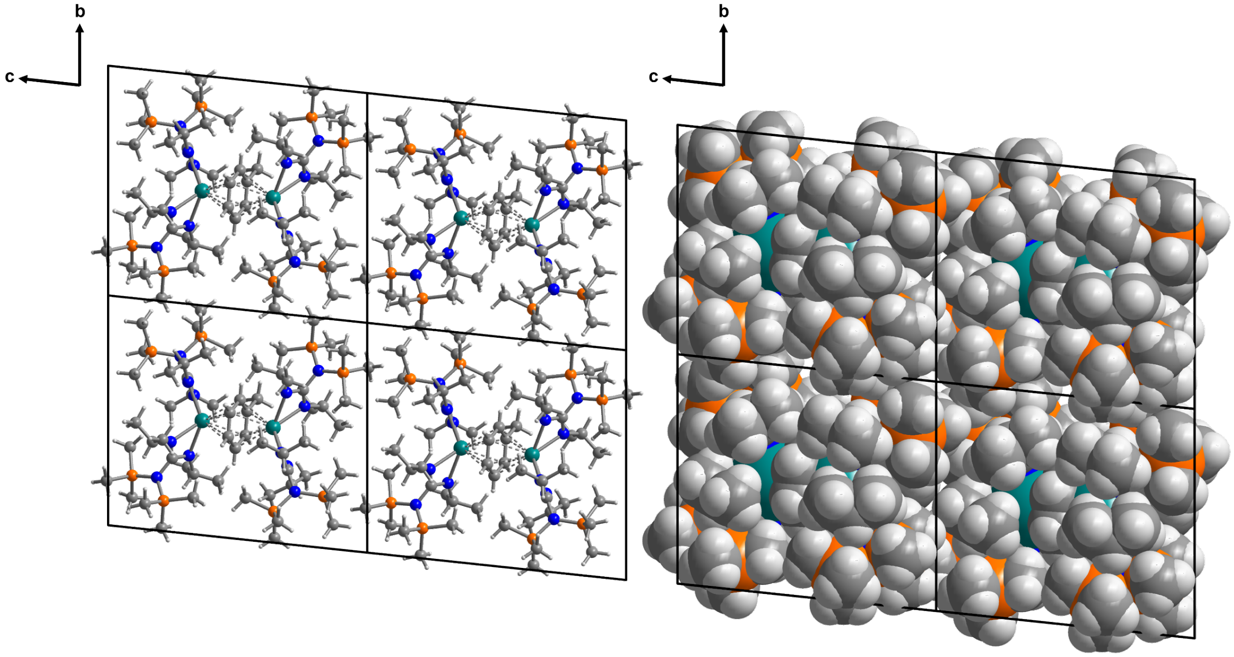

Figure S25. Crystal packing diagram of [{$(Me_3Si)_2NC(N^iPr)_2$}$_2Er]_2(\mu\text{-}\eta^6:\eta^6\text{-}C_6H_5Me)$, **3**, along the a-axis with ball-and-stick (left) and space-filling (right) representations. Teal, orange, blue, gray, and white-gray spheres represent Er, Si, N, C, and H atoms, respectively. Only one orientation of the disordered toluene dianion is shown for clarity. Unit cell edges are shown as black lines.

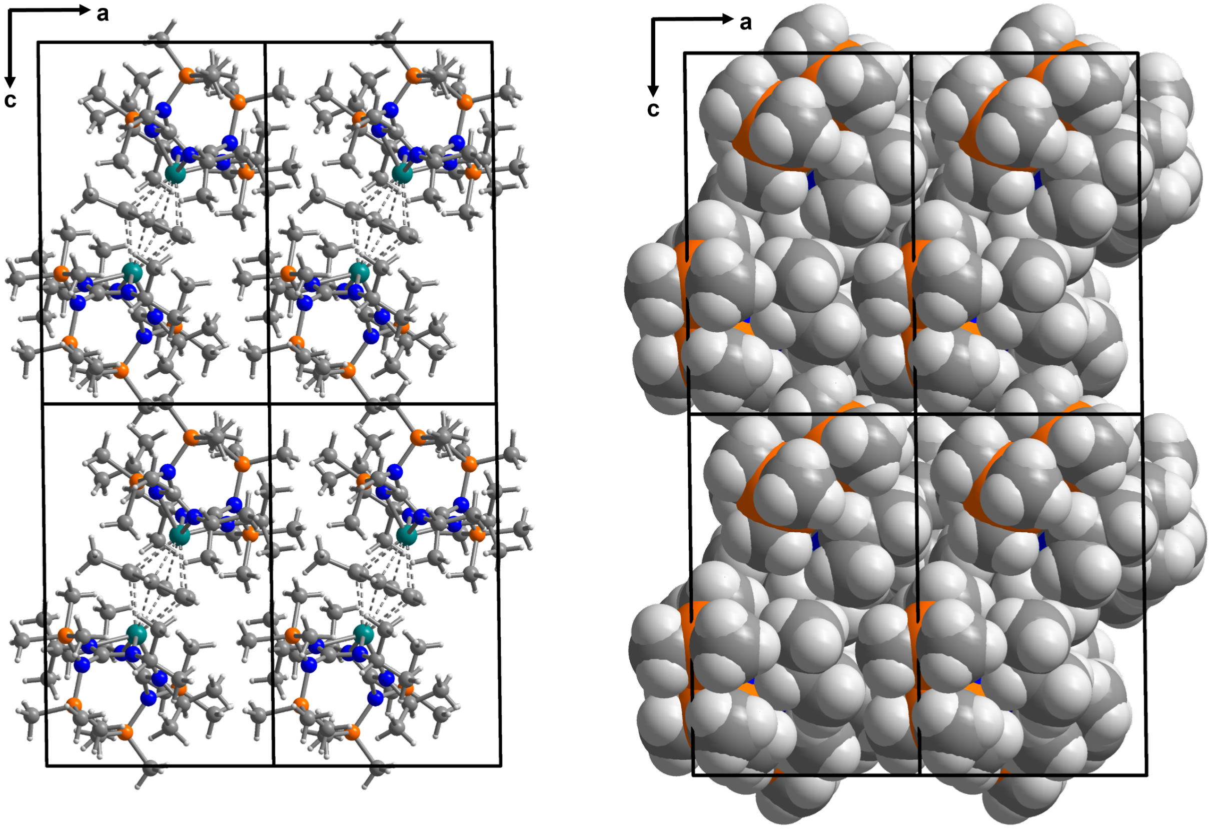

Figure S26. Crystal packing diagram of $[\{(Me_3Si)_2NC(N^iPr)_2\}_2Er]_2(\mu\text{-}\eta^6\text{:}\eta^6\text{-}C_6H_5Me)$, **3**, along the b-axis with ball-and-stick (left) and space-filling (right) representations. Teal, orange, blue, gray, and white-gray spheres represent Er, Si, N, C, and H atoms, respectively. Only one orientation of the disordered toluene dianion is shown for clarity. Unit cell edges are shown as black lines.

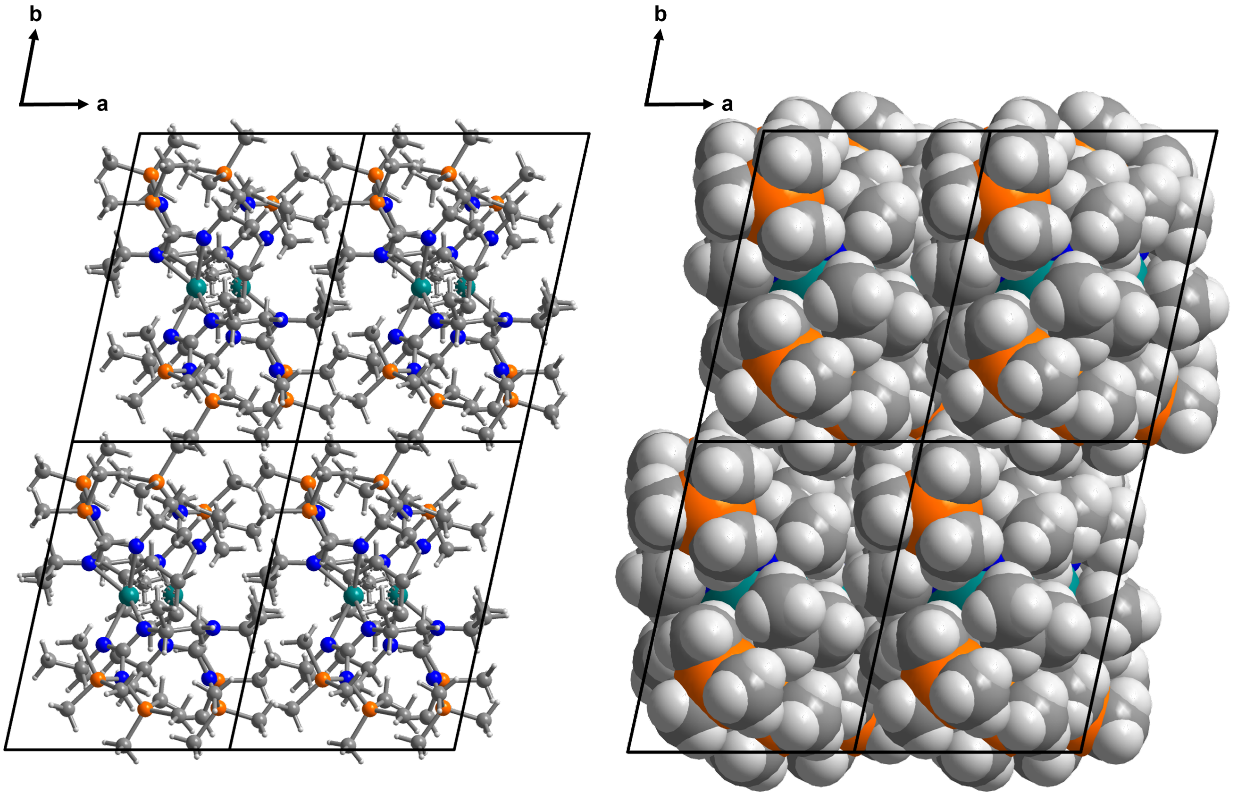

Figure S27. Crystal packing diagram of $[\{(Me_3Si)_2NC(N^iPr)_2\}_2Er]_2(\mu\text{-}\eta^6\text{:}\eta^6\text{-}C_6H_5Me)$, **3**, along the c-axis with ball-and-stick (left) and space-filling (right) representations. Teal, orange, blue, gray, and white-gray spheres represent Er, Si, N, C, and H atoms, respectively. Only one orientation of the disordered toluene dianion is shown for clarity. Unit cell edges are shown as black lines.

2 NMR Spectroscopy

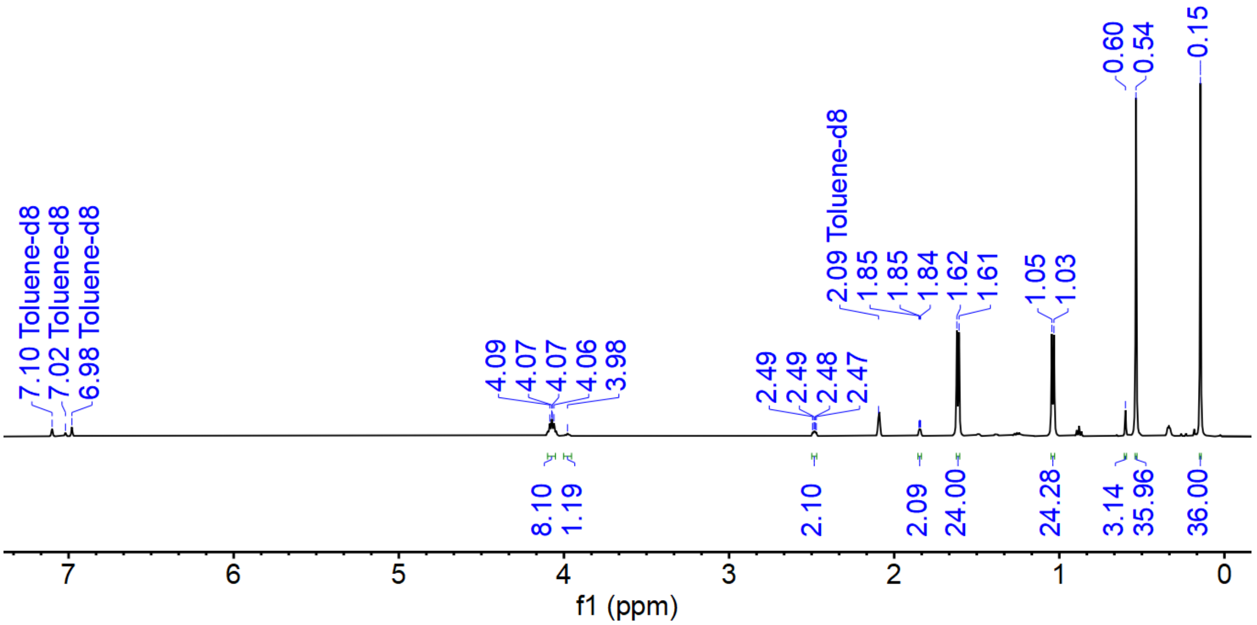

Figure S28. ^{1}H NMR spectrum of [{(Me$_3$Si)$_2$NC(N^{i}Pr)$_2$}$_2$Y]$_2$(μ-η^6:η^6-C$_6$H$_5$Me), **1,** (500 MHz, ppm, toluene-*d*$_8$, 25 °C): *δ* 0.15, 0.54 (s, 72 H, Si(C*H*$_3$)$_3$), 0.60 (s, 3 H, C*H*$_3$C$_6$H$_5$), 1.03, 1.61 (d, $^3J_{H–H}$ = 6.34, 6.47f Hz, 48 H, CH(C*H*$_3$)$_2$), 1.84 (t, $^3J_{H–H}$ = 2.67 Hz, 3 H, CH$_3$C$_6$*H*$_5$), 2.48 (q, $^3J_{H–H}$ = 4.05 Hz, 2 H, CH$_3$C$_5$*H*$_5$), 3.98 (t, $^3J_{H–H}$ = 7.32 Hz, 1 H, CH$_3$C$_6$*H*$_5$), 4.07 (m, $^3J_{H–H}$ = 6.27 Hz, 8 H, C*H*(CH$_3$)$_2$).

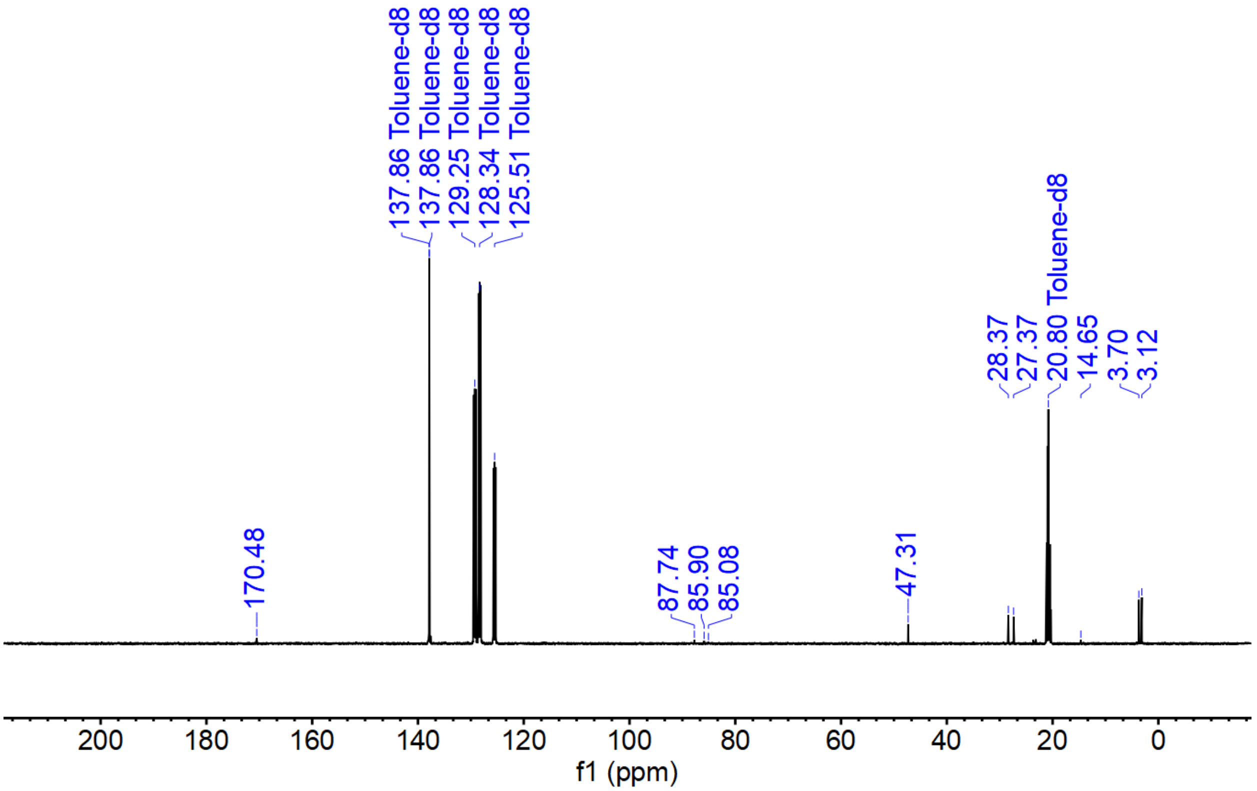

Figure S29. $^{13}C\{H\}$ NMR spectrum of $[\{(Me_3Si)_2NC(N^iPr)_2\}_2Y]_2(\mu\text{-}\eta^6\text{:}\eta^6\text{-}C_6H_5Me)$, **1,** (126 MHz, ppm, toluene-d_8, 25 °C): δ 170.48 ($\underline{C}N_3$), 87.74, 85.90, 85.08 ($CH_3\underline{C}_6H_5$), 47.31 ($\underline{C}H(CH_3)_2$), 28.37, 27.37 ($CH(\underline{C}H_3)_2$), 14.65 ($\underline{C}H_3C_6H_5$), 3.70, 3.12 ($Si(\underline{C}H_3)_3$).

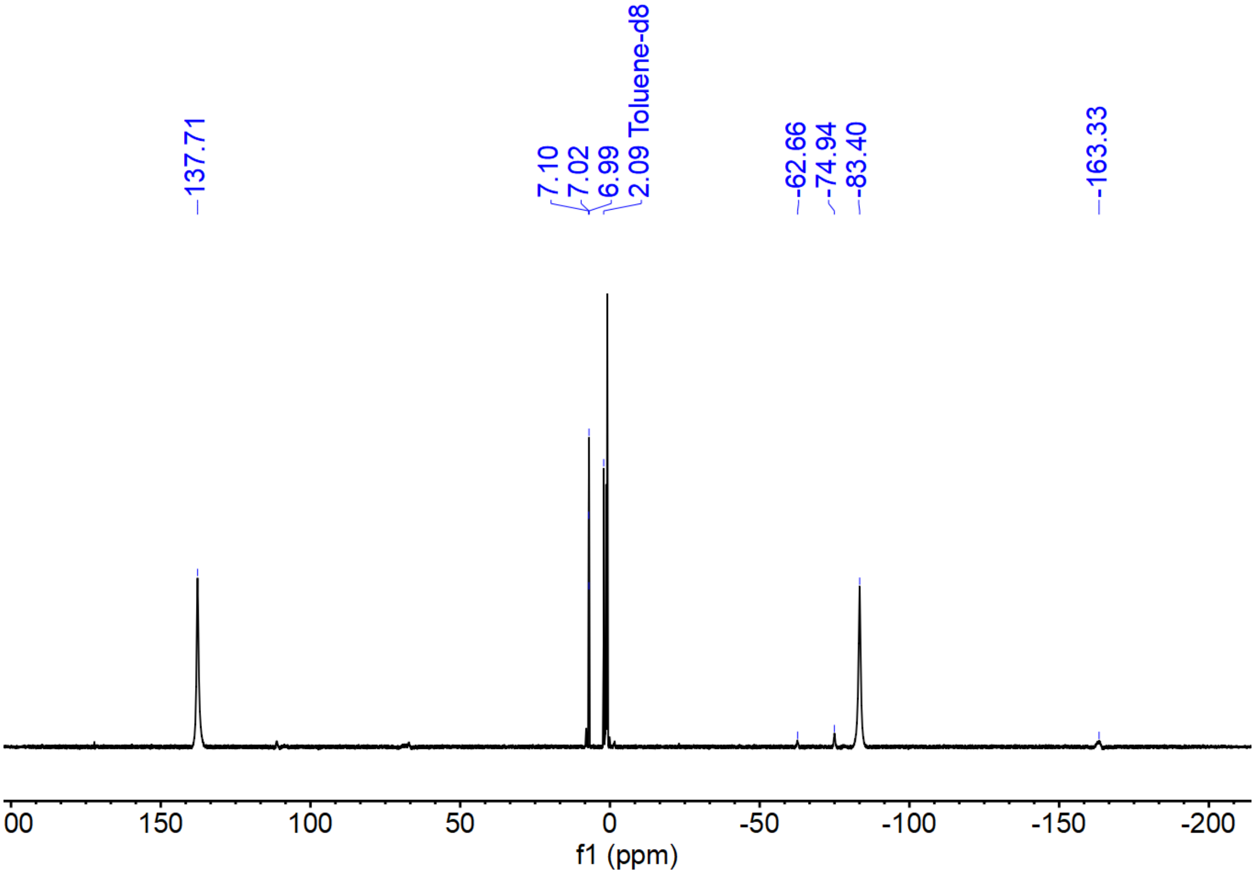

Figure S30. ^{1}H NMR spectrum of $[\{(Me_3Si)_2NC(N^iPr)_2\}_2Dy]_2(\mu\text{-}\eta^6\text{:}\eta^6\text{-}C_6H_5Me)$, **2,** (500 MHz, ppm, toluene-d_8, 25 °C): δ –163.33, –83.40, –74.94, –62.66, 6.99, 7.02, 7.10, 137.

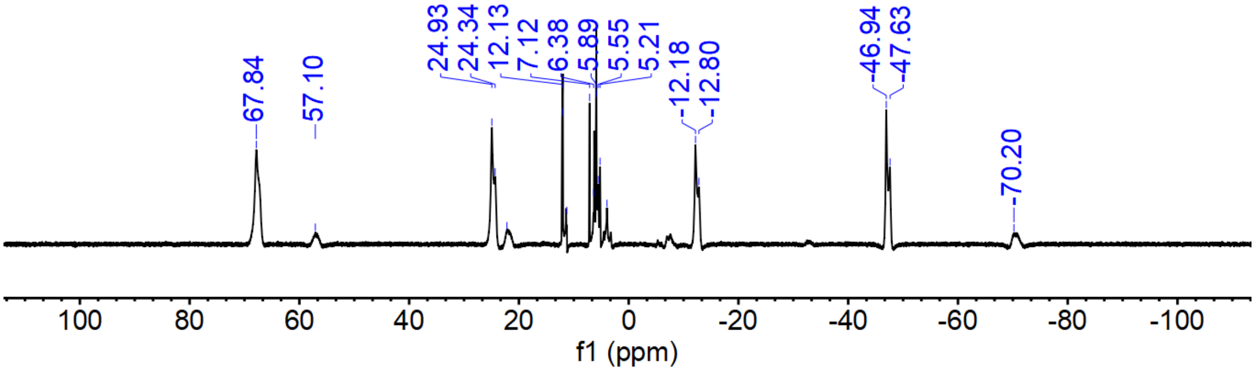

Figure S31. ^{1}H NMR spectrum of $[\{(Me_3Si)_2NC(N^iPr)_2\}_2Er]_2(\mu\text{-}\eta^6\text{:}\eta^6\text{-}C_6H_5Me)$, **3**, (500 MHz, ppm, toluene-d_8, 25 °C): δ –70.20, –47.63, –46.94, –12.80, –12.18, 5.21, 5.55, 5.89, 6.39, 7.12, 12.13, 24.34, 14.93, 57.10, 67.84.

3 IR Spectroscopy

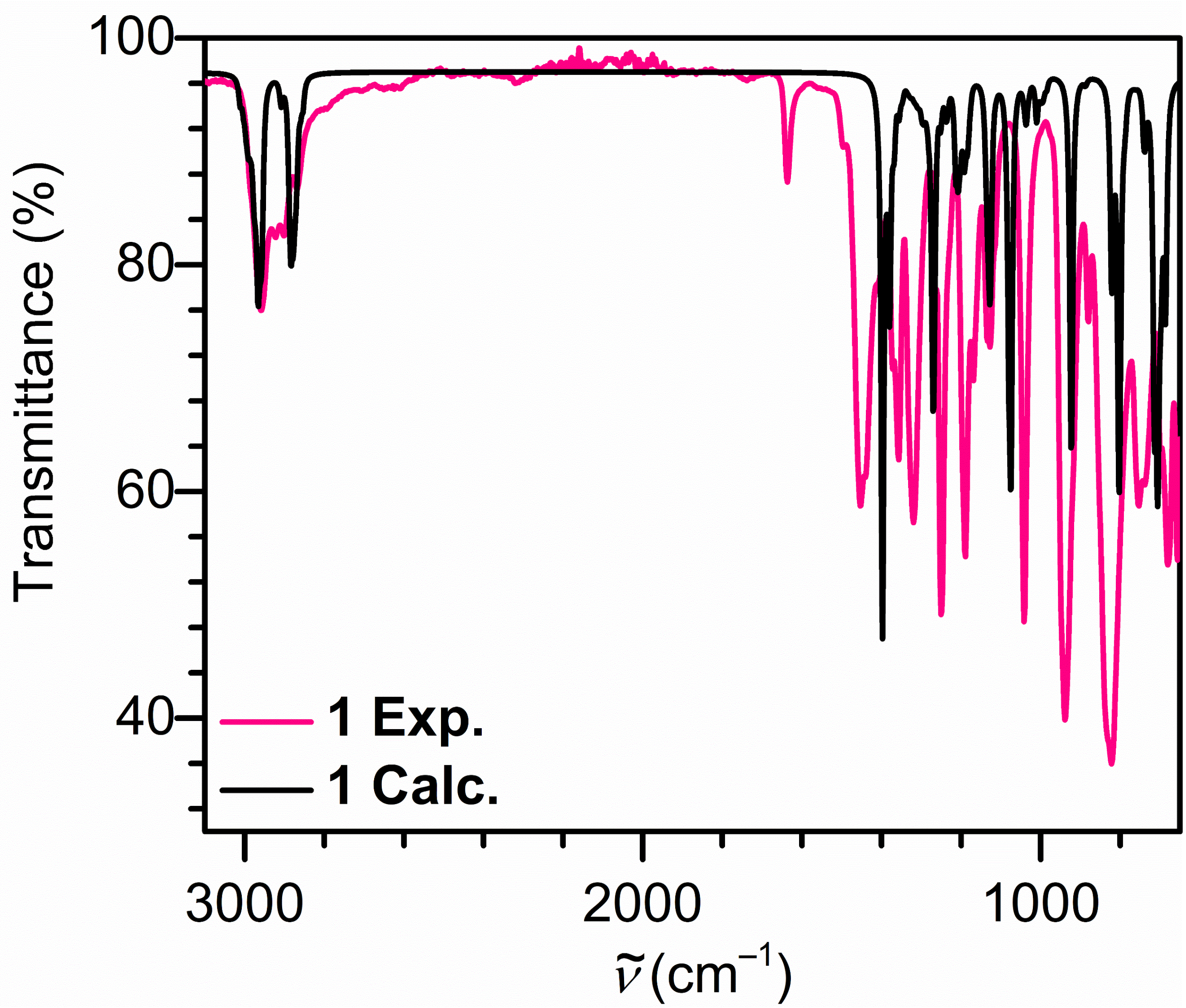

Figure S32. Experimental FTIR spectrum of $[\{(Me_3Si)_2NC(N^iPr)_2\}_2Y]_2(\mu\text{-}\eta^6\text{:}\eta^6\text{-}C_6H_5Me)$, **1**, (pink trace) measured on crushed crystalline solids under a nitrogen atmosphere. DFT-calculated frequencies are plotted in black. The predicted vibrational modes were shifted by 154 cm^{-1}.

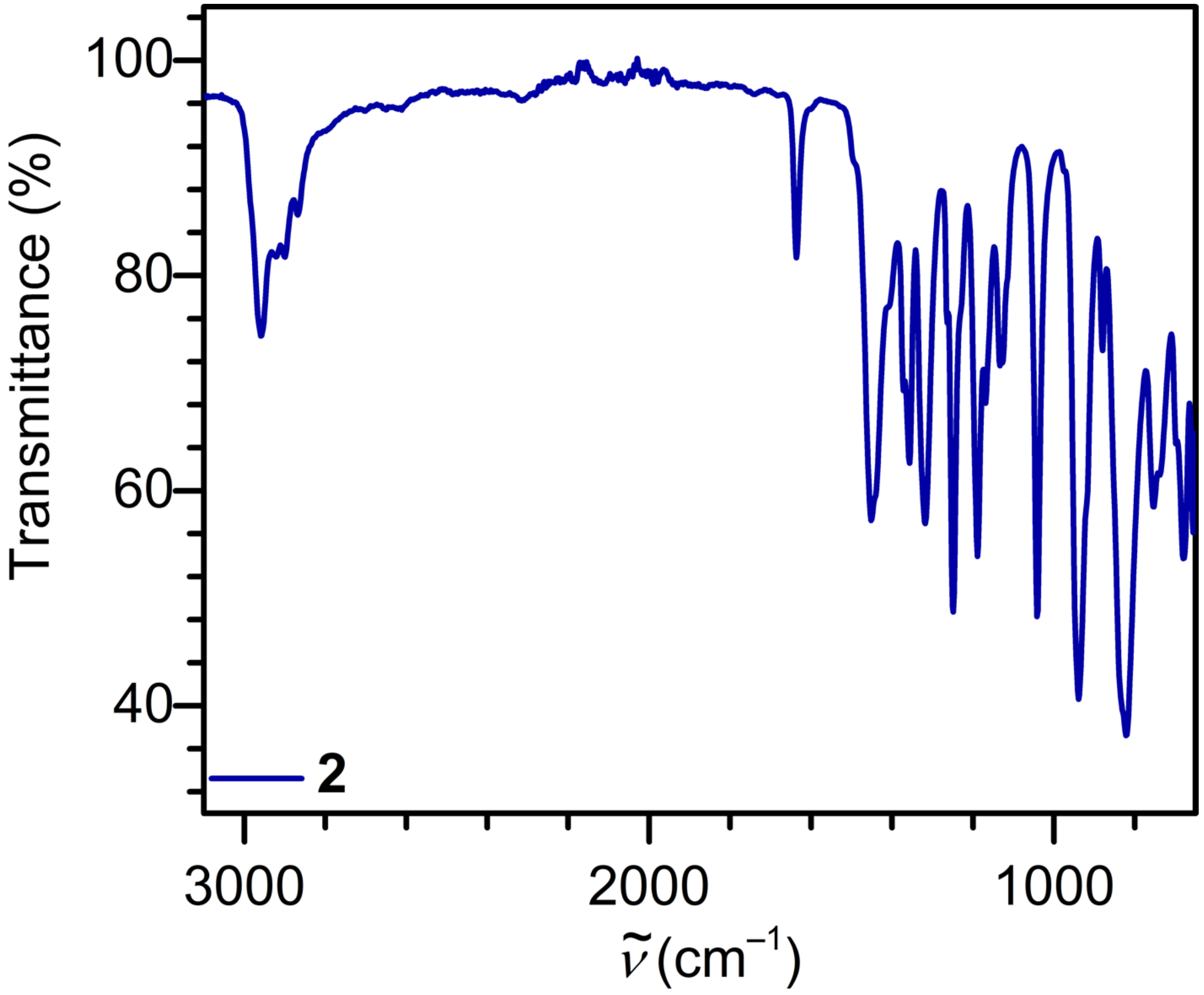

Figure S33. Experimental FTIR spectrum of $[\{(Me_3Si)_2NC(N^iPr)_2\}_2Dy]_2(\mu\text{-}\eta^6\text{:}\eta^6\text{-}C_6H_5Me)$, **2**, (blue trace) measured on crushed crystalline solids under a nitrogen atmosphere.

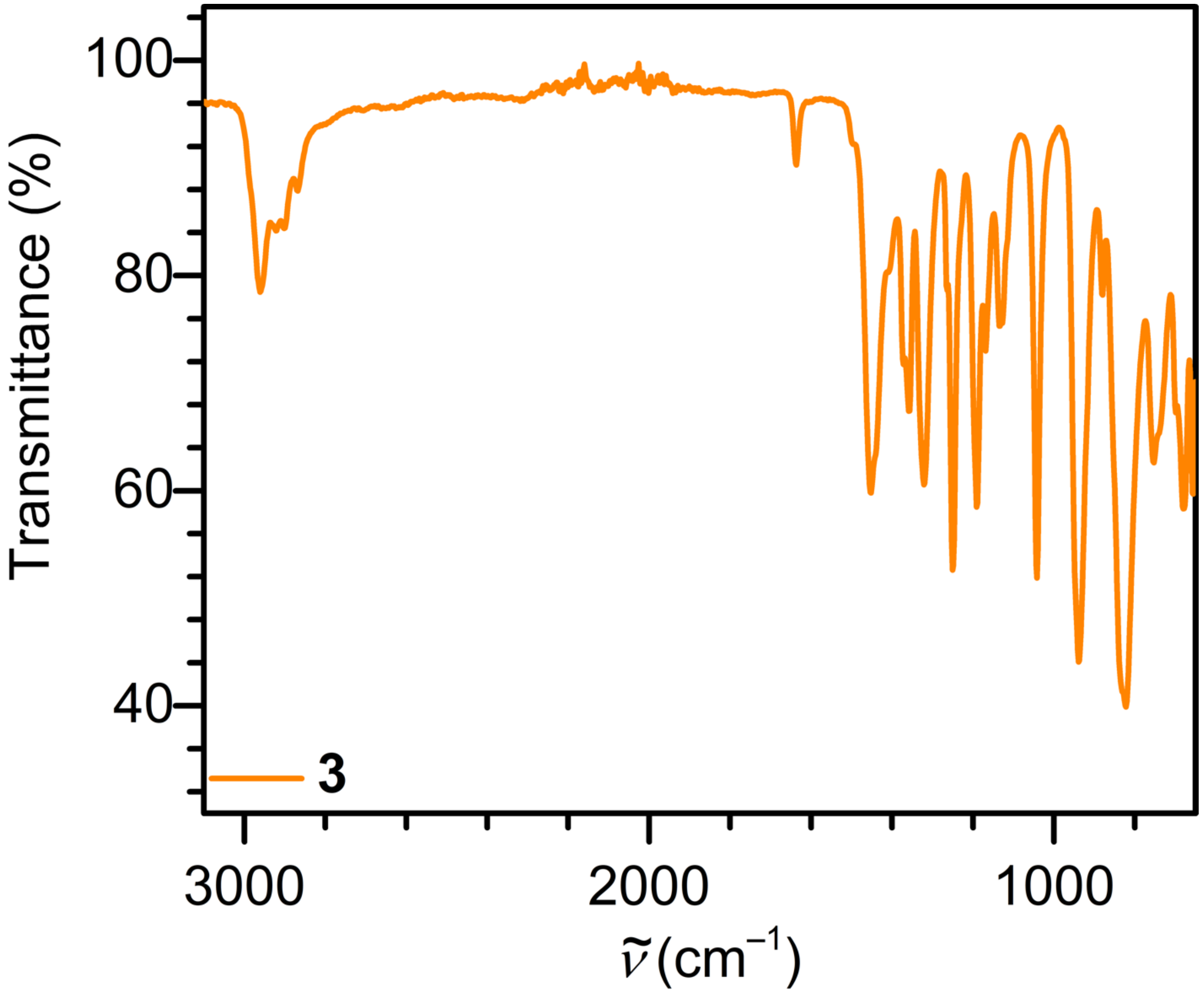

Figure S34. Experimental FTIR spectrum of $[\{(Me_3Si)_2NC(N^iPr)_2\}_2Er]_2(\mu\text{-}\eta^6\text{:}\eta^6\text{-}C_6H_5Me)$, **3**, (orange trace) measured on crushed crystalline solids under a nitrogen atmosphere.

4 UV–Vis Spectroscopy

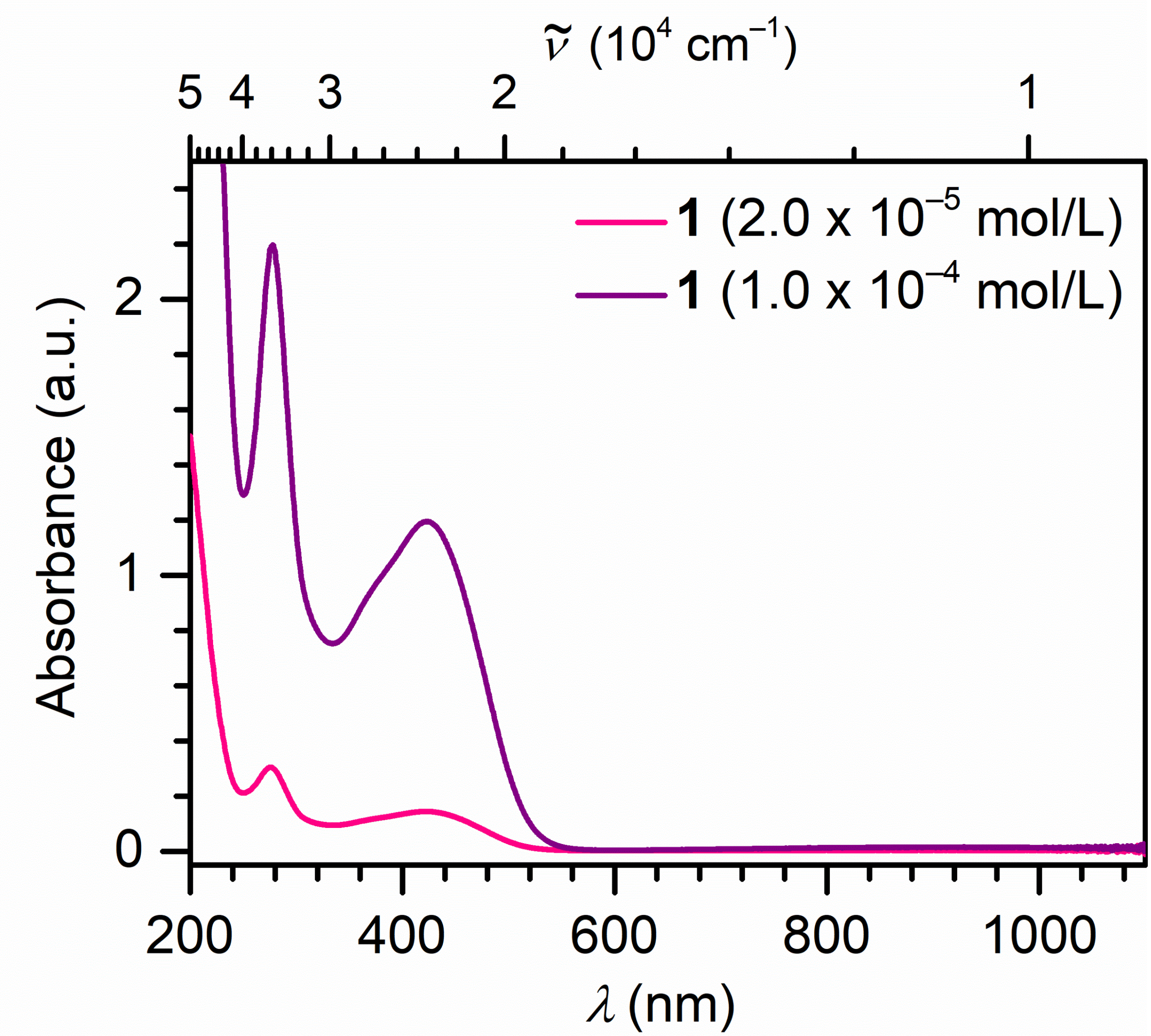

Figure S35. UV-vis spectra of $[\{(Me_3Si)_2NC(N^iPr)_2\}_2Y]_2(\mu\text{-}\eta^6\text{:}\eta^6\text{-}C_6H_5Me)$, **1**, recorded at 100 μmol/L (purple trace) and 20 μmol/L (pink trace) concentrations in ^{n}hexane at room temperature.

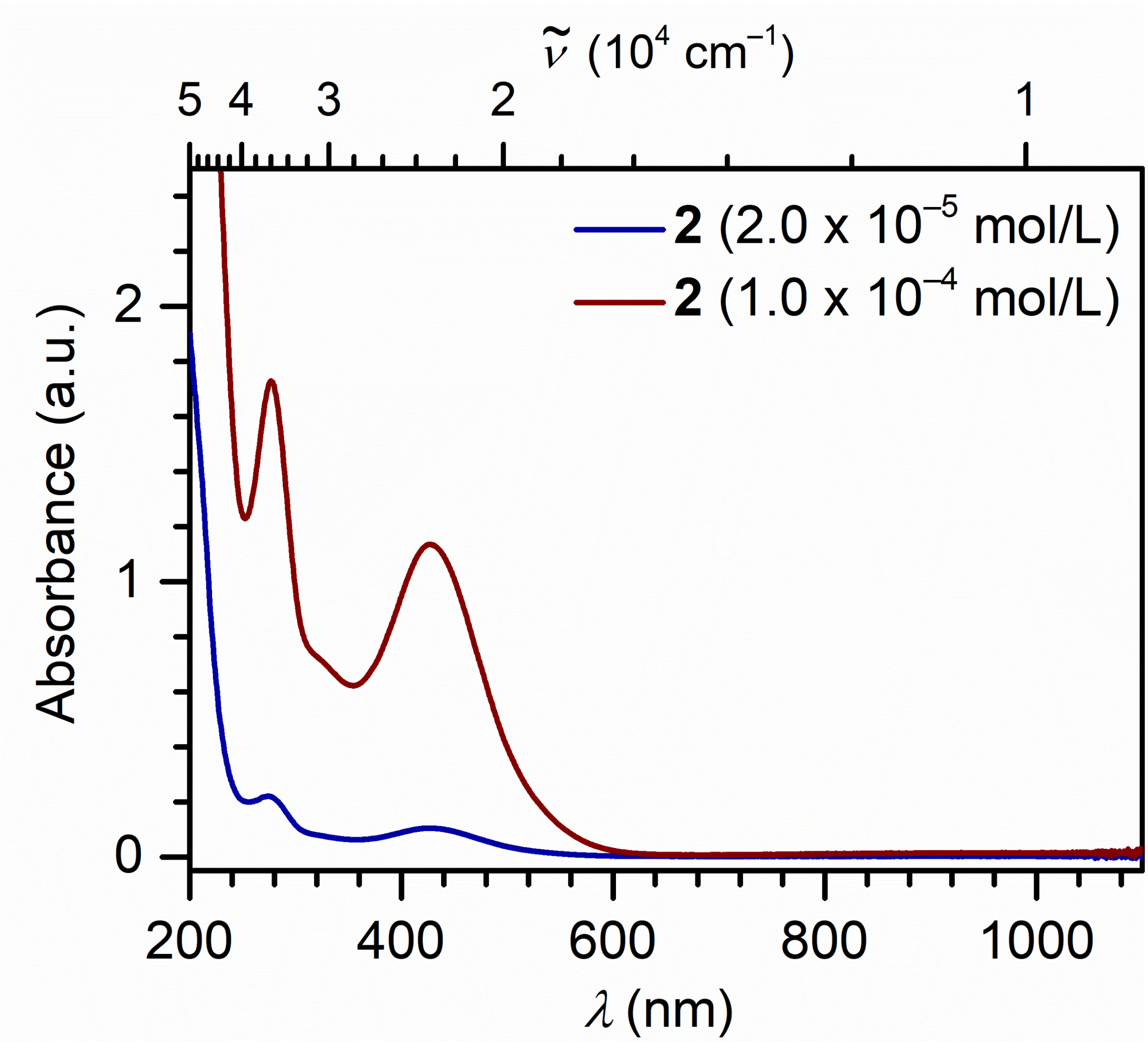

Figure S36. UV-vis spectra of $[\{(Me_3Si)_2NC(N^iPr)_2\}_2Dy]_2(\mu\text{-}\eta^6{:}\eta^6\text{-}C_6H_5Me)$, **2**, recorded at 100 μmol/L (maroon trace) and 20 μmol/L (blue trace) concentrations in ^{n}hexane at room temperature.

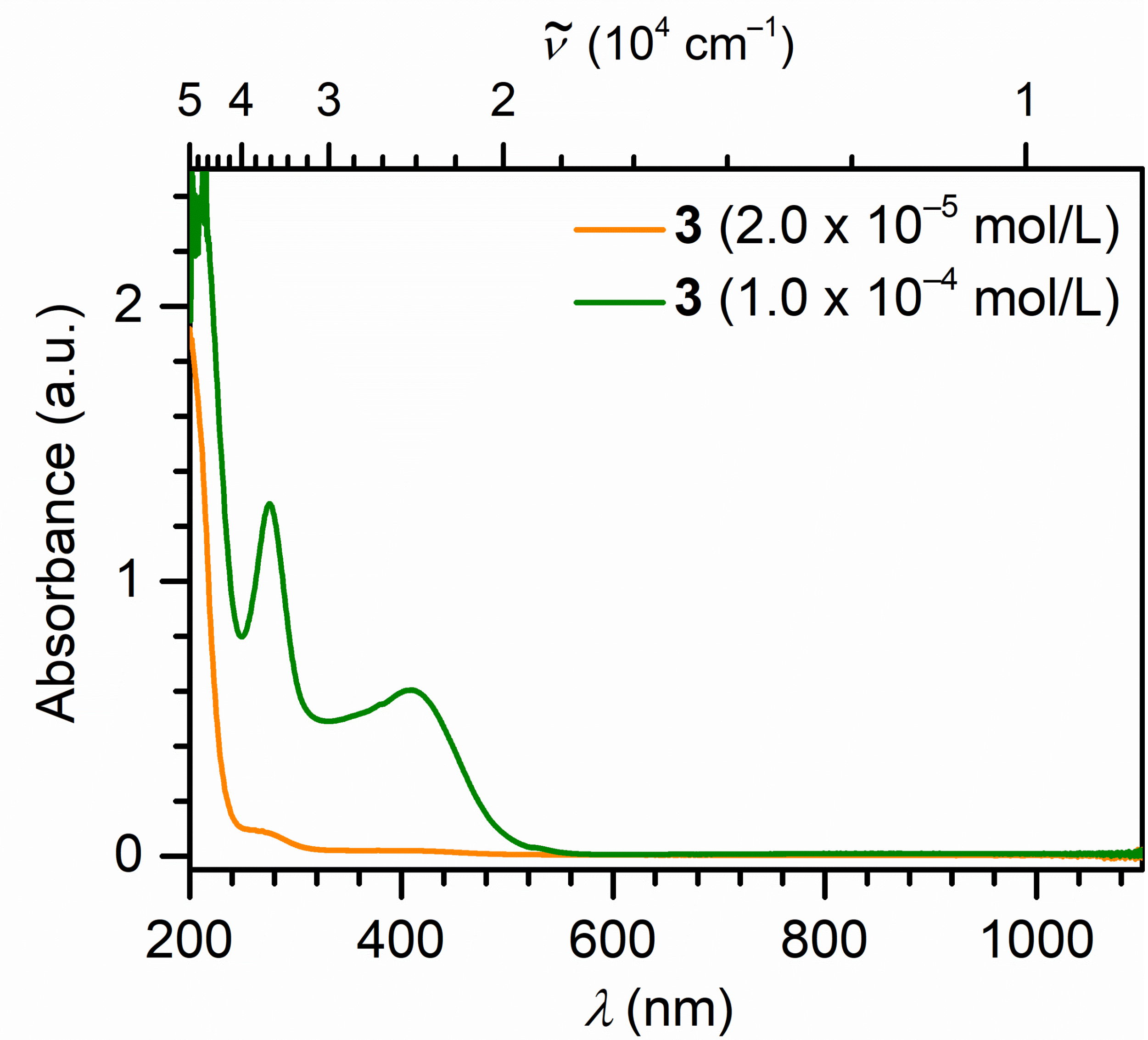

Figure S37. UV-vis spectra of $[\{(Me_3Si)_2NC(N^iPr)_2\}_2Er]_2(\mu\text{-}\eta^6\text{:}\eta^6\text{-}C_6H_5Me)$, **3**, recorded at 100 μmol/L (green trace) and 20 μmol/L (orange trace) concentrations in ^{n}hexane at room temperature.

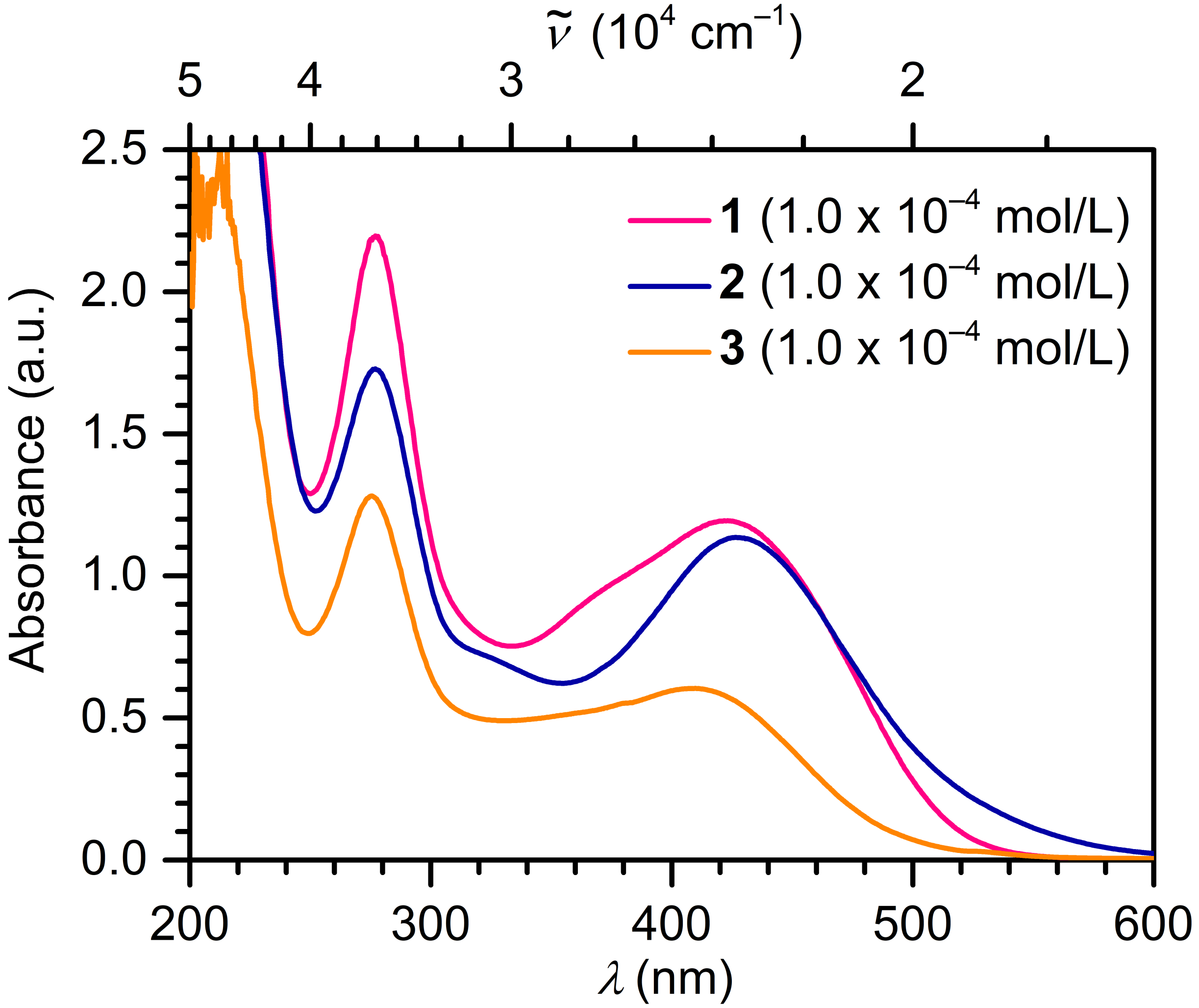

Figure S38. Magnification of the UV-vis spectra of $[\{(Me_3Si)_2NC(N^iPr)_2\}_2RE]_2(\mu\text{-}\eta^6\text{:}\eta^6\text{-}C_6H_5Me)$, where RE = Y (**1**, pink trace), Dy (**2**, blue trace), and Er (**3**, orange trace), recorded at 100 μmol/L concentrations in ^{n}hexane at room temperature.

5 Magnetic Measurements

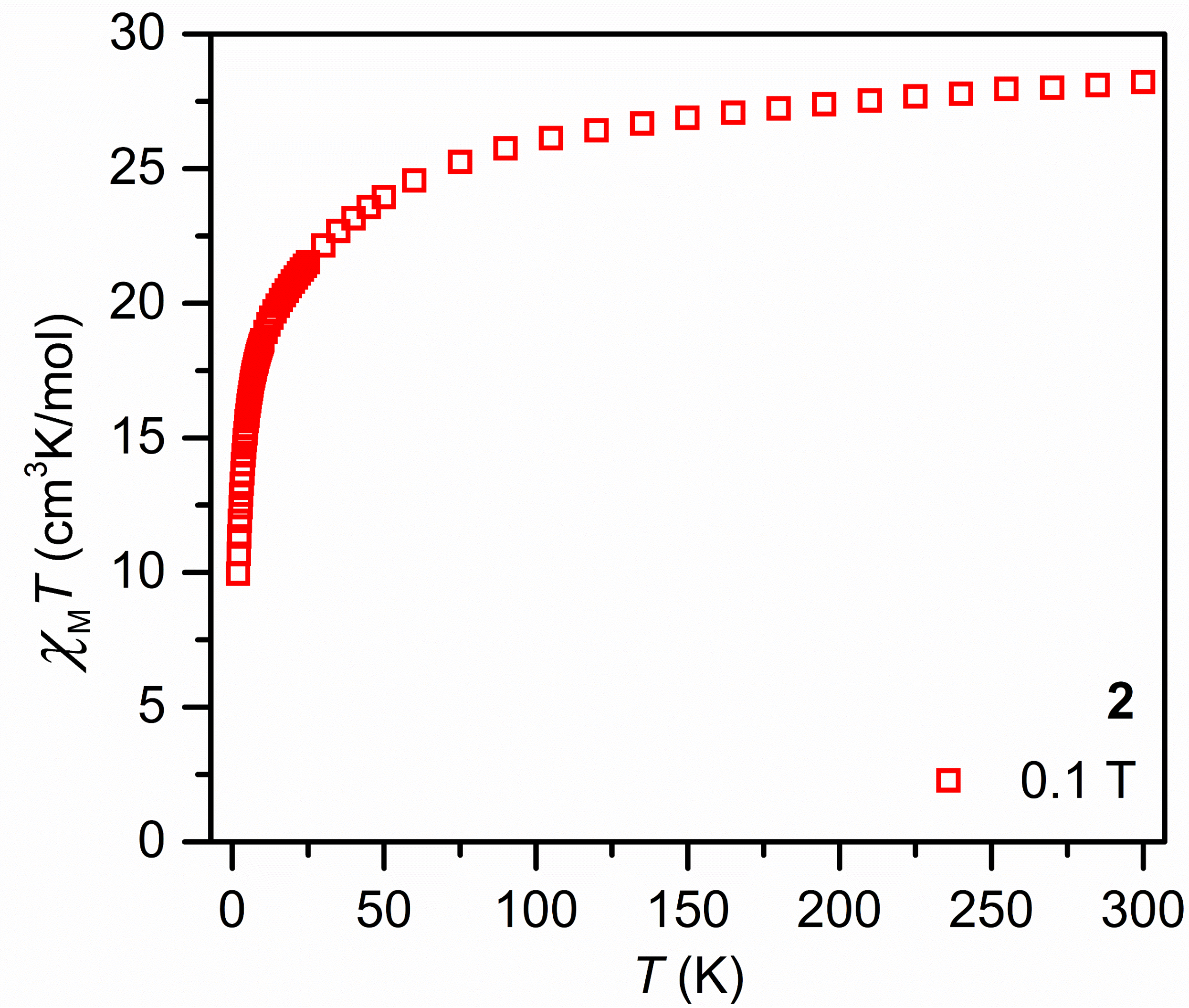

Figure S39. Variable-temperature dc magnetic susceptibility data for a restrained polycrystalline sample of $[\{(Me_3Si)_2NC(N^iPr)_2\}_2Dy]_2(\mu\text{-}\eta^6\text{:}\eta^6\text{-}C_6H_5Me)$, **2** (red squares), collected under a 0.1 T applied dc field.

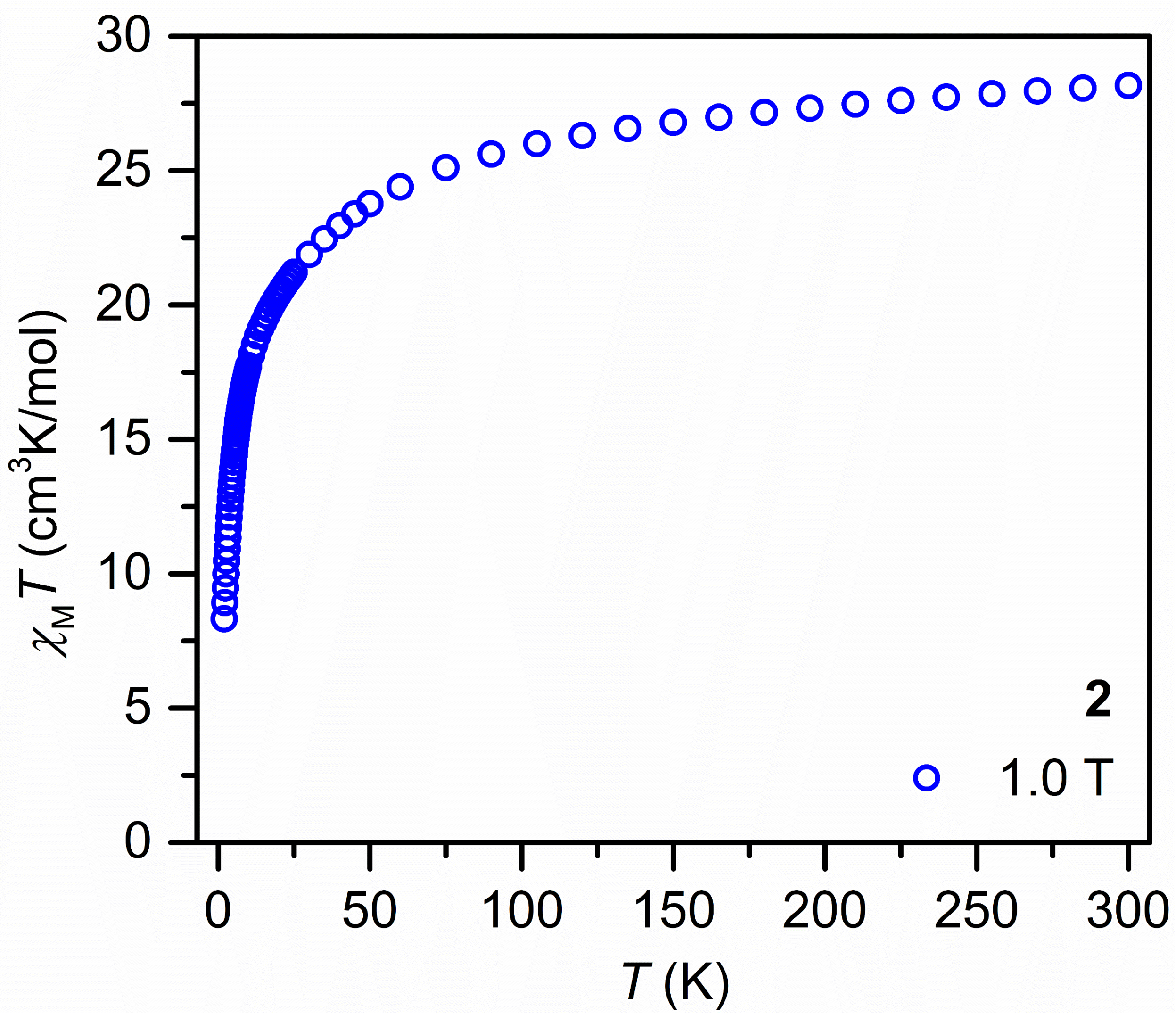

Figure S40. Variable-temperature dc magnetic susceptibility data for a restrained polycrystalline sample of $[\{(Me_3Si)_2NC(N^iPr)_2\}_2Dy]_2(\mu\text{-}\eta^6\text{:}\eta^6\text{-}C_6H_5Me)$, **2** (blue circles), collected under a 1.0 T applied dc field.

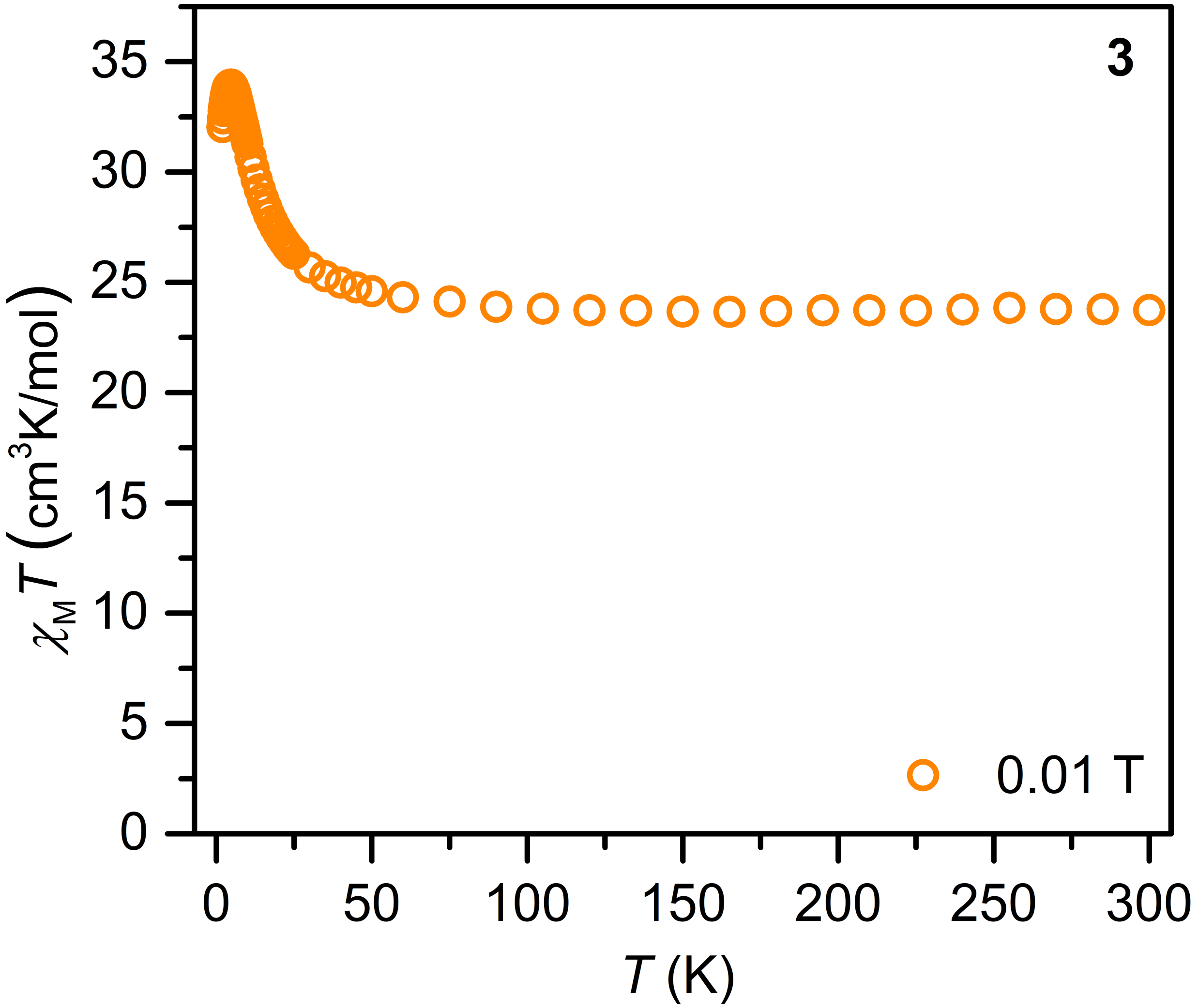

Figure S41. Variable-temperature dc magnetic susceptibility data for a restrained polycrystalline sample of $[\{(Me_3Si)_2NC(N^iPr)_2\}_2Er]_2(\mu\text{-}\eta^6\text{:}\eta^6\text{-}C_6H_5Me)$, **3** (orange circles), collected under a 0.01 T applied dc field.

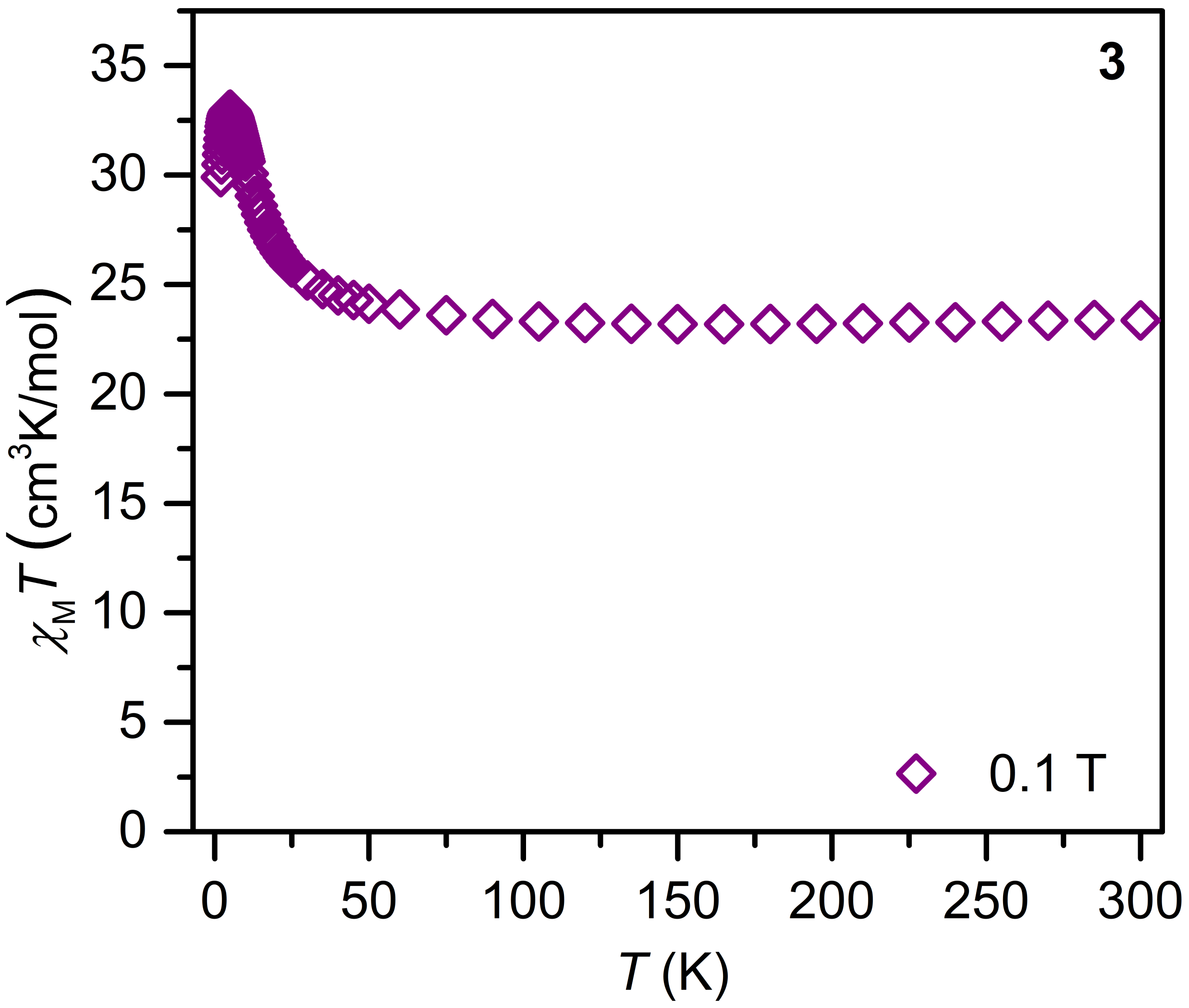

Figure S42. Variable-temperature dc magnetic susceptibility data for a restrained polycrystalline sample of $[\{(Me_3Si)_2NC(N^iPr)_2\}_2Er]_2(\mu\text{-}\eta^6\text{:}\eta^6\text{-}C_6H_5Me)$, **3** (purple diamonds), collected under a 0.1 T applied dc field.

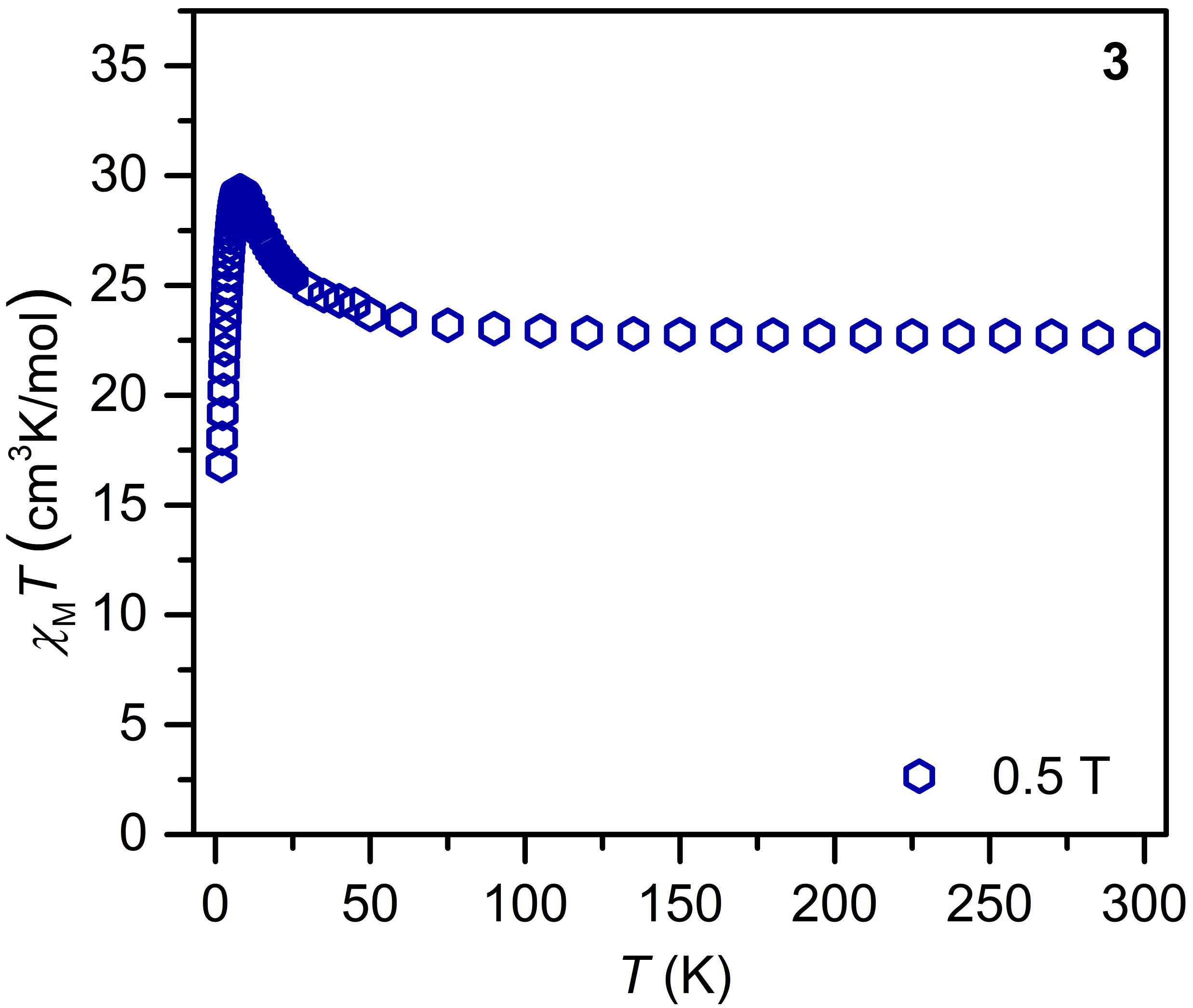

Figure S43. Variable-temperature dc magnetic susceptibility data for a restrained polycrystalline sample of $[\{(Me_3Si)_2NC(N^iPr)_2\}_2Er]_2(\mu\text{-}\eta^6\text{:}\eta^6\text{-}C_6H_5Me)$, **3** (dark blue hexagons), collected under a 0.5 T applied dc field.

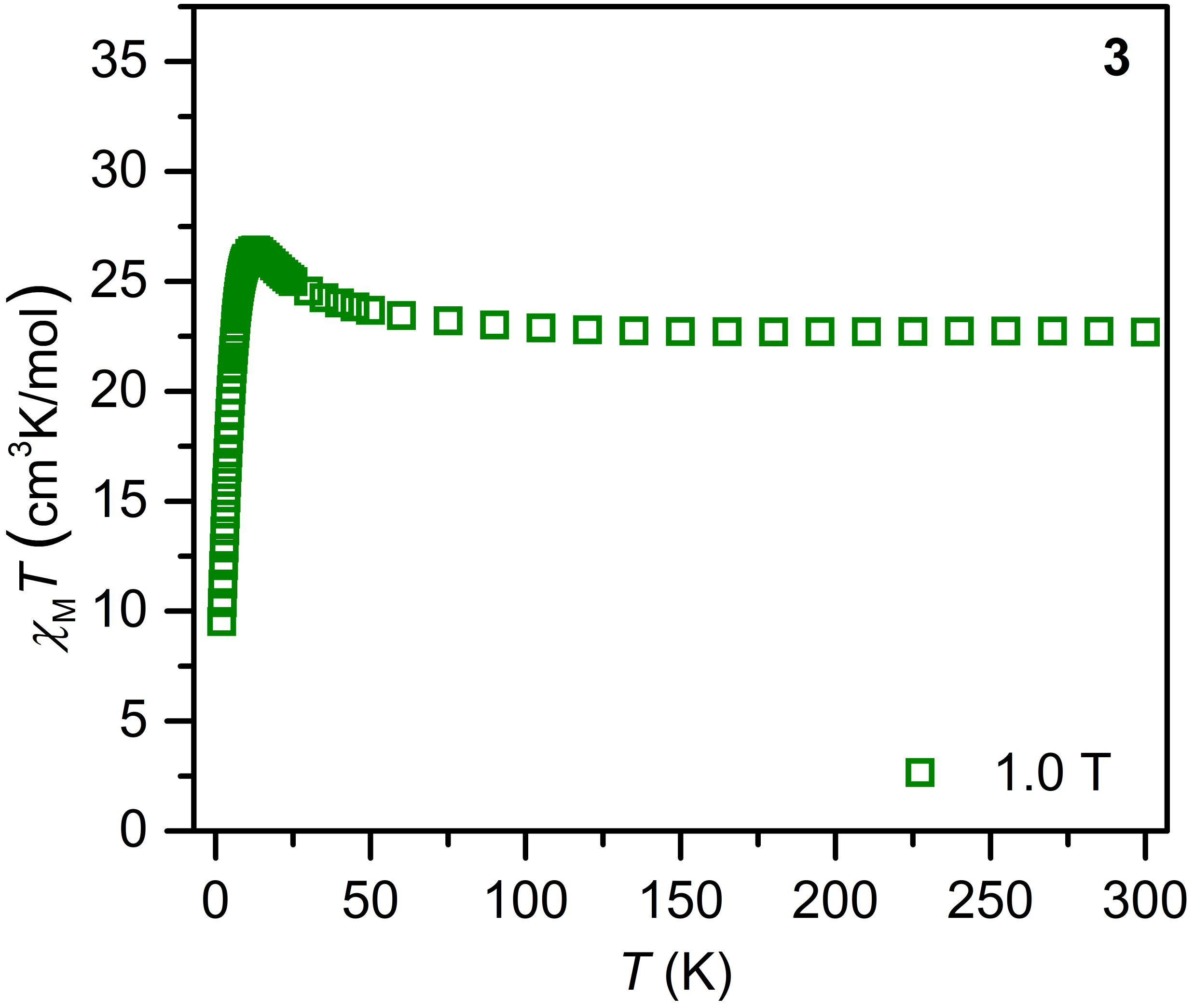

Figure S44. Variable-temperature dc magnetic susceptibility data for a restrained polycrystalline sample of $[\{(Me_3Si)_2NC(N^iPr)_2\}_2Er]_2(\mu\text{-}\eta^6\text{:}\eta^6\text{-}C_6H_5Me)$, **3** (green squares), collected under a 1.0 T applied dc field.

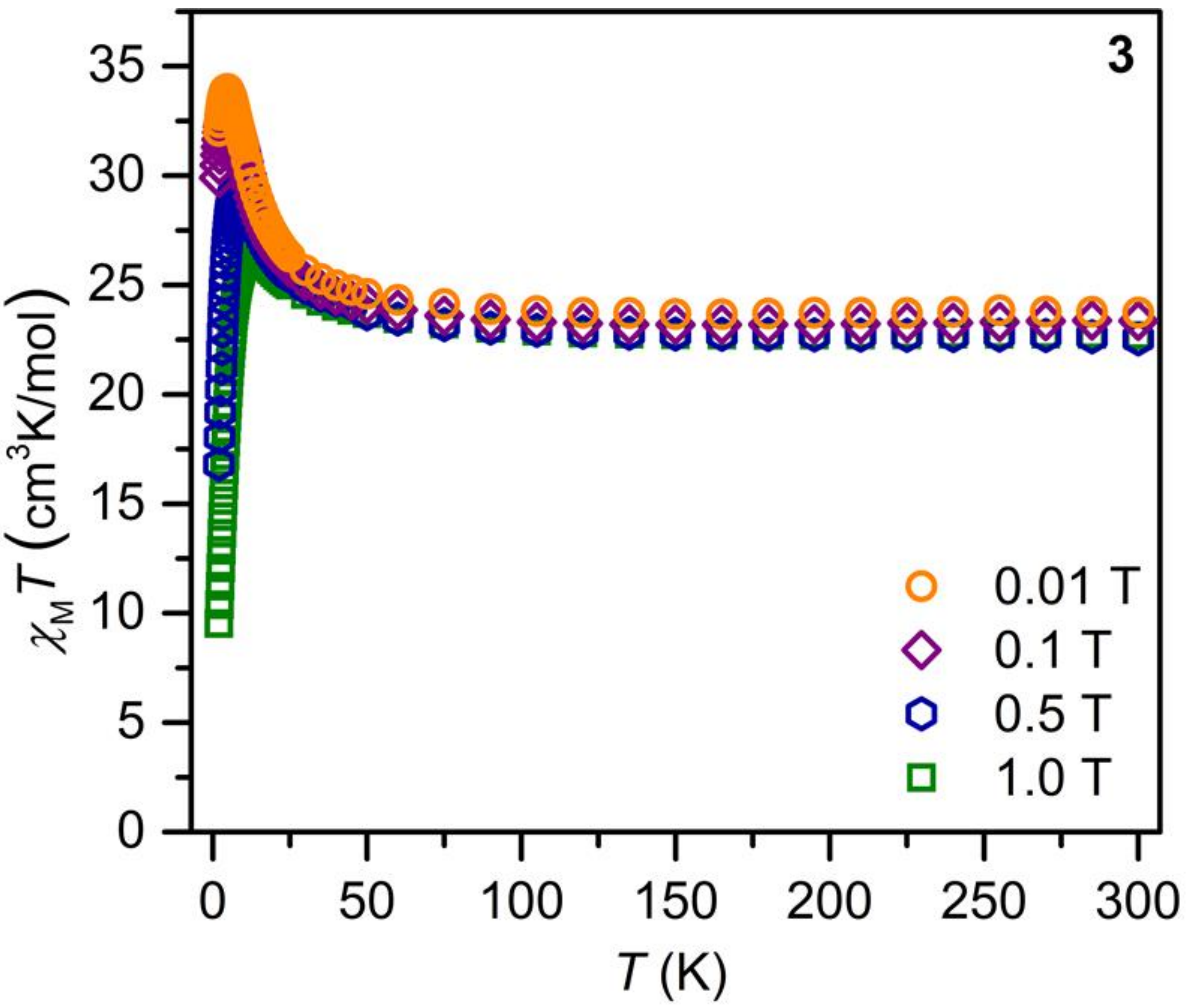

Figure S45. Variable-temperature dc magnetic susceptibility data for a restrained polycrystalline sample of $[\{(Me_3Si)_2NC(N^iPr)_2\}_2Er]_2(\mu\text{-}\eta^6\text{:}\eta^6\text{-}C_6H_5Me)$, **3** collected under 0.01 T, 0.1 T, 0.5 T, and 1.0 T applied dc fields.

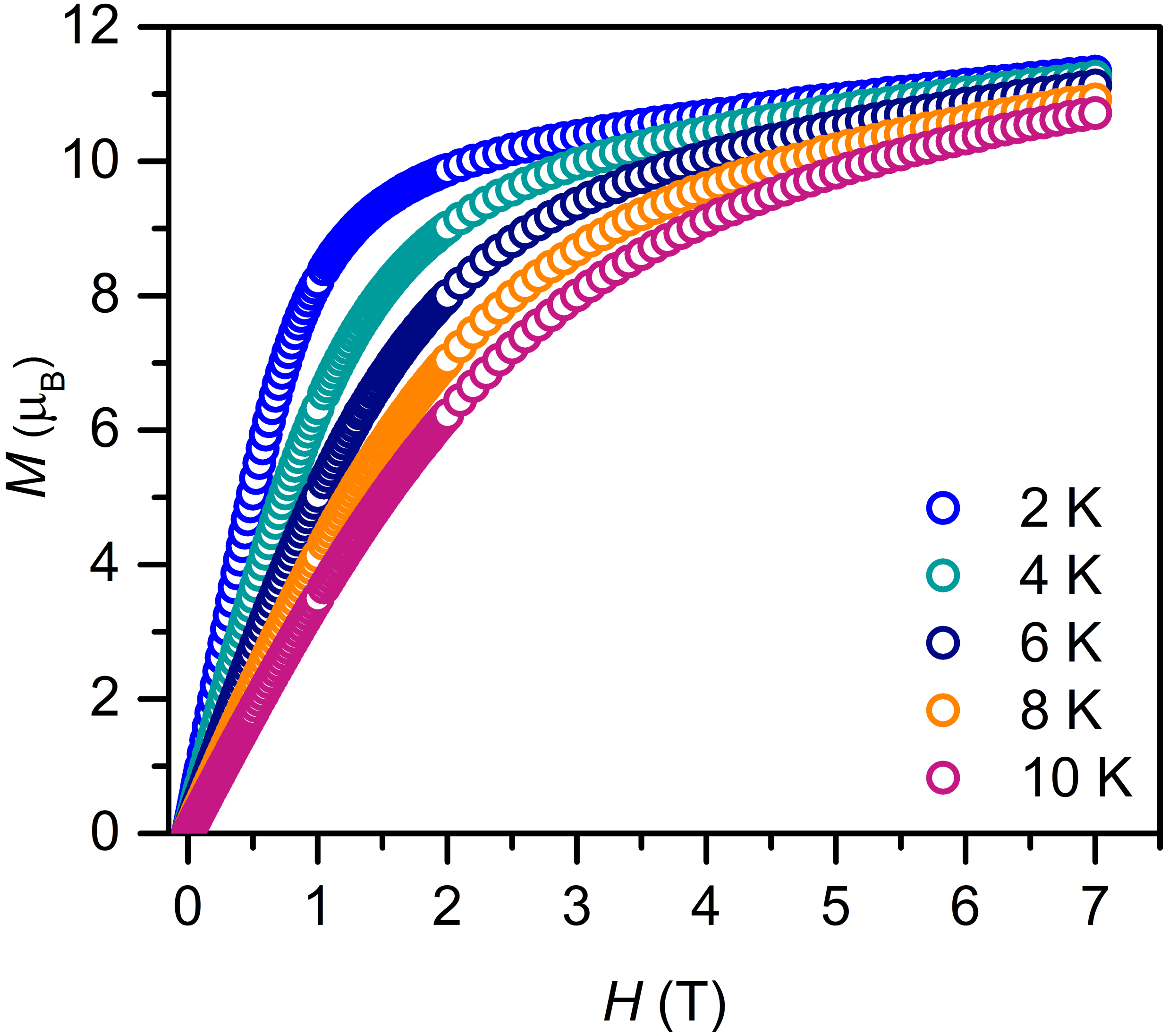

Figure S46. Variable-temperature field-dependent magnetization curves recorded for $[\{(Me_3Si)_2NC(N^iPr)_2\}_2Dy]_2(\mu\text{-}\eta^6\text{:}\eta^6\text{-}C_6H_5Me)$, **2**. Measurements were carried out from 0 to 7 T at 2, 4, 6, 8, and 10 K.

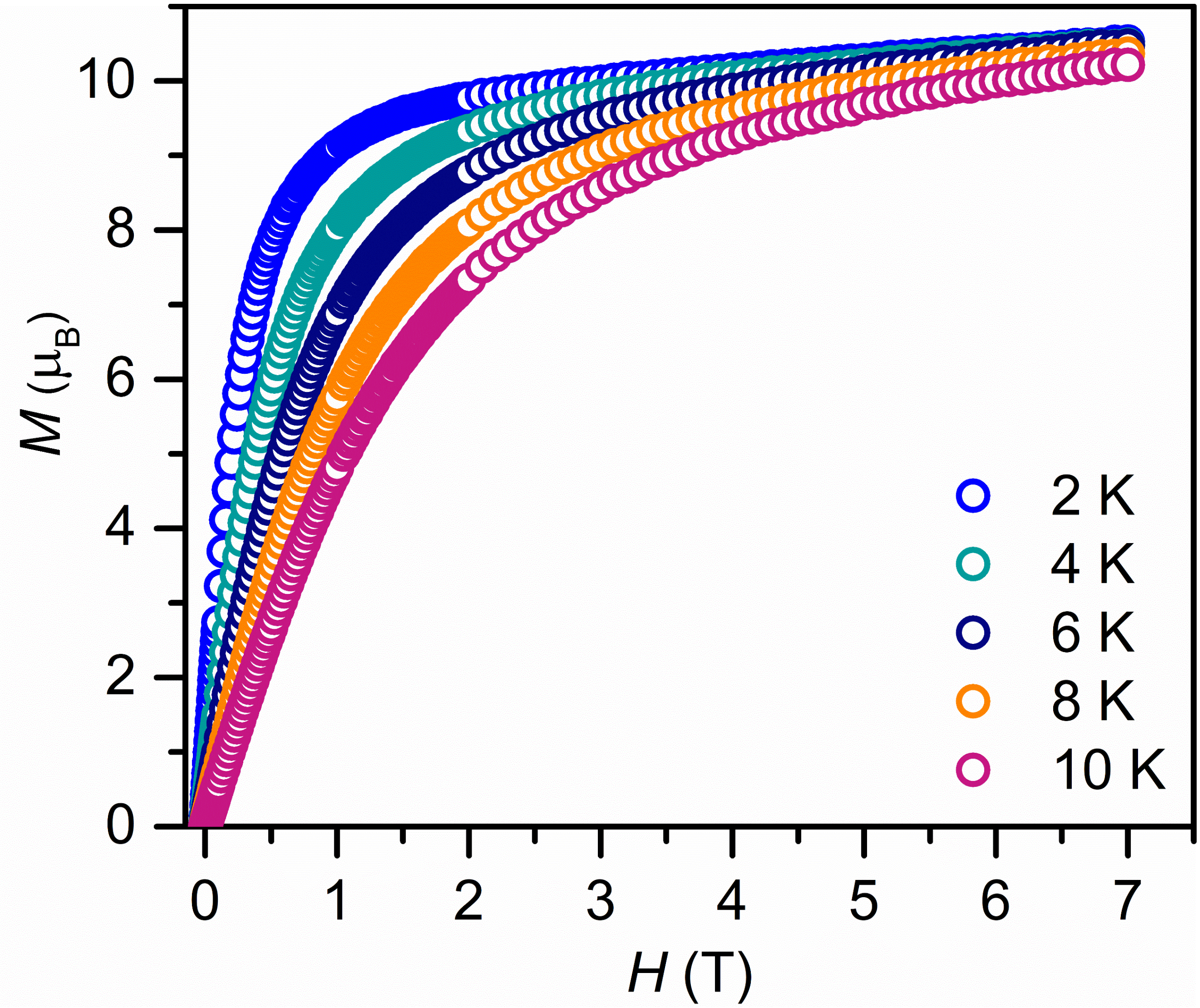

Figure S47. Variable-temperature field-dependent magnetization curves recorded for [{$(Me_3Si)_2NC(N^iPr)_2$}$_2$Er]$_2$(μ-η^6:η^6-C_6H_5Me), **3**. Measurements were carried out from 0 to 7 T at 2, 4, 6, 8, and 10 K.

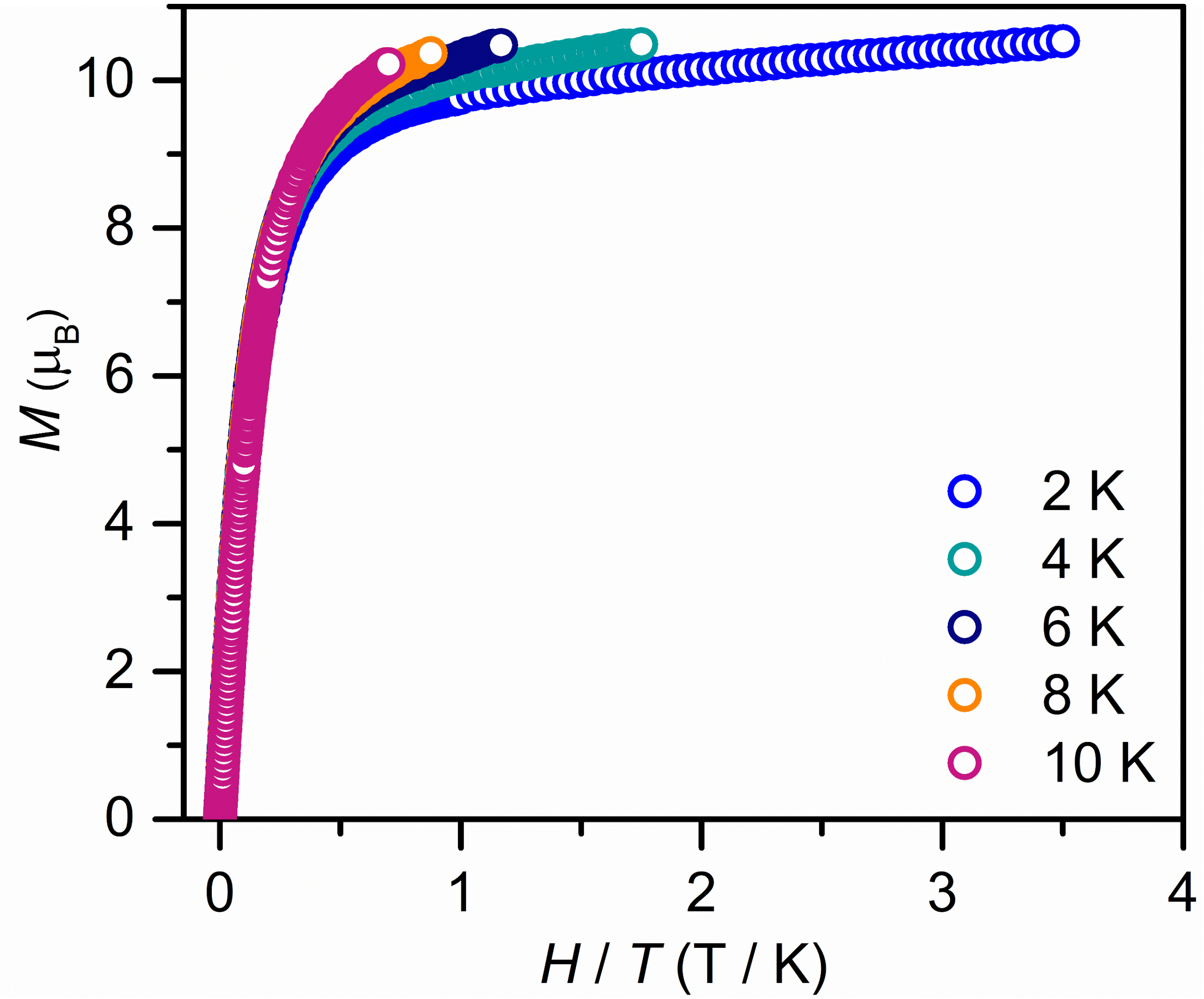

Figure S48. Variable-temperature field-dependent reduced magnetization curves recorded for [{$(Me_3Si)_2NC(N^iPr)_2$}$_2$Er]$_2$(μ-η^6:η^6-C_6H_5Me), **3**. Measurements were carried out from 0 to 7 T at 2, 4, 6, 8, and 10 K.

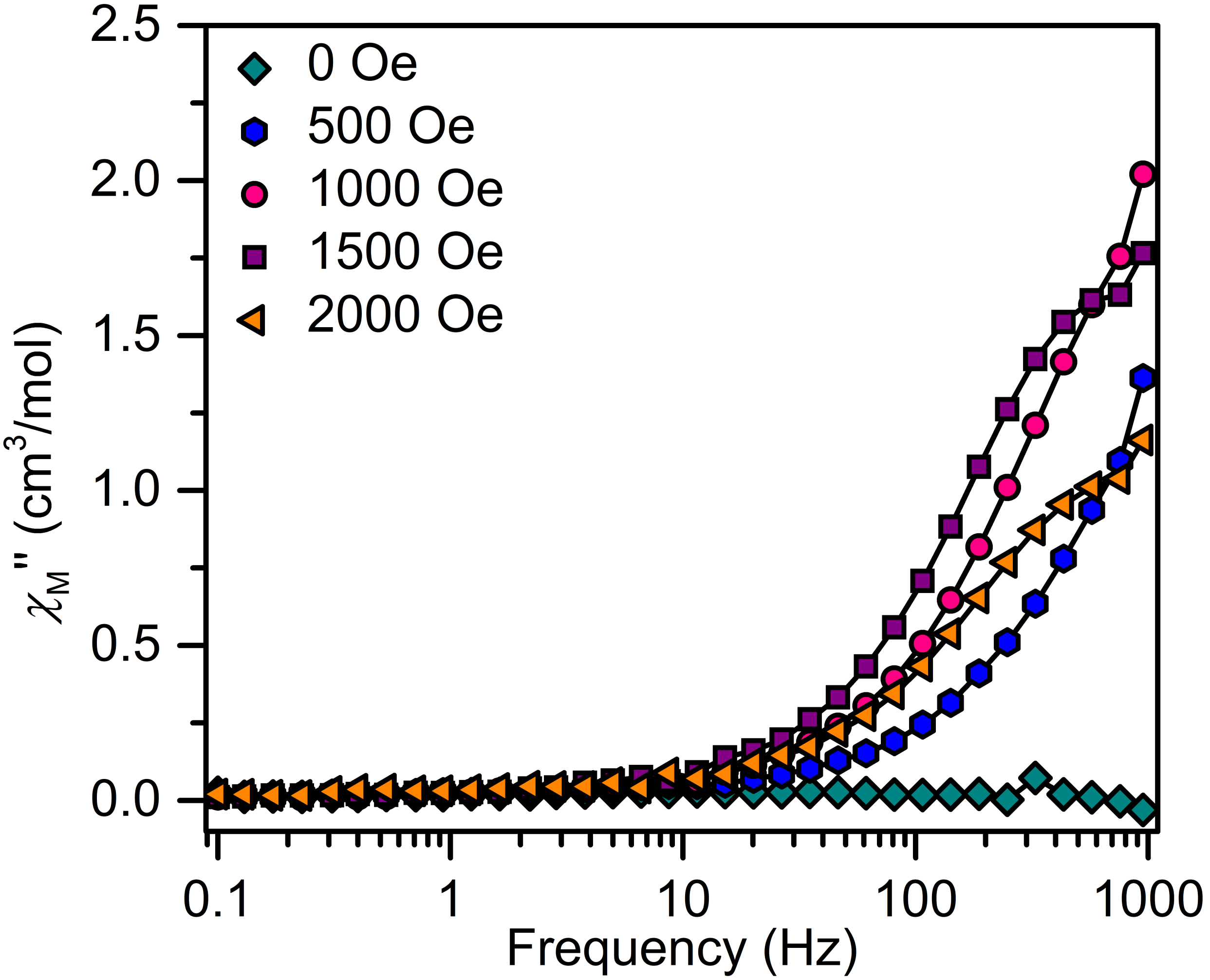

Figure S49. Out-of-phase (χ_M'') components of the ac magnetic susceptibility for [{$(Me_3Si)_2NC(N^iPr)_2$}$_2$Dy]$_2$(μ-η^6:η^6-C_6H_5Me), **2**, at 1.8 K under dc fields ranging from 0 Oe to 2000 Oe. Solid lines represent guides for the eye.

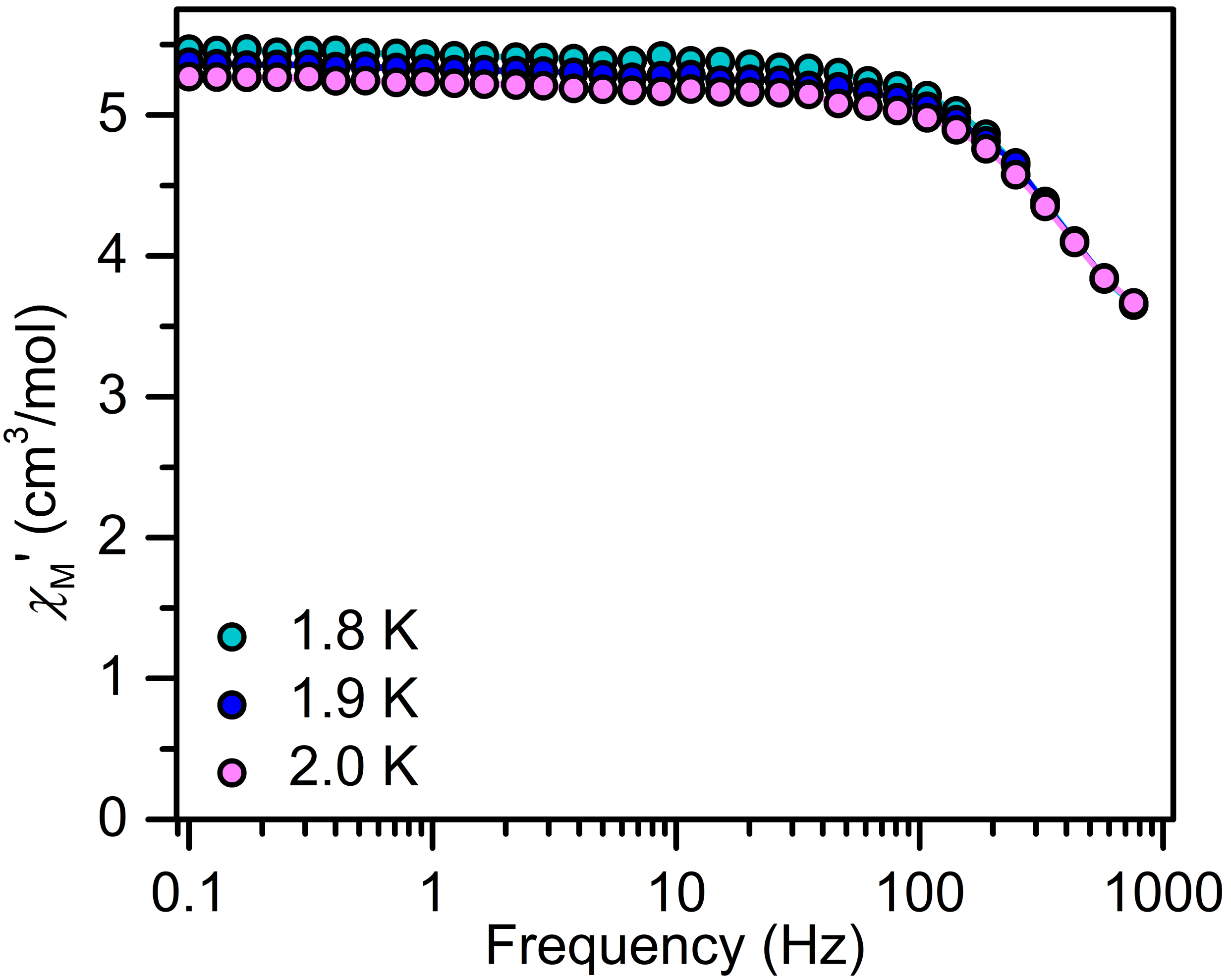

Figure S50. In-phase (χ_M') components of the ac magnetic susceptibility for $[\{(Me_3Si)_2NC(N^iPr)_2\}_2Dy]_2(\mu\text{-}\eta^6\text{:}\eta^6\text{-}C_6H_5Me)$, **2**, under a 1500 Oe applied dc field from 1.8 K (pale blue circles) to 2.0 K (pink circles). Solid lines represent guides for the eye.

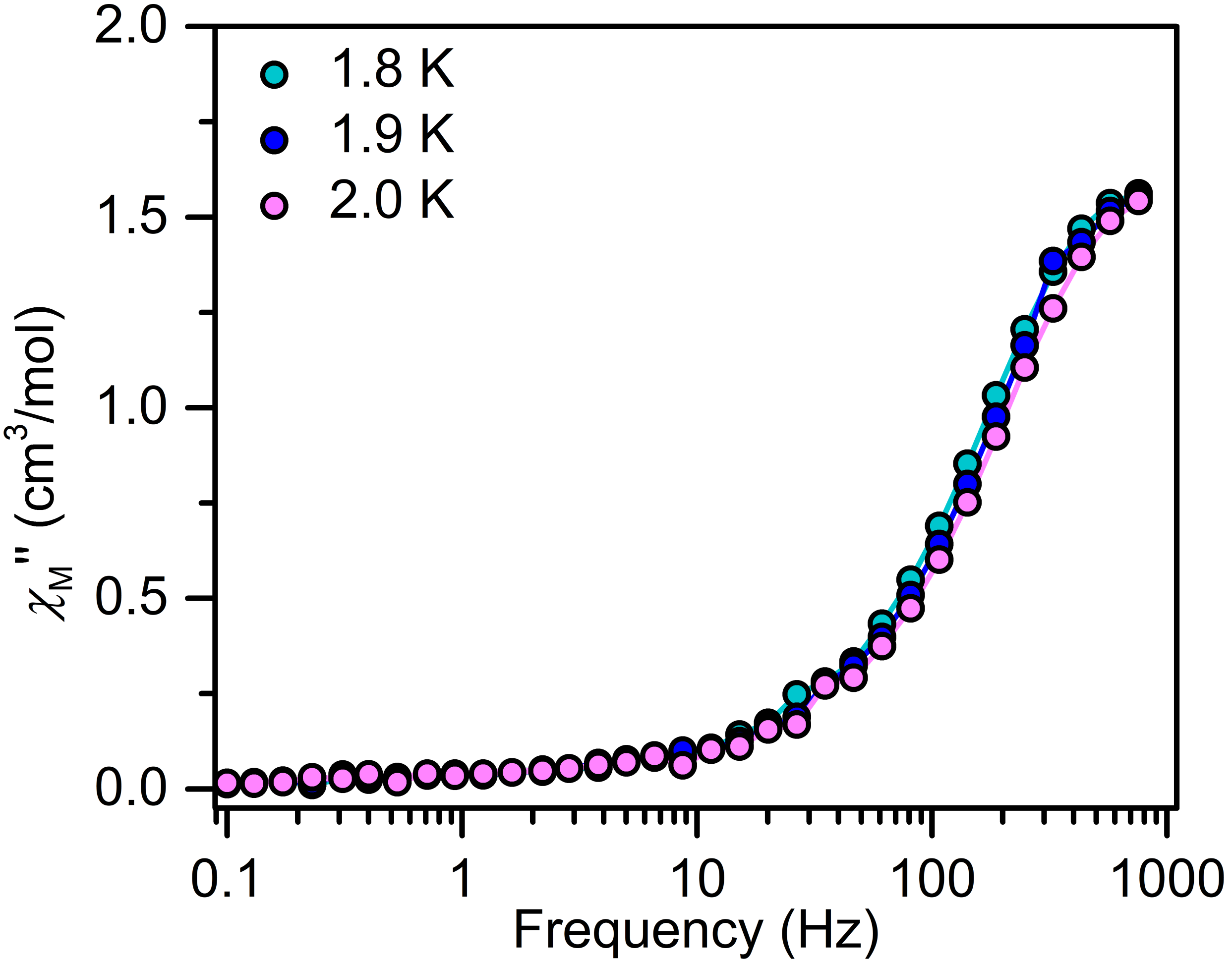

Figure S51. Out-of-phase (χ_M'') components of the ac magnetic susceptibility for $[\{(Me_3Si)_2NC(N^iPr)_2\}_2Dy]_2(\mu\text{-}\eta^6\text{:}\eta^6\text{-}C_6H_5Me)$, **2**, under a 1500 Oe applied dc field from 1.8 K (pale blue circles) to 2.0 K (pink circles). Solid lines represent guides for the eye.

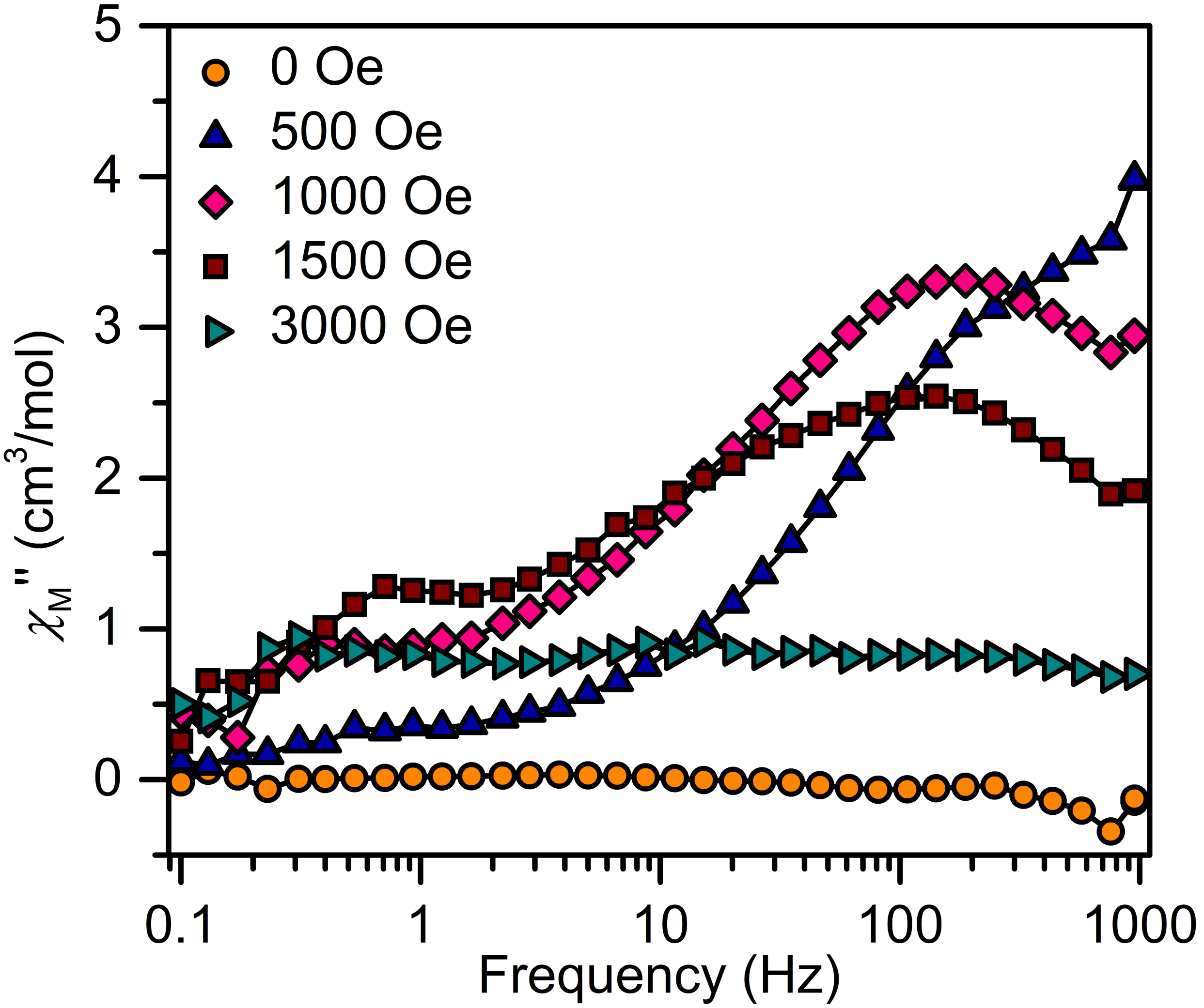

Figure S52. Out-of-phase (χ_M'') components of the ac magnetic susceptibility for [{(Me$_3$Si)$_2$NC(N^{i}Pr)$_2$}$_2$Er]$_2$(μ-η^6:η^6-C$_6$H$_5$Me), **3**, at 1.8 K under dc fields ranging from 0 Oe to 3000 Oe. Solid lines represent guides for the eye.

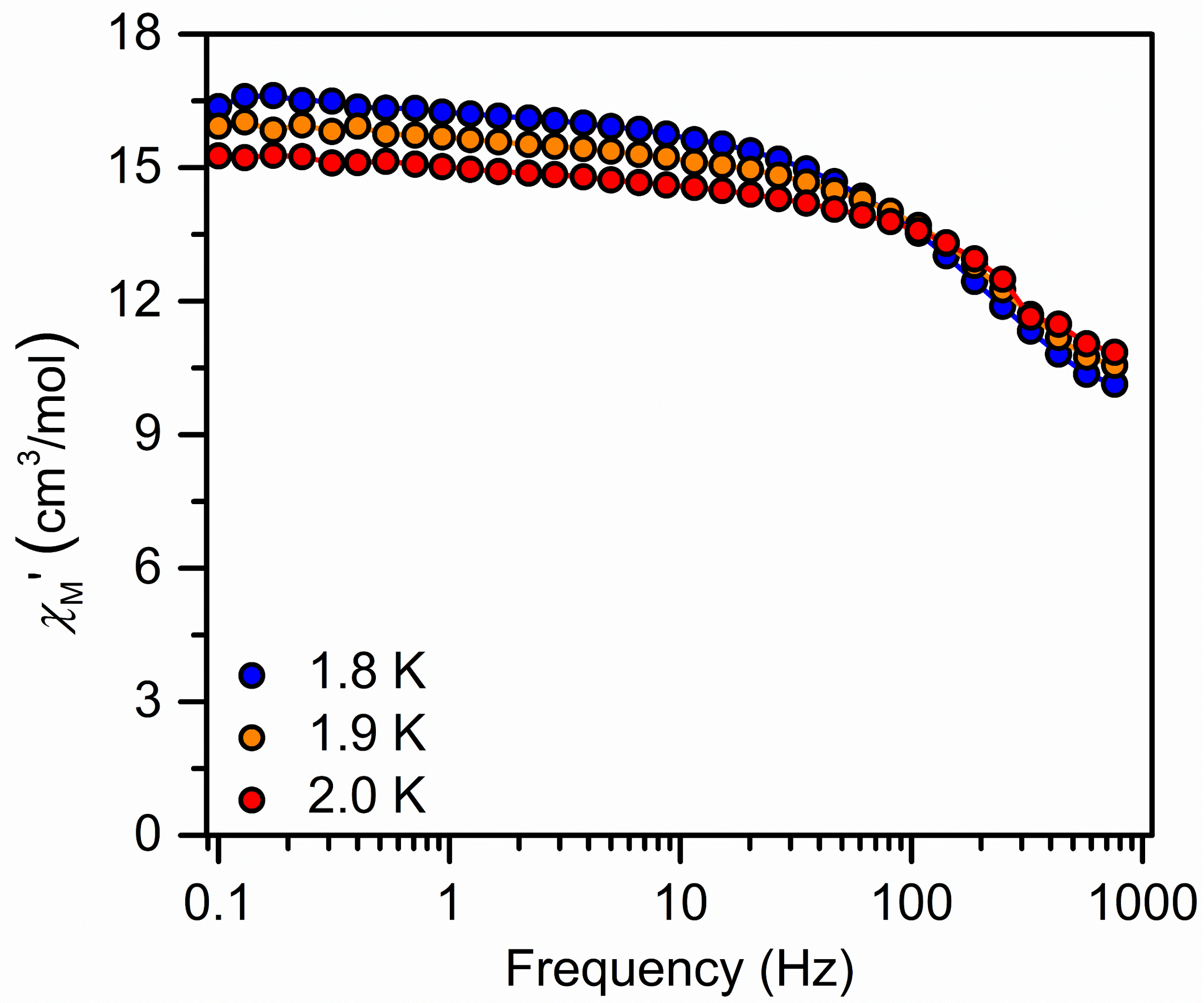

Figure S53. In-phase (χ_M') components of the ac magnetic susceptibility for [{(Me$_3$Si)$_2$NC(N^{i}Pr)$_2$}$_2$Er]$_2$(μ-η^6:η^6-C$_6$H$_5$Me), **3**, under a 500 Oe applied dc field from 1.8 K (blue circles) to 2.0 K (red circles). Solid lines represent guides for the eye.

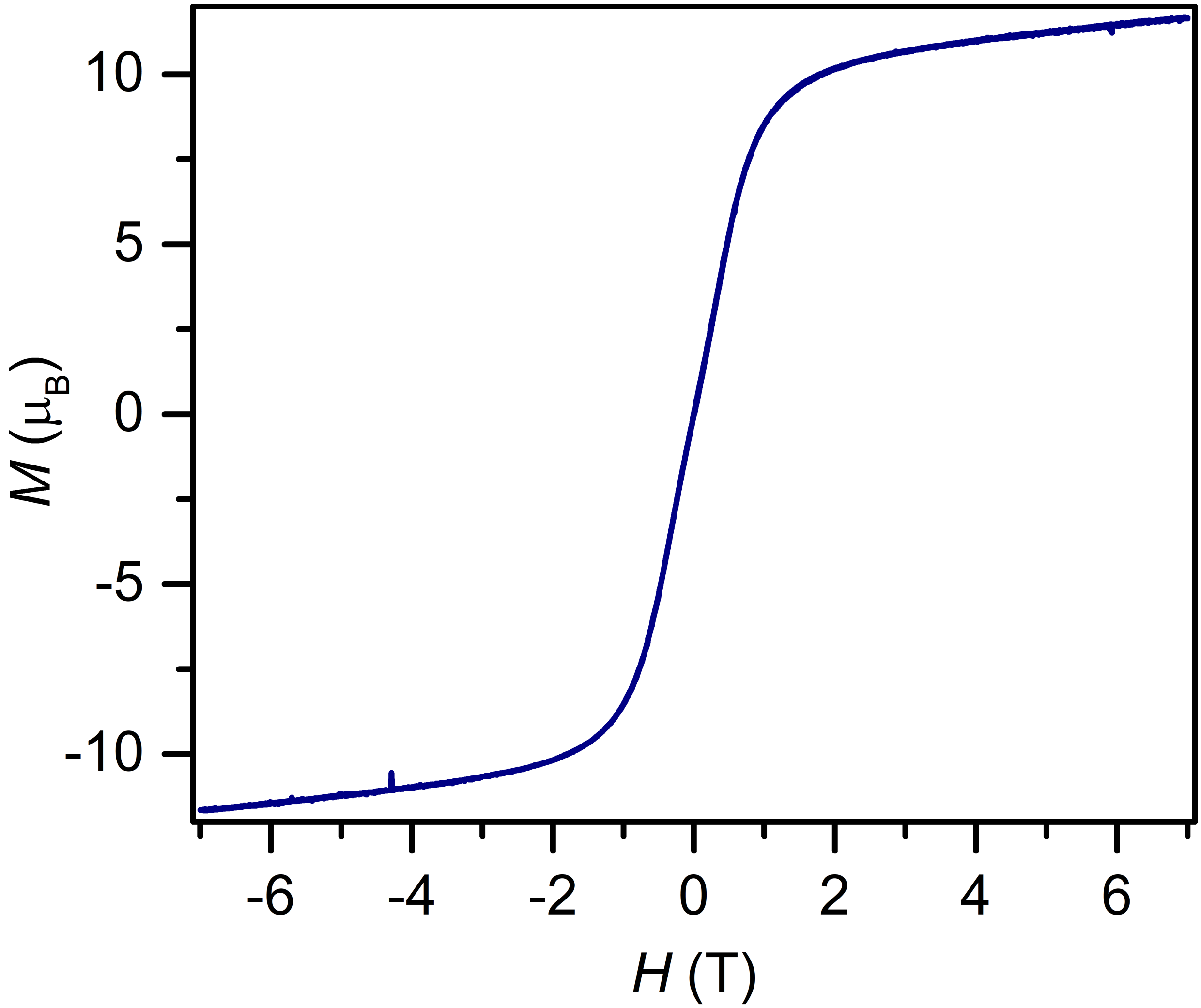

Figure S54. Plot of the magnetization (*M*) vs dc magnetic field (*H*) at an average sweep rate of 100 Oe/s for $[\{(Me_3Si)_2NC(N^iPr)_2\}_2Dy]_2(\mu\text{-}\eta^6\text{:}\eta^6\text{-}C_6H_5Me)$, **2**, at 1.8 K.

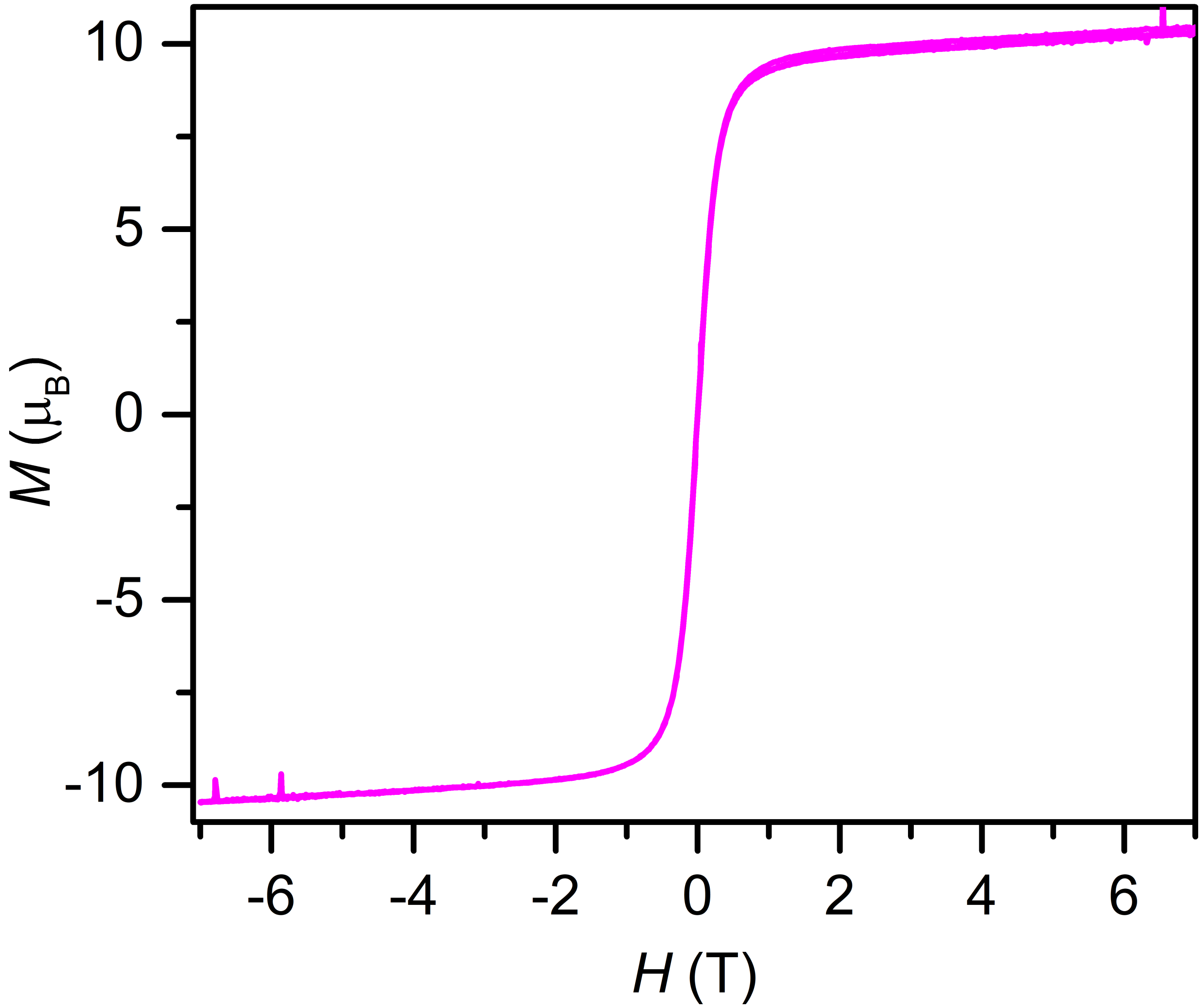

Figure S55. Plot of the magnetization (*M*) vs dc magnetic field (*H*) at an average sweep rate of 100 Oe/s for $[\{(Me_3Si)_2NC(N^iPr)_2\}_2Er]_2(\mu\text{-}\eta^6\text{:}\eta^6\text{-}C_6H_5Me)$, **3**, at 1.8 K.

6 DFT Calculations

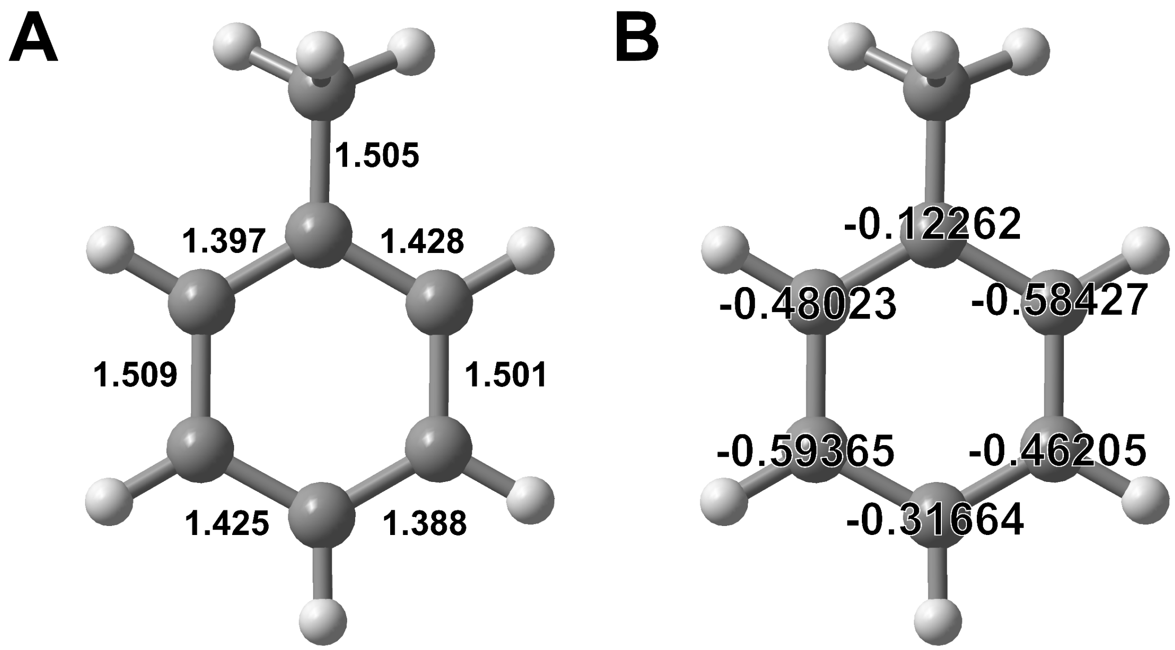

Figure S56. (**A**) DFT-calculated C–C bond distances of the bridging toluene unit of $[\{(Me_3Si)_2NC(N^iPr)_2\}_2Y]_2(\mu\text{-}\eta^6\text{:}\eta^6\text{-}C_6H_5Me)$, **1**. (**B**) Natural atomic charges of the arene ring of the bridging toluene unit of **1**. Gray and white-gray spheres represent C and H atoms, respectively.

Table S2. Bond metrics extracted from the geometry optimized coordinates of $[\{(Me_3Si)_2NC(N^iPr)_2\}_2Y]_2(\mu\text{-}\eta^6\text{:}\eta^6\text{-}C_6H_5Me)$, **1**. Optimization was performed employing the uTPPSSh functional at the def2-SVP level using the D3BJ correction.

	**Distances (Å)**
Y•••Y	4.502
Y–N	2.334, 2.353, 2.363, 2.364, 2.422, 2.440, 2.451, 2.453
Y–C_{guan}	2.823, 2.823, 2.826, 2.828
Y–C_{tol}	2.648, 2.649, 2.653, 2.655, 2.659, 2.667, 2.671, 2.673, 2.678, 2.703, 2.707, 2.719

Table S3. Majority contributions of the TD-DFT–calculated transition states for $[\{(Me_3Si)_2NC(N^iPr)_2\}_2Y]_2(\mu\text{-}\eta^6\text{:}\eta^6\text{-}C_6H_5Me)$, **1**, on the def2-TZVP level using the uB3LYP functional with the D3BJ dispersion correction and the hexane implicit solvent model. The calculated excitation energies were empirically shifted by 0.06 eV. Isovalue for all depictions is 0.03. Oscillator strength cutoff used is 0.02 and contributions higher than 15% are shown. (HOMO = 353, LUMO = 354)

λ (nm)	v (cm^{-1})	Oscillator Strength	Occupied	Virtual	Weight (%)
373.4	26771	0.32443	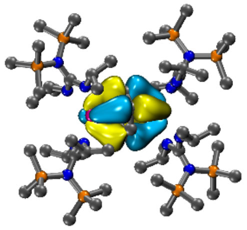 353α	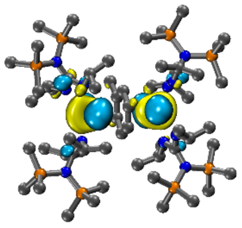 357α	34.8
			353β	357β	34.8
364.4	27444	0.10704	353α	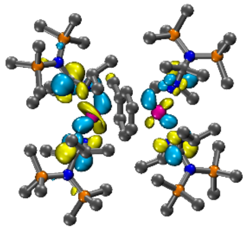 358α	40.6
			353β	358β	40.6
326.2	30656	0.06194	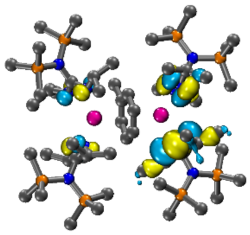 352α	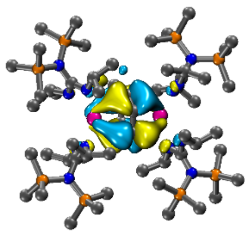 354α	45.4
			352β	354β	45.4
436.7	22902	0.03693	353α	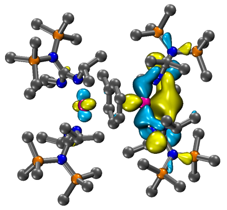 356α	31.9

			353β	356β	31.9
318.2	31425	0.03594	353α	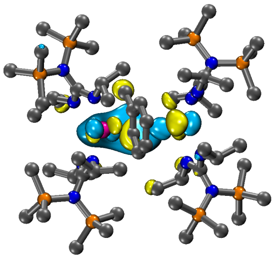 365α	26.3
			353β	365β	26.3
339.8	29426	0.03389	353α	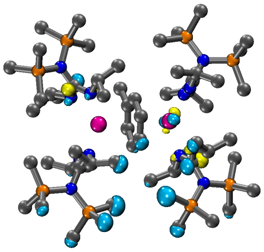 359α	40.4
			353β	359β	40.4
321.2	31138	0.02941	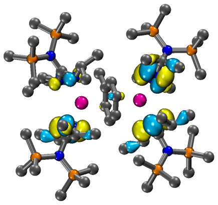 350α	354α	28.5
			350β	354β	28.5
304.1	32887	0.02533	353α	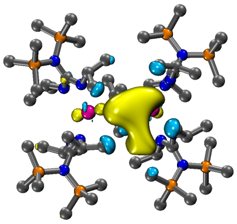 364α	49
			353β	364β	49

Table S4. Geometry-optimized coordinates of [{$(Me_3Si)_2NC(N^iPr)_2$}$_2Y]_2(\mu-\eta^6:\eta^6-C_6H_5Me)$, **1**. Optimization was performed using the uTPPSSh functional at the def2-SVP level. Calculation employed D3BJ dispersion correction. (Final single point energy = -5378.578550585787 Hartrees)

Y	7.654540	8.281223	13.101804
N	6.942217	9.431228	11.178342
N	9.500909	6.859819	12.975075
N	8.720161	10.324478	12.266708
N	7.525333	5.944688	12.364841
C	7.861175	10.406489	11.250584
C	8.835070	5.776205	12.551305
C	5.575018	9.252303	14.528918
C	6.715064	9.915719	14.988767
C	5.490775	7.828607	14.601293
Si	6.771529	12.832763	10.587830
N	7.894519	11.480944	10.303688
C	7.534607	14.437526	9.954527
C	6.483915	12.974225	12.443478
C	5.103565	12.634168	9.722576
Si	8.975985	11.349386	8.895599
C	9.852479	9.670360	8.937799
C	10.294461	12.702651	8.860928
C	7.945372	11.508902	7.320953
Si	9.973189	4.113141	10.658019
N	9.505270	4.528145	12.325151
C	9.286499	2.418516	10.182014
C	9.273541	5.419777	9.499689
C	11.849630	4.014058	10.442514
Si	9.805082	3.465593	13.719000
C	8.625634	1.990086	13.767265
C	11.555494	2.762307	13.652168
C	9.591716	4.474229	15.290942
C	6.056719	9.279841	10.032816
C	10.946049	6.891547	13.113894
C	9.657380	11.412364	12.528575
C	6.660838	4.808006	12.073742
H	9.354716	12.285181	11.924401
C	11.097813	11.068564	12.129191
C	9.617777	11.823530	13.999707
H	6.031940	10.223789	9.465665
C	6.567804	8.186721	9.087053
C	4.636520	8.977317	10.514555
C	5.862658	4.373180	13.308839
H	6.524559	4.168710	14.161226
H	5.282002	3.460332	13.098163

H	5.157524	5.163883	13.602646
H	7.291483	3.954147	11.771536
C	5.688494	5.107038	10.931194
H	11.326923	5.864325	13.262493
C	11.599950	7.451409	11.844050
C	11.316668	7.728737	14.341682
H	5.071023	5.986387	11.170203
H	5.015967	4.250215	10.766207
H	6.216348	5.309681	9.989489
H	7.574683	2.297201	13.868324
H	8.874093	1.350788	14.632378
H	8.709517	1.370140	12.861370
H	11.686316	2.040973	12.829612
H	11.760178	2.223919	14.593483
H	12.315357	3.551698	13.540518
H	11.502568	10.270157	12.767139
H	11.745751	11.953187	12.242575
H	11.157240	10.731565	11.085890
H	10.408977	5.198076	15.421346
H	9.576611	3.809406	16.169483
H	8.645451	5.032833	15.266592
H	9.734829	1.610396	10.782927
H	9.520449	2.207194	9.124537
H	8.192699	2.368217	10.303762
H	8.183830	5.318347	9.398238
H	9.720760	5.303024	8.498604
H	9.479662	6.442879	9.847393
H	11.379268	6.812316	10.978750
H	12.695324	7.520515	11.949990
H	11.203333	8.451571	11.628558
H	12.371955	4.881932	10.871468
H	12.087087	3.976139	9.365202
H	12.268419	3.106770	10.903253
H	10.945664	9.805983	8.956081
H	9.609359	9.078263	8.041093
H	9.577439	9.073682	9.819901
H	7.528158	8.473482	8.639368
H	5.850892	7.997643	8.271075
H	6.724950	7.253134	9.641921
H	9.879479	13.698318	8.647169
H	11.022237	12.468395	8.064503
H	10.846952	12.759101	9.811479
H	8.606227	12.130520	14.290989
H	10.303291	12.664962	14.190101
H	9.921252	10.983339	14.642800
H	8.511222	14.638946	10.421613

H	6.858144	15.270409	10.212285
H	7.664824	14.449930	8.860582
H	4.632207	8.087008	11.163907
H	3.959624	8.791302	9.665735
H	4.237611	9.818658	11.098734
H	10.884917	8.738281	14.257588
H	12.409223	7.823760	14.444907
H	10.918951	7.275720	15.262071
H	7.180679	10.717804	7.262261
H	8.589042	11.427624	6.428567
H	7.429962	12.482138	7.267653
H	6.399989	11.977051	12.901603
H	5.554909	13.529097	12.653498
H	7.314573	13.500522	12.938497
H	5.219975	12.429366	8.646245
H	4.529231	13.571329	9.825677
H	4.499900	11.824158	10.159047
C	4.387709	10.015496	14.006242
H	4.696192	10.911674	13.453824
H	3.778112	9.380581	13.350836
H	3.766650	10.335565	14.853668
H	6.767662	10.999009	14.943985
C	7.861318	9.147974	15.600622
H	4.606665	7.309196	14.243157
C	7.745034	7.730348	15.692127
C	6.613093	7.064993	15.241944
Y	5.764580	8.683838	17.168224
H	8.746075	9.666545	15.956758
H	8.557595	7.159836	16.137666
H	6.532047	5.988645	15.328508
N	6.609755	7.259653	18.853655
N	4.079381	10.236339	17.749643
N	4.658067	6.631103	17.886576
N	6.221914	10.883706	18.073552
C	5.623931	6.352738	18.763792
C	4.927378	11.172927	18.202726
C	7.745942	7.077487	19.746452
C	2.654921	10.292003	18.039377
C	3.588862	5.672483	17.626683
C	7.243725	11.887721	18.333950
N	5.609233	5.163808	19.567324
N	4.461113	12.398464	18.784380
H	7.702803	6.063118	20.172647
C	7.711603	8.066627	20.916717
C	9.055149	7.199035	18.959516
H	2.353365	11.342264	18.203734

C	2.343022	9.506540	19.318985
C	1.861518	9.744811	16.850275
H	3.900524	4.687636	18.015374
C	2.272664	6.027072	18.330373
C	3.343041	5.536014	16.123511
C	7.706237	12.575791	17.042926
H	6.818763	12.667933	18.989823
C	8.454477	11.276046	19.038944
Si	6.453437	3.711360	18.981809
Si	4.862343	5.261117	21.183110
Si	4.425077	12.543774	20.558987
Si	3.980268	13.718919	17.692584
H	6.842538	7.886247	21.563759
H	8.620861	7.977136	21.533346
H	7.642624	9.096231	20.543650
H	9.124991	8.185407	18.473805
H	9.928091	7.076735	19.620404
H	9.106579	6.429195	18.174478
H	2.861517	9.946668	20.181083
H	1.262198	9.493081	19.537116
H	2.695949	8.473186	19.214411
H	2.224152	8.741121	16.575977
H	0.788651	9.672865	17.088641
H	1.971012	10.392877	15.967970
H	1.838359	6.947539	17.915200
H	1.539046	5.215195	18.196607
H	2.422770	6.178254	19.407319
H	4.260900	5.239839	15.599481
H	2.567740	4.779064	15.923292
H	3.004458	6.494840	15.700695
H	6.854130	12.952021	16.459841
H	8.372278	13.424736	17.268462
H	8.263431	11.869566	16.414569
H	8.855268	10.437369	18.448403
H	9.251897	12.026149	19.160140
H	8.192563	10.894297	20.034846
C	5.540446	2.165120	19.558941
C	6.491739	3.754817	17.106574
C	8.238589	3.567872	19.589337
C	4.237183	7.019716	21.466572
C	3.397718	4.083732	21.383956
C	6.145060	4.799667	22.491345
C	5.296167	14.123096	21.122049
C	5.310759	11.059754	21.299938
C	2.660177	12.641861	21.229954
C	5.194454	15.166396	17.734826

C	2.291881	14.407085	18.184155
C	3.892911	13.041002	15.941092
H	4.481270	2.176481	19.258105
H	6.016373	1.286133	19.091446
H	5.588428	2.019455	20.649817
H	6.921657	4.707280	16.765919
H	7.124645	2.940461	16.719304
H	5.491281	3.651866	16.663410
H	8.318697	3.659019	20.683954
H	8.635844	2.577422	19.305993
H	8.891878	4.329167	19.136218
H	3.165514	7.106505	21.232647
H	4.368852	7.297012	22.525507
H	4.775221	7.756457	20.853990
H	3.703137	3.033304	21.496205
H	2.840629	4.366420	22.294254
H	2.702116	4.148598	20.533160
H	7.015252	5.474526	22.466331
H	5.698336	4.856518	23.498599
H	6.511852	3.769074	22.353529
H	4.741176	15.030051	20.832692
H	5.368817	14.122940	22.223224
H	6.316963	14.201361	20.715511
H	6.397205	11.133872	21.149128
H	5.119758	11.018502	22.385103
H	4.978919	10.108009	20.860609
H	2.022412	11.820996	20.870945
H	2.688058	12.590787	22.332268
H	2.173391	13.590419	20.956714
H	6.225127	14.856066	17.509273
H	4.894043	15.912305	16.978515
H	5.200528	15.671639	18.712369
H	2.326284	14.946165	19.144563
H	1.954671	15.126462	17.418314
H	1.531162	13.614315	18.263916
H	3.018980	12.385996	15.809972
H	3.807778	13.873043	15.222551
H	4.788941	12.457377	15.683324

7 References

(1) Kelly, R. P.; Toniolo, D.; Tirani, F. F.; Maron, L.; Mazzanti, M. A Tetranuclear Samarium(II) Inverse Sandwich from Direct Reduction of Toluene by a Samarium(II) Siloxide. *Chem. Commun.* **2018**, *54* (73), 10268–10271. https://doi.org/10.1039/C8CC04169E.

(2) Gun'ko, Y. K.; Hitchcock, P. B.; Lappert, M. F. Nonclassical Organolanthanoid Metal Chemistry: [K([18]-Crown-6)(η^2-PhMe)$_2$]X (X = [(LnCp$^t{}_3$)$_2$(μ-H)], [(LnCp“$_2$)$_2$(μ-η^6:η^6-PhMe)]) from [LnCp$^x{}_3$], K, and [18]-Crown-6 in Toluene (Ln = La, Ce; Cp^t = η^5-$C_5H_4SiMe_2Bu^t$; Cp“ = η^5-$C_5H_3(SiMe_3)_2$-1,3). *Organometallics* **2000**, *19* (15), 2832–2834. https://doi.org/10.1021/om0001873.

(3) Kelly, R. P.; Maron, L.; Scopelliti, R.; Mazzanti, M. Reduction of a Cerium(III) Siloxide Complex To Afford a Quadruple-Decker Arene-Bridged Cerium(II) Sandwich. *Angew. Chem. Int. Ed.* **2017**, *56* (49), 15663–15666. https://doi.org/10.1002/anie.201709769.

(4) Zhang, L.; Jiang, Z.; Zhang, C.; Cheng, K.; Li, S.; Gao, Y.; Wang, X.; Chu, J. Room Temperature Ring Opening of Benzene by Four-Electron Reduction and Carbonylation. *J. Am. Chem. Soc.* **2025**, *147* (28), 25017–25023. https://doi.org/10.1021/jacs.5c08414.

(5) Richardson, G. M.; Rajeshkumar, T.; Burke, F. M.; Cameron, S. A.; Nicholls, B. D.; Harvey, J. E.; Keyzers, R. A.; Butler, T.; Granville, S.; Liu, L.; Langley, J.; Lim, L. F.; Cox, N.; Chilton, N. F.; Hicks, J.; Davis, N. J. L. K.; Maron, L.; Anker, M. D. Four-Electron Reduction of Benzene by a Samarium(II)-Alkyl without the Addition of External Reducing Agents. *Nat. Chem.* **2025**, *17* (1), 20–28. https://doi.org/10.1038/s41557-024-01688-6.

(6) Jin, P.-B.; Luo, Q.-C.; Gransbury, G. K.; Winpenny, R. E. P.; Mills, D. P.; Zheng, Y.-Z. Rare Earth Benzene Tetraanion-Bridged Amidinate Complexes. *Chem. Sci.* **2025**, *16* (4), 1907–1924. https://doi.org/10.1039/D4SC05982D.
